# Polyurethane Recycling: Sustainable Development Perspectives and Innovative Approaches

**DOI:** 10.3390/ma19040805

**Published:** 2026-02-19

**Authors:** Konrad Polecki, Joanna Paciorek-Sadowska, Marcin Borowicz, Marek Isbrandt, Iwona Zarzyka

**Affiliations:** 1 Department of Chemistry and Technology of Polyurethanes, Faculty of Materials Engineering, Kazimierz Wielki University, JK Chodkiewicza Street 30, 85-064 Bydgoszcz, Poland; poleckikonrad7@gmail.com (K.P.); sadowska@ukw.edu.pl (J.P.-S.); m.borowicz@ukw.edu.pl (M.B.); 2 Department of Organic Chemistry, Faculty of Chemistry, Rzeszów University of Technology, Powstańców Warszawy 6, 35-959 Rzeszów, Poland; izarzyka@prz.edu.pl

**Keywords:** polyurethanes, polyurethane materials, application of polyurethanes, recycling of polyurethanes

## Abstract

**Highlights:**

**What are the main findings?**

- Recent advances in catalytic depolymerization, bio-based polyols and NIPU chemistry support transition to circular life cycles.
- Hybrid strategies show promise for improving material recovery and reducing environmental impact.

**What are the implications of the main findings?**

- Mechanical recycling remains feasible but reduces mechanical and insulation performance.
- Chemical recycling enables recovery of polyols suitable for new polyurethane systems.
- Biological routes show potential for selective cleavage of urethane and ester linkages.

**Abstract:**

Polyurethanes are widely used polymeric materials; their crosslinked structure and compositional diversity significantly hinder effective end-of-life management. The review emphasizes polyurethane recycling technologies, with chemical aspects discussed only insofar as they directly affect recyclability. The influence of polyol and isocyanate structure on phase separation, network architecture and thermal stability is discussed in the context of degradation and depolymerization mechanisms. Mechanical, chemical, thermochemical and emerging biological recycling routes are compared, with emphasis on their respective advantages, limitations and technological maturity. Mechanical recycling remains the most accessible option on an industrial scale but typically leads to reduced mechanical and thermal-insulation performance. Chemical recycling—particularly glycolysis, hydrolysis and aminolysis—enables partial recovery of polyols suitable for reuse in new polyurethane formulations, albeit at the cost of higher energy demand and increased process complexity. The environmental impact of polyurethane recycling is considered in terms of energy consumption, greenhouse-gas emissions, waste-reduction potential and alignment with circular-economy principles. Emerging biological and hybrid recycling strategies are highlighted as promising low-temperature alternatives with potential environmental benefits, despite their current low technological readiness. Key structural and technological barriers to efficient polyurethane recycling are identified, and future research directions toward improved sustainability and resource efficiency are outlined.

## 1. Introduction

Global plastic production is projected to reach approximately 600 million tons by 2060, and polymer synthesis currently accounts for nearly 3% of global greenhouse-gas emissions. A significant fraction of plastics is manufactured for short-lived or single-use applications, including packaging materials, shopping bags, agricultural films and consumer products. Despite increasing awareness of environmental consequences, only a limited share of plastic waste is effectively recycled, while substantial amounts are incinerated, landfilled or improperly managed, contributing to long-term environmental pollution and resource depletion [1,2].

In response to the escalating problem of plastic pollution, various mitigation strategies have been proposed, including material reduction, reuse, mechanical recycling, chemical recycling and the development of bio-based or biodegradable polymers. However, the feasibility and effectiveness of these approaches strongly depend on the chemical structure, application profile and service life of specific polymer classes [3].

Polyurethanes (PUs) constitute one of the most versatile and widely used classes of polymeric materials, with applications spanning construction, automotive, furniture, packaging, coatings, and thermal insulation. Their broad industrial relevance arises from the extraordinary tunability of their chemical structure, which enables precise control over mechanical, thermal, and physicochemical properties. However, this same structural diversity—combined with extensive crosslinking and formulation variability—poses significant challenges for end-of-life management [4,5].

In the context of sustainable development and circular-economy strategies, polyurethane recycling has emerged as a critical research and technological priority. Despite growing regulatory pressure and increasing environmental awareness, the effective recycling of polyurethane waste remains limited compared with other polymer classes. This limitation stems not only from the chemical stability of urethane linkages but also from aging-induced degradation, heterogeneous formulations, and the coexistence of thermoplastic and thermoset polyurethane systems [6].

Mechanical recycling and energy recovery currently dominate industrial practice due to their technological maturity and economic feasibility. However, these approaches often result in significant deterioration of functional properties or irreversible material loss. In contrast, chemical recycling routes—including glycolysis, hydrolysis, aminolysis, and emerging catalytic depolymerization strategies—offer the potential for higher-value material recovery, particularly through the regeneration of polyols suitable for reuse in new polyurethane formulations [7,8].

**Figure 1:**
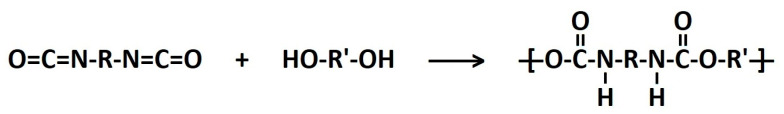
Schematic representation of polyurethane formation via the polyaddition reaction between a bifunctional isocyanate and a polyol, leading to the formation of urethane linkages.

To facilitate understanding of how polyurethane chemistry influences recyclability, Figure 1 schematically illustrates the polyaddition reaction between polyols and isocyanates leading to the formation of urethane linkages, which directly affect network architecture and resistance to depolymerization [9,10].

In recent years, innovative approaches such as bio-based polyols, non-isocyanate polyurethanes (NIPUs), hybrid chemical–biological recycling strategies, and selective catalytic processes have gained increasing attention. These developments reflect a broader shift from conventional waste treatment toward sustainable material cycles. Nevertheless, distinguishing truly innovative recycling concepts from established or incremental methods remains challenging and is rarely addressed systematically in the literature [11,12].

The aim of this review is therefore not only to summarize existing polyurethane recycling technologies but also to critically differentiate established industrial practices from emerging and potentially transformative approaches. Particular emphasis is placed on identifying the structural, chemical, and aging-related factors that govern recyclability, as well as on outlining key barriers and qualitative indicators of recycling efficiency, such as material recovery yield, preservation of functional properties, process energy demand, and scalability [13].

By integrating fundamental polyurethane chemistry with sustainability-oriented recycling perspectives, this review seeks to provide a coherent framework for understanding current limitations and future opportunities in polyurethane recycling. The analysis highlights how advances in chemistry, catalysis, and bio-based feedstocks may contribute to the transition toward more circular and resource-efficient polyurethane lifecycles [14].

## 2. Chemistry of Polyurethanes

This section summarizes key aspects of polyurethane chemistry that are essential for understanding recycling behavior and sustainability challenges. The chemical structure of polyurethanes—including the nature of polyols and isocyanates, crosslink density, phase separation, and the presence of additives—directly determines degradation pathways, aging mechanisms, and the response of materials to mechanical, chemical, and thermochemical recycling processes.

Fundamental chemical features such as urethane bond stability, intermolecular interactions, and network architecture strongly influence the feasibility and efficiency of different recycling routes. In particular, the resistance of crosslinked polyurethane systems to depolymerization, together with the chemical heterogeneity of commercial formulations, represents a major barrier to effective material recovery.

Accordingly, an understanding of polyurethane chemistry provides the necessary foundation for interpreting the recycling technologies discussed in subsequent sections. By linking molecular structure with aging-related degradation and end-of-life behavior, this section establishes a coherent framework for evaluating polyurethane recycling strategies from a sustainability-oriented perspective.

Polyurethane chemistry is based on a polyaddition reaction between isocyanates and hydroxyl-containing compounds, which provides the fundamental basis for the synthesis of polymer networks with broad industrial applicability [15,16,17]. The relatively simple reaction pathway, combined with the ability to tailor material properties over a wide range, led to the rapid adoption of polyurethanes as one of the most technologically important polymer families. In particular, the reaction between diisocyanates and polyester diols enabled the formation of linear or crosslinked polymer networks, laying the foundation for large-scale industrial production. Because the physicochemical properties of polyurethane materials are strongly dictated by the molecular structure of both the polyol and the isocyanate components, even small variations in their chemical composition or stoichiometric ratio can result in significant changes in macroscopic material behavior. As a result, structure–property relationships play a central role in the design and optimization of modern polyurethane systems [18].

Isocyanates employed in polyurethane synthesis may be aliphatic or aromatic and are frequently bifunctional; however, their effective functionality often exceeds three in practical formulations due to branching or prepolymer formation. For applications such as flexible foams, elastomers and thermoplastic polyurethanes, prepolymer isocyanates are commonly used. These species are obtained by partial reaction of NCO-functional aromatic or aliphatic isocyanates with selected polyols, allowing improved control over reactivity and molecular architecture. The broad availability of isocyanates and polyols with different molecular weights, functionalities and chemical structures enables precise modulation of polyurethane chemistry, facilitating the production of thermoplastic or thermosetting materials as well as flexible, semi-rigid or rigid systems through adjustment of the relative proportions of soft and hard segments within the polymer network [18].

Polyurethanes have been extensively investigated due to the versatility of their synthesis, which can often be carried out under mild conditions and allows straightforward chemical modification of the polymer backbone. Structural variation in either the polyol or the isocyanate component can be readily achieved using diverse synthetic strategies, providing access to materials with a wide spectrum of mechanical, thermal and functional properties. Flexible polyurethanes are typically produced using linear, high-molecular-weight polyols with low functionality, resulting in materials with high segmental mobility, whereas rigid polyurethanes are derived from low-molecular-weight, highly functional polyols that promote the formation of densely crosslinked networks. In addition, appropriate balancing of hydrophilic and hydrophobic segments enables the preparation of water-dispersible polyurethane systems, which are particularly relevant for coating and surface-protection applications [19].

### 2.1. Chemical Characteristics of Polyols

#### 2.1.1. Types of Polyols

To date, polyols constitute the most widely used group of starting materials for designing polyurethane materials with diverse properties. They can be divided into two major categories: high-molecular-weight and low-molecular-weight polyols. The molecular weight, functionality and chemical nature of polyols directly influence the final properties of polyurethane systems. High-molecular-weight polyols containing long alkyl segments are typically used for producing flexible or resilient polyurethanes due to their linear chains, which provide high segmental mobility, low functionality (2–3), and a correspondingly low degree of crosslinking. Polyether polyols of the polyoxyalkylene type are the most commonly employed polyols for flexible polyurethane foams. These materials are synthesized through the photopolymerization of oxiranes (epoxides) via an addition reaction initiated by a low-molecular-weight polyol [20].

This reaction can be catalyzed by three groups of compounds:

(1) **anionic catalysts**, such as NaOH, KOH, Ba(OH)_2_, Sr(OH)_2_, C_16_H_30_CaO_4_ or naphthenates;
(2) **cationic catalysts**, including BF_3_, CF_3_SO_3_H, PF_5_ or SbF_5_;
(3) **coordination catalysts**, such as Al(OR)_3_, Zn(OR)_2_, Ti(OR)_4_ or Zn_3_[Co(CN)_6_]_3_.

**Figure 2:**
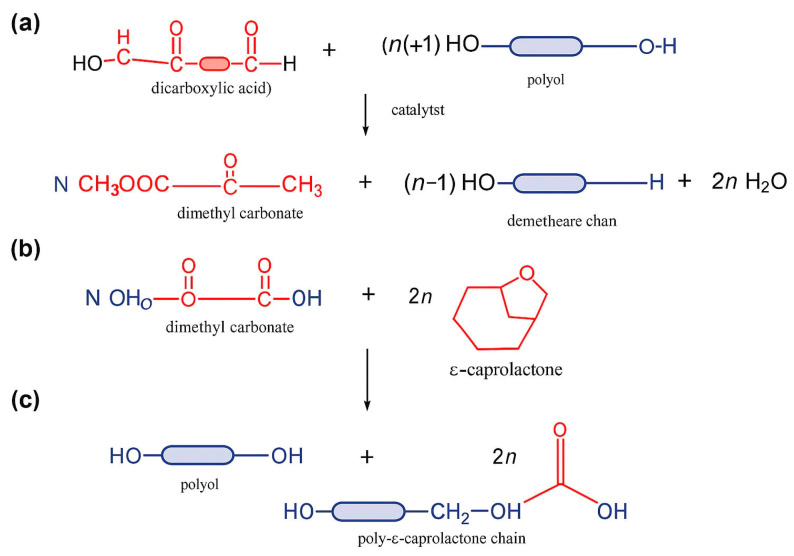
Reaction pathways commonly employed for the synthesis of polyester polyols: (**a**) polycondensation via esterification, (**b**) transesterification, and (**c**) ring-opening polymerization [18].

Polyester polyols represent the second most commonly produced polyol type used in polyurethane formulations. Polyester-based polyurethanes exhibit higher crystallinity, improved thermal stability and increased flame resistance compared with polyether-based systems, which is attributed to stronger intermolecular interactions along the polymer chains. However, polyester polyols are susceptible to hydrolytic degradation when exposed to moisture and heat over extended periods. They can be synthesized either through a polycondensation reaction between polyols and dicarboxylic acids, or by ring-opening polymerization of cyclic carbonates or lactones (Figure 2). Since water is a by-product in polycondensation, its continuous removal is required to shift the equilibrium toward polyester polyol formation. The reaction may proceed via self-catalysis due to the inherent acidity of carboxylic groups or may be catalyzed by C_7_H_8_O_3_S, C_16_H_30_O_4_Sn, CH_3_COO–Zn^+^ or Mn^2+^, which typically ensure higher reaction efficiency [21].

Figure 2 illustrates the main reaction pathways commonly employed for the synthesis of polyester polyols, including polycondensation via esterification, transesterification, and ring-opening polymerization.

In polyurethane technology, polyols used for flexible and rigid applications are generally divided into two main classes. The first class is characterized by relatively high hydroxyl values, typically in the range of 300–600 mg KOH g^−1^ and functionalities between 3 and 8. The second class exhibits lower hydroxyl values, usually between 30 and 50 mg KOH/g, and functionalities ranging from 2 to 3. Polyols from both classes can be obtained from renewable, bio-based feedstocks, providing opportunities for the development of more sustainable polyurethane systems [22].

#### 2.1.2. Bio-Based Polyols

Early attempts to introduce renewable components into polyurethane chemistry focused on the synthesis of polyether polyols using bio-based starters, such as sorbitol, sucrose and xylitol, during the polymerization of alkylene oxides, including ethylene oxide, propylene oxide and butylene oxide. In these systems, the bio-based content of the resulting polyols typically did not exceed 30%, which translated into a bio-carbon fraction of less than 8% in the final polyurethane foams. An alternative approach to the utilization of carbohydrates involves their fermentation to short-chain diols [23]. For instance, propylene glycol and butanediol can be produced from glucose-derived substrates using genetically engineered microorganisms. These diols are employed either in polymer systems other than polyurethanes, such as polyesters [23], or in polyurethane chemistry as chain extenders and as precursors for polyether polyols used in elastomeric materials, including poly(trimethylene ether) glycol and polytetrahydrofuran [24,25].

The most extensively investigated route for the production of bio-polyols is based on the use of vegetable oils as renewable raw materials; the resulting products are commonly referred to as natural-oil polyols (NOPs). Certain oils, such as castor oil and Lesquerella oil, can be applied directly as polyols owing to the presence of naturally occurring hydroxyl groups [26,27]. From an industrial perspective, castor oil is favored over Lesquerella oil because the latter exhibits a relatively low extraction yield of approximately 25 wt.%, which limits its commercial applicability. Castor oil is often chemically modified, for example by transesterification, to address limitations associated with its low hydroxyl value, reduced reactivity due to secondary hydroxyl groups and relatively low functionality. In general, the functionalization of vegetable oils to obtain suitable polyols relies on chemical transformations targeting ester functionalities or carbon–carbon double bonds present in fatty-acid chains [23].

Because most vegetable oils do not inherently contain hydroxyl groups, chemical introduction of hydroxyl functionalities is required to enable their use as polyols [28]. One widely applied method involves transesterification of vegetable oils, such as soybean, palm, linseed, sunflower and rapeseed oils, with multifunctional alcohols including glycerol, pentaerythritol, neopentyl glycol and trimethylolpropane. In this process, the ester groups of the triglycerides participate directly in the reaction. In a related approach, amidation of vegetable oils with amines, for example diethanolamine, yields fatty-acid diethanolamides that can serve as intermediates for polyurethane synthesis. These compounds improve polyol compatibility and facilitate the production of polyurethane materials with enhanced physico-mechanical properties [28,29].

#### 2.1.3. Natural-Oil Polyols (NOPs)

Natural-oil-based polyols may also be obtained through oxidative modification of carbon–carbon double bonds present in unsaturated fatty-acid chains. Reported oxidation routes include ozonolysis and hydroformylation, which generate aldehyde intermediates subsequently hydrogenated to primary hydroxyl groups, as well as oxidative formation of peroxides followed by reduction to the corresponding alcohols [30].

**Figure 3:**
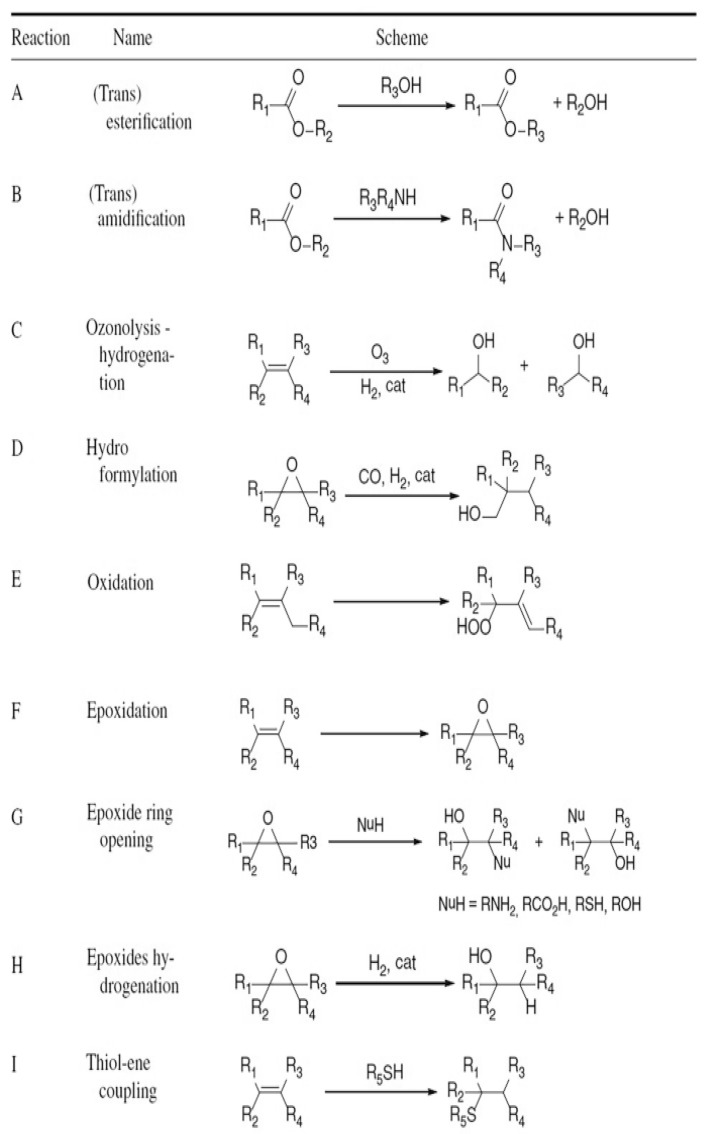
Principal reaction pathways employed for the functionalization of vegetable oils leading to the formation of natural-oil polyols (NOPs) [31].

Figure 3 summarizes the principal reaction pathways used for the functionalization of vegetable oils leading to the formation of natural-oil polyols (NOPs).

Commercial examples of natural-oil polyols include Renuva™ polyols produced by Dow Chemical (Midland, MI, USA), which are synthesized via hydroformylation. Among available functionalization routes, epoxidation is one of the most widely applied and industrially established methods for introducing hydroxyl functionalities at carbon–carbon double bonds within aliphatic fatty-acid chains. Compared with direct oxidation processes, epoxidation offers superior control over reaction selectivity and conversion, making it a robust and well-developed technology in industrial practice. Notably, one of the earliest large-scale commercial bio-polyols, BiOH^®^ produced by Cargill, (Minneapolis, MN, USA). was obtained through epoxidation of C=C bonds followed by methanolysis carried out under ambient pressure and moderate temperature conditions [31,32,33].

More recently, the thiol–ene reaction, despite having been known since the early twentieth century, has gained renewed attention as an efficient alternative route for bio-polyol synthesis. This approach is characterized by simple reaction conditions, solvent-free operation, the absence of high-temperature requirements and high conversion efficiency. Owing to these advantages, thiol–ene chemistry is widely regarded as a green and cost-effective functionalization strategy with growing relevance in sustainable polyurethane chemistry [34,35,36].

The thiol–ene methodology has been successfully applied to the preparation of natural-oil-based polyols derived from corn, soybean and castor oils. In this process, unsaturated carbon–carbon bonds present in fatty-acid chains are functionalized using mercapto compounds, resulting in polyols containing primary hydroxyl groups located at the termini of the modified chains [33,34,35,36,37].

The physicochemical and application-related properties of natural-oil-based polyols are strongly dependent on the selected functionalization pathway. A comparative overview of key characteristics and practical considerations associated with polyols derived from different biomass sources and modification strategies is provided in Table 1.

Prociak et al. [38] synthesized polyols from rapeseed oil using epoxidation, transamidation and transesterification, and systematically evaluated their reactivity during polyurethane foam formation. Their results demonstrated that natural-oil polyols obtained via transamidation and transesterification exhibit significantly higher reactivity than those produced through epoxidation. This behavior was attributed to the presence of amine functionalities, which act as intrinsic catalysts during polyurethane formation. As a result, highly reactive polyols are particularly suitable for polyurethane systems requiring rapid reaction kinetics, such as spray-foam applications, and their incorporation leads to improved thermal performance of the resulting PU foams.

In contrast, polyols derived from epoxidation display lower reactivity and are therefore better suited for thermal-insulation foams, where they promote the formation of a more uniform cellular structure, contributing simultaneously to enhanced mechanical properties. A comparative assessment of functionalization strategies and the resulting polyol characteristics was reported by Amri et al. [39]. Their study showed that bio-polyols obtained via ozonolysis followed by reduction are more reactive than those produced by epoxidation and subsequent ring-opening reactions. This difference arises from the presence of primary hydroxyl groups in ozonolysis-derived polyols, whereas epoxidation-based routes predominantly generate secondary hydroxyl functionalities.

Nevertheless, the functionality of ozonolysis-derived polyols is typically limited to three hydroxyl groups per triglyceride molecule. In contrast, epoxidized polyols offer greater flexibility, as their functionality can be precisely adjusted by controlling the epoxide content, which depends on the type of vegetable oil and the amount of ring-opening agent employed. Hydroformylation provides an additional route to polyols containing primary hydroxyl groups with tunable functionality. Thiol–ene chemistry offers comparable advantages; however, in systems based on highly unsaturated fatty acids, competing oligomerization reactions may occur, making careful selection of the vegetable-oil feedstock essential [31,32,33].

Despite the numerous advantages associated with vegetable-oil-derived polyols, including the renewability of the feedstock, several limitations remain. Certain synthetic pathways require expensive catalysts, rendering bio-polyols more costly than their petrochemical counterparts. In addition, high viscosity, residual impurities and unreacted fatty-acid fragments can restrict their direct substitution for conventional petroleum-based polyols in polyurethane formulations [33,34].

Consequently, although a wide range of natural-oil polyols is commercially available, these materials are most often employed in combination with petrochemical polyols rather than as complete replacements. It should also be noted that many vegetable oils used for bio-polyol production are edible, which introduces competition between material applications and food supply [40]. For this reason, recent research efforts have increasingly focused on second-generation biomass resources, which are non-edible and therefore do not compete with food markets. Non-edible vegetable oils constitute an important class of such feedstocks, as they are inexpensive, can be produced globally—including in arid regions and on marginal land—and are well suited for chemical modification [41].

In general, the chemical pathways applied to non-edible vegetable oils are analogous to those used for edible oils and rely on similar functionalization strategies. Representative examples include tung oil, jatropha oil, jojoba oil, linseed oil and castor oil; oils derived from algae are also classified as non-edible feedstocks. Jatropha oil, for instance, contains a high proportion of unsaturated fatty acids, primarily oleic (43%) and linoleic (34%) acids, which is advantageous for chemical modification and for the synthesis of value-added chemicals and polymers [42].

Algae-derived oils represent another promising renewable feedstock for polyol synthesis. Their main limitation lies in the presence of organic impurities, such as hydrophobic cofactors and pigments, which may interfere with chemical processing [43,44]. Compared with conventional vegetable oils, algae oils typically exhibit a higher degree of unsaturation, although this strongly depends on the algal species [45]. Petrovic et al. [45] reported that the epoxidation of algae oil proceeds with lower conversion than that of vegetable oils and requires higher catalyst concentrations. In the same study, ozonolysis was also investigated and yielded polyols with low functionality (approximately 1.5), which limited their applicability in polyurethane formulations. In contrast, when ozonolysis was combined with subsequent oxidation using sodium chlorite, azelaic acid could be obtained from palmitoleic acid extracted from algae oil. This intermediate was further reacted with ethylene glycol via acid-catalyzed polycondensation to produce a polyester polyol suitable for the manufacture of shape-memory polyurethane foams [44].

An alternative strategy to avoid competition with food resources involves the utilization of used cooking oils (UCO) as renewable feedstocks. On a global scale, more than 27 million tons of UCO are generated annually, making them both abundant and economically attractive, with prices typically two to three times lower than those of virgin vegetable oils [46]. Conversion of UCO into bio-based chemicals, particularly bio-polyols, offers both economic and environmental benefits by reducing reliance on virgin resources and supporting circular-economy concepts. During thermal treatment associated with frying, UCO undergo several chemical transformations, including:

(1) hydrolysis, which increases the concentration of polar species such as glycerol and free fatty acids and leads to darkening;
(2) oxidation, which alters the content of conjugated dienes and trienes;
(3) polymerization of fatty-acid double bonds, resulting in the formation of triglyceride dimers and higher oligomers [24,44,46].

### 2.2. Characteristics of Isocyanates

Isocyanates are defined by the presence of the highly reactive isocyanate functional group (–N=C=O), which plays a central role in polyurethane formation [47,48]. This functional group readily reacts with hydroxyl groups, directly participating in the polyaddition reaction that leads to the formation of urethane linkages. Compared with polyols, the range of commercially available isocyanates is relatively limited. Nevertheless, large-scale industrial production is dominated by a small number of major manufacturers, including Dow, Huntsman, Bayer, BASF, Shell, ICI and AC [49].

The technological importance of isocyanates is primarily associated with their high reactivity toward hydroxyl-containing compounds, which enables efficient and controllable polyurethane synthesis. A common industrial route for isocyanate production involves amine-based precursors, which are converted into the corresponding isocyanates through reaction with phosgene [50] according to the general reaction scheme:

(1) $$R-NH_{2}\;+\;{COCl}_{2}\to \;R-N=C=O\;+\;2HCl$$

Two main classes of isocyanates are employed in industrial polyurethane production: aromatic and aliphatic isocyanates. Aromatic isocyanates introduce rigid segments into the polyurethane backbone and exert a strong influence on the final material properties. Representative examples include toluene diisocyanate (TDI) and methylene diphenyl diisocyanate (MDI), both of which contain aromatic rings and are predominantly used in rigid and thermosetting polyurethane systems. Owing to their relatively low cost and high intrinsic reactivity compared with other diisocyanates, aromatic isocyanates are widely preferred in large-scale industrial applications [47,49]. A major limitation of this class is their limited resistance to ultraviolet radiation; prolonged UV exposure typically leads to yellowing or darkening, which restricts their use primarily to indoor applications.

Aliphatic isocyanates, including hexamethylene diisocyanate (HMDI) and isophorone diisocyanate (IPDI), are commonly applied in coating formulations. These compounds exhibit good compatibility with pigments, maintain surface gloss and provide excellent resistance to UV radiation [51]. In general, polyurethanes terminated with isocyanate groups are characterized by hydrophobicity, chemical stability and increased rigidity, whereas systems terminated with hydroxyl groups tend to be more hydrophilic, exhibit greater flexibility and display higher sensitivity to environmental factors [52].

**Figure 4:**
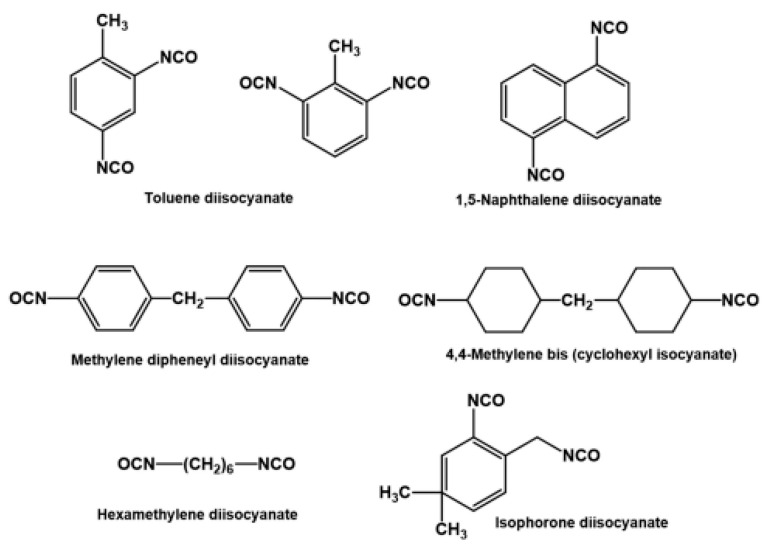
Isocyanates used for polyurethane synthesis [53].

Figure 4 provides an overview of the main isocyanates commonly used for polyurethane synthesis.

One of the key concerns related to isocyanates is their inherent toxicity. Under processing conditions, particularly at elevated temperatures, isocyanates may generate hazardous species, including volatile monomeric diisocyanates and aromatic amines formed as a result of partial degradation of urethane linkages These compounds are recognized as potentially harmful to human health [49,54].

One approach to reducing these risks is the use of polymeric isocyanates, which exhibit significantly lower vapor pressure compared with monomeric isocyanates, thereby improving process safety and reducing worker exposure [54]. In parallel, there is growing interest in isocyanate-free routes for polyurethane synthesis. These emerging technologies rely on alternative chemistries, such as cyclic carbonates reacting with amines to form so-called NIPUs (Non-Isocyanate Polyurethanes) [55,56].

#### 2.2.1. Non-Isocyanate Polyurethanes (NIPUs)

An illustrative example of an isocyanate-free strategy is the synthesis of polyurethanes derived from limonene. In this approach, the carbon–carbon double bond present in limonene is first converted into an epoxide. The epoxidised intermediate subsequently reacts with carbon dioxide under elevated pressure in the presence of a suitable catalyst, leading to the formation of cyclic carbonate groups. In the final step, aminolysis of the cyclic carbonates occurs, resulting in the formation of urethane linkages and yielding non-isocyanate polyurethanes (NIPUs) structurally based on limonene [57].

**Figure 5:**
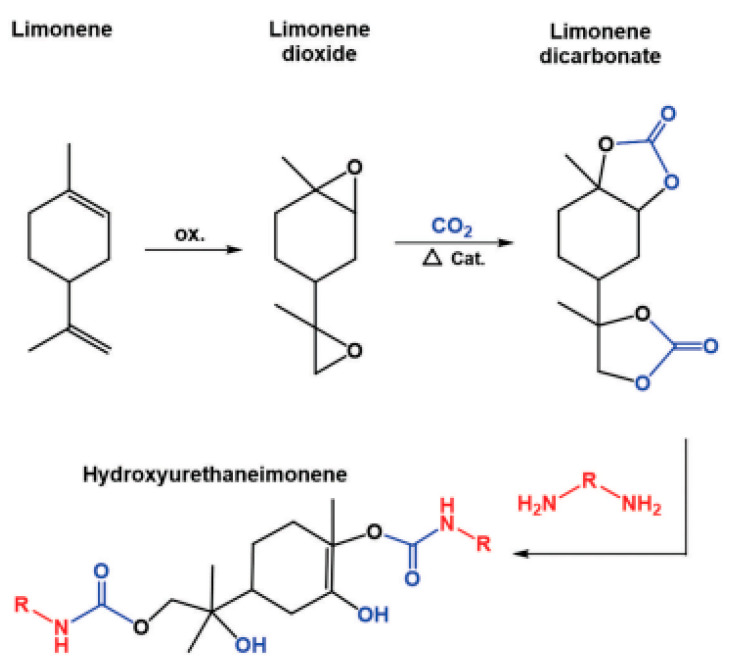
Isocyanate-free polyurethane synthesis via epoxidation, CO_2_ incorporation forming cyclic carbonates, followed by aminolysis [58].

Figure 5 schematically presents an isocyanate-free polyurethane synthesis route involving epoxidation, carbon dioxide incorporation to form cyclic carbonates, and subsequent aminolysis.

Patent analysis clearly indicates increasing interest in polyurethane technologies based on renewable raw materials, and a review of scientific publications confirms the growing body of research dedicated to bio-isocyanates and bio-based polyurethanes. These studies may play an important role in shaping future regulatory frameworks and industrial practices by providing key insights into the risks associated with isocyanate use. Patents also serve as an essential source of information on technological trends and can reveal research gaps between academia and industry [58].

Several representative patent applications addressing the synthesis of bio-isocyanates are summarized below. Patents US9950996B2 (Parakash Purushottam Wadgaonkar; Sachin Suresh Kuhire. Bio-based aromatic isocyantes for preparation of polyurethanes. 25 April 2018), US4749806A (Igor Tkatchenko; Rabih Jaouhari; Michel Bonnet; Gordon Dawkins; Serge Lecolier. Process for the synthesis of isocyanates and of isocyanate derivatieves. 7 June 1988) and CN102659631B (Wu Shuyonga. One-step synthesis of ethyl isocyanate. 7 May 2014)disclose phosgene-free routes for isocyanate production, thereby substantially reducing both the environmental burden and the inherent safety risks associated with conventional phosgenation-based technologies [57]. Patent WO2014147142A1 (Rolf Klucker; Jean-Marie Bernard. Allophanate composition. 25 Spetember 2014) discloses an allophanate-type bio-isocyanate intended for use in polyurethane coating formulations [59].

Another relevant patent application is EP3819259A1 (Covestro AG. Verfahren zur isocyanat- und polyurethan-herstellung mit verbesserter nachhaltigkeit. 6 November 2019), which describes a low-emission route for isocyanate synthesis. In this process, carbon monoxide is generated via the reverse water–gas shift (RWGS) reaction, while hydrogen obtained from water electrolysis is used for chlorine production. Oxygen produced during electrolysis is utilized to support the combustion of polyurethane waste. The carbon monoxide formed subsequently reacts with chlorine to generate phosgene, which is then converted into isocyanates through reaction with amines. Although phosgene remains an intermediate in this process, the overall approach significantly reduces carbon dioxide emissions derived from fossil resources and is consistent with the principles of more sustainable polyurethane manufacturing [59].

A further example is CN113461894A, describing a polyurethane foam produced from a bio-based isocyanate, illustrating the increasing use of bio-derived feedstocks in polyurethane synthesis [60].

Bio-based TDI can also be produced using toluene obtained from biomass, which is subsequently subjected to nitration, hydrogenation, carbonylation and oxidation. These examples represent only a fraction of the current knowledge landscape in the field of sustainable use of isocyanates. Nevertheless, patent analysis clearly shows a growing correlation between scientific research trends and patent activity related to bio-isocyanate technologies [58].

An overview of the data discussed in this section is presented in Table 2.

#### 2.2.2. Bio-Based Isocyanates: Sources and Industrial Availability

Bio-based isocyanates are already available on the commercial market; however, their overall availability remains limited. One example is the aliphatic diisocyanate Tolonate™ X FLO 100 introduced by Vencorex Chemicals (Saint-Priest, France). This material is derived from palm oil, exhibits low viscosity and contains approximately 32% bio-based carbon [61]. Tolonate™ X FLO 100 is primarily applied as a precursor for epoxy resins used in coating formulations. To the best of current knowledge, no reports have been published on the production of flexible polyurethane foams using this isocyanate.

Covestro (Leverkusen, Germany) has also introduced a bio-based polyisocyanate in the form of a PDI trimer marketed as Desmodur^®^ eco N 7300. This product represents the first bio-based curing agent designed for lightweight polyurethane coating systems. It contains approximately 70% renewable raw materials and demonstrates a carbon footprint that is about 30% lower than that of petrochemical analogues. Desmodur^®^ eco N 7300 is used in coating applications for the automotive and plastics sectors and is compatible with both solvent-borne and solvent-free systems. In terms of performance, it is reported to be comparable to conventional petrochemical-based curing agents [62].

Another commercially available bio-isocyanate is STABIO™ PDI, produced by Mitsui Chemicals (Tokyo, Japan). This material is an aliphatic polyisocyanurate derived from bio-based 1,5-pentamethylene diisocyanate and contains more than 60% biomass-derived carbon. It is characterized by a high NCO content, good solvent resistance and excellent weathering stability, which makes it suitable for use in paint and coating formulations [63].

Detailed compositional information on these commercial bio-isocyanates is generally not disclosed in patent literature, likely due to the use of trade-secret protection strategies reflecting the proprietary nature of the technologies involved. A comparison of the most relevant commercially available bio-isocyanates is provided in Table 3.

**Figure 6:**
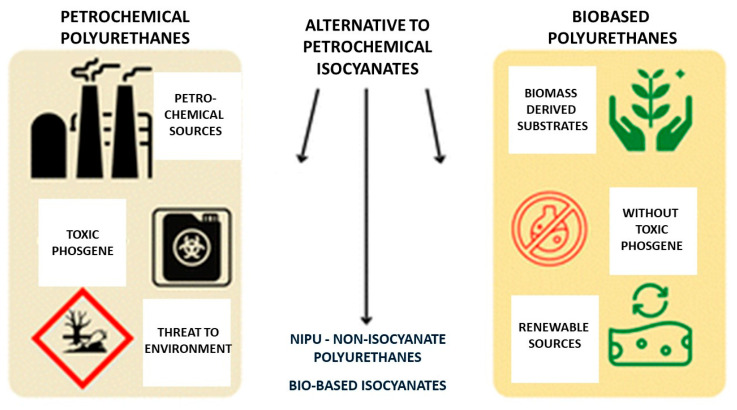
Graphical classification of isocyanates according to their origin.

**Figure 7:**
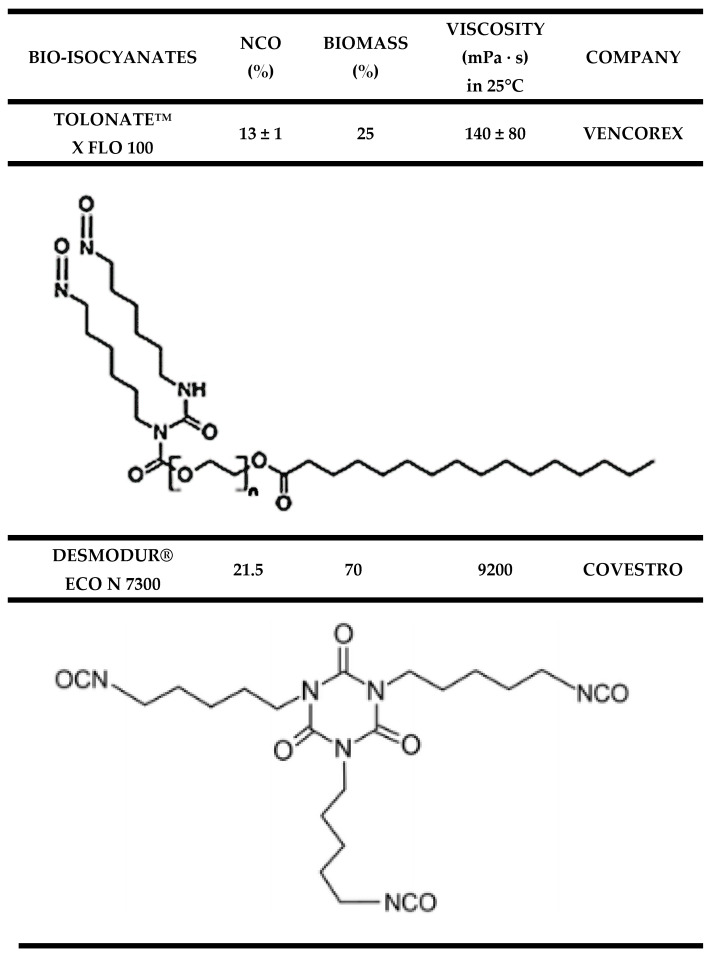
Comparison of bio-isocyanates [64,65,66].

Figure 6 and Figure 7 graphically classifie isocyanates according to their origin, distinguishing between fossil-based and bio-based feedstocks.

A summary of the key properties and characteristics discussed in this section is provided in Table 3.

Bio-isocyanates can be obtained via classical rearrangement reactions, including the Curtius, Hofmann and Lossen rearrangements, as schematically shown in Figure 6. These synthetic routes enable the formation of isocyanates without the use of phosgene; however, they are associated with significant practical limitations. In particular, the Curtius rearrangement requires acyl azides as key intermediates, which are highly toxic and potentially explosive, posing substantial safety and technological challenges for large-scale implementation. In contrast, the Hofmann and Lossen rearrangements are restricted to the synthesis of aliphatic isocyanates, which limits their applicability in designing more structurally diverse polyurethane systems [64,65].

**Figure 8:**
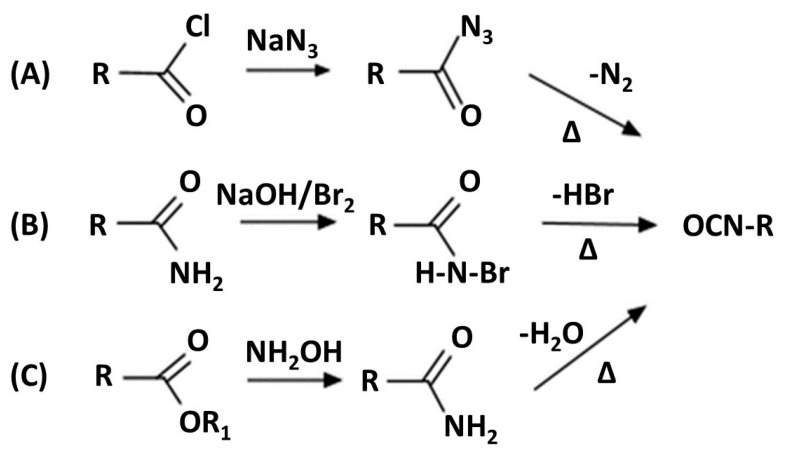
Isocyanate synthesis via classical rearrangement reactions: (**A**) Curtius, (**B**) Hofmann and (**C**) Lossen rearrangements [67].

Figure 8 illustrates classical rearrangement reactions used for isocyanate synthesis, including the Curtius, Hofmann, and Lossen rearrangements.

In addition to established synthetic routes, isocyanates can also be derived from a variety of biomass-based precursors. The synthesis of fatty-acid-derived diisocyanates has been reported since the second half of the twentieth century. Isocyanates obtained from vegetable oils generally exhibit aliphatic structures, which results in lower reactivity compared with aromatic isocyanates of petrochemical origin [68].

Changqing Fu et al. [67] reported that the synthesis of a diisocyanate derived from undecylenic acid obtained from castor oil. The synthetic route involved thiol–ene coupling followed by a Curtius rearrangement, yielding the target diisocyanate with a conversion of 76.4%. This compound was subsequently employed as a precursor for the preparation of a bio-based waterborne polyurethane dispersion (BPUD) through reaction with castor oil and a hydrophilic carboxylic-acid-type chain extender, which was also synthesized from castor oil using thiol–ene chemistry. The resulting polyurethane materials exhibited high flexibility and low elongation at break. A major limitation of this approach, however, is the requirement for sodium azide as a reagent, which is highly explosive and poses significant challenges for safe handling and process scalability.

Hojabri et al. [69] reported an alternative route for the synthesis of aliphatic 1,7-heptamethylene diisocyanate (HPMDI) starting from oleic acid via a Curtius rearrangement (Figure 8). The process involved thermal decomposition of the corresponding acyl azide intermediate, which subsequently rearranged to form the target isocyanate. The obtained HPMDI was subsequently used for polyurethane synthesis, yielding materials with physicochemical properties comparable to those of analogous polymers derived from petrochemical feedstocks.

Hydrolysis of both the acyl azide intermediate and the resulting isocyanate was prevented by conducting the reaction under strictly anhydrous conditions. Nevertheless, the process exhibited notable drawbacks, including the requirement for strictly moisture-free conditions and the use of hazardous azide intermediates that necessitate low-temperature handling. In this process, tetrahydrofuran (THF) is required as a reaction solvent to ensure proper dissolution of the reactants and to facilitate efficient reaction progress. For these reasons, industrial-scale implementation of the process would require further optimization and the development of milder reaction conditions.

In a related study, Hojabri et al. [69] reported the synthesis of poly(ester-urethanes) based on polyester diols and aliphatic diisocyanates derived from renewable feedstocks. Structural analysis confirmed the expected chemical architecture of the resulting polymers, and their properties were shown to be comparable to those of polyurethane materials prepared from petrochemical precursors.

**Figure 9:**
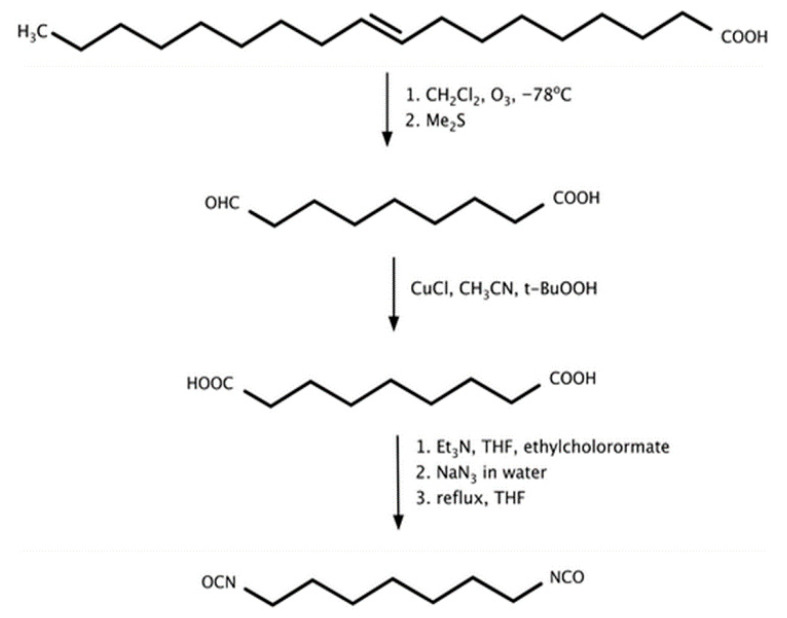
Synthesis of 1,7-heptamethylene diisocyanate (HPMDI) from oleic acid via the Curtius rearrangement [66,69].

Figure 9 depicts the synthesis pathway of 1,7-heptamethylene diisocyanate (HPMDI) from oleic acid via the Curtius rearrangement.

Hojabri et al. [69] also reported the synthesis of 1,16-diisocyanatohexadec-8-ene (HDEDI) from oleic acid using a Curtius rearrangement (Figure 9). In the initial step, oleic acid was subjected to olefin metathesis catalyzed by a Grubbs catalyst, yielding an unsaturated dicarboxylic acid intermediate. This compound was subsequently converted via a Curtius rearrangement employing ethyl chloroformate, triethylamine and anhydrous tetrahydrofuran as the reaction medium. A solution of sodium azide was then added dropwise under an inert nitrogen atmosphere, leading to the formation of HDEDI as a light-yellow oil. The resulting bio-derived diisocyanate was subsequently utilized for the synthesis of fully bio-based polyurethane materials.

The results indicated that polyurethanes synthesized from HDEDI exhibited stronger hydrogen-bonding interactions than those prepared from HPMDI, which translated into a higher tensile strength at break. At the same time, these materials exhibited a lower Young’s modulus and increased elongation at break.

A notable limitation was the relatively low melting temperature of polyurethanes based on HDEDI (80 °C) and HPMDI (73 °C), which was significantly lower than that observed for polyurethanes synthesized from HDI. Another limitation of the process was the requirement for large amounts of solvents during the step in which the diol was separated from the reaction mixture, significantly hindering process scalability.

The principal factor limiting the commercial viability of both isocyanates (HPMDI and HDEDI) is their relatively low reaction yield, which has so far been achieved only at laboratory scale, amounting to 68% and 57%, respectively.

**Figure 10:**
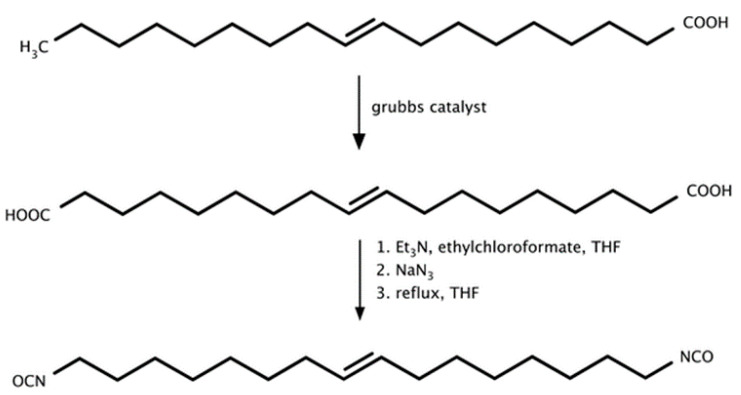
Synthesis of unsaturated diisocyanates from oleic acid [66,70].

Cifarelli et al. [70] reported that bio-polyols from epoxidised soybean oil and linseed oil using a solvent-free approach based on reaction with caprylic acid or 3-phenylbutyric acid in the presence of triethylamine as a catalyst. As shown in Figure 10, the obtained biopolyols were subsequently used for the preparation of polyurethane foams in combination with the partially bio-based isocyanate Tolonate™ X FLO 100, with water employed as the blowing agent.

The resulting green polyurethane (G-PU) foams exhibited an open-cell morphology characterized by a well-developed and interconnected pore network.

The mechanical properties of the bio-based PU foams were characterized by a slightly lower Young’s modulus and reduced compressive resilience compared with the reference material. Unfortunately, polyols derived from vegetable oils tend to exhibit higher flammability than their petrochemical counterparts [71,72]. The produced foams may be unsuitable for commercial applications that require high fire resistance. Although several strategies exist for improving flame retardancy—such as incorporating phosphorus- or nitrogen-containing functional groups—these modifications significantly complicate the synthesis and increase production costs.

Unsaturated triglycerides of vegetable oils bearing isocyanate functionalities have also been reported as precursors for polyurethane synthesis [73,74]. The preparation of these materials involved a multistep synthetic sequence. In the initial step, methyl oleate was selectively brominated at the allylic position using N-bromosuccinimide, providing a functionalised intermediate suitable for further transformation. The crude product was purified by silica gel column chromatography using n-pentane/CHCl_3_ as the eluent.

In the next step, the purified intermediate was dissolved in THF, followed by the gradual addition of AgNCO_3_, with the reaction carried out under light-protected conditions. The resulting methyl oleate isocyanate was then mixed with methanol and heated under reflux overnight under strictly anhydrous conditions. The final allyl isocyanates were obtained with yields in the range of 60–70%.

Substitution of methyl oleate with dried soybean oil did not hinder the subsequent reaction, as the resulting alkyl halides readily underwent conversion in the presence of AgNCO_3_.The obtained polyurethanes and polyureas exhibited low mechanical strength, high elongation, and a high swelling index. These materials may be suitable for applications in which high mechanical performance is not a critical requirement.

The main limitation of this synthetic route is the need for very expensive AgNCO_3_, which cannot be replaced by cheaper reagents. Furthermore, the process requires solvents such as THF, reducing its environmental benefits.

**Figure 11:**
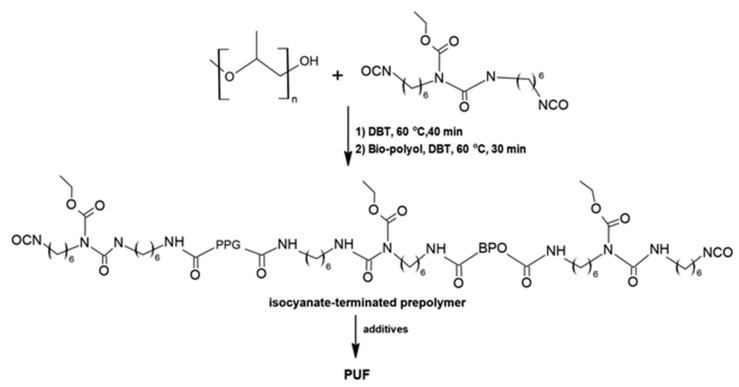
Schematic representation of polyurethane foam synthesis using a bio-polyol and a partially bio-based isocyanate [66,73].

Oleic and erucic acid esters were likewise employed as precursors for diisocyanate synthesis. Following nucleophilic substitution, the linear isocyanates 1,19-diisocyananonadecane and 1,23-diisocyananotricosane were obtained [75]. The synthetic route comprised four consecutive steps:

1. Dimethyl nonadecanedioate and dimethyl tricosanedioate were synthesized via somerizing alkoxycarbonylation (Figure 11).
2. These products were reduced to the corresponding diols.
3. The diols were converted to bromides via an Appel II reaction. Finally, nucleophilic substitution yielded the target diisocyanates.
4. The overall process yield reached up to 40%.

Despite the moderate yield, the method preserves the hydrocarbon chain length, which is a significant advantage. The resulting diisocyanates were subsequently applied in polyaddition reactions with diols to obtain transparent bio-based polyurethane–polyether copolymers. Polymers obtained via this route exhibited lower melting temperatures, reduced ductility, and lower elasticity. To enable industrial-scale implementation, improvements in yield and the elimination of toxic solvents will be essential.

**Figure 12:**
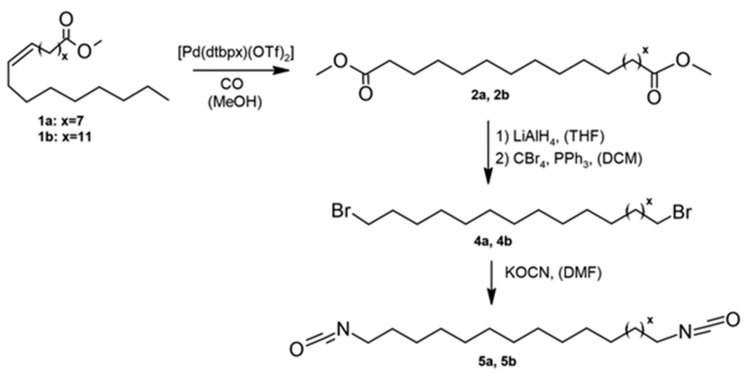
Synthesis of 1,19-diisocyananonadecane and 1,23-diisocyananotricosane from fatty-acid-derived precursors [76].

Bhutra et al. [76] reported an environmentally benign, phosgene-free route for isocyanate synthesis, as illustrated in Figure 12. In this approach, castor-oil-derived sebacic acid and undecylenic acid were employed as starting substrates. Both acids were first reacted with hydrazine hydrate, leading to the formation of a solid intermediate, which was isolated by filtration and subsequently heated under reflux in ethanol. After a second filtration step, a white crystalline material was obtained.

The resulting suspension was then treated with concentrated hydrochloric acid, acetic acid and dichloromethane, followed by dropwise addition of an aqueous sodium nitrite solution. The reaction mixture was subsequently heated under reflux in anhydrous tetrahydrofuran under a nitrogen atmosphere. Using this multistep protocol, the target isocyanate products were obtained with an approximate yield of 70%.

The synthesized diisocyanate (DITD) contained non-equivalent NCO groups and exhibited instability, whereas the isocyanate with equivalent structural units was stable and easy to process. These diisocyanates were subsequently reacted with various diols to produce polyurethane materials whose properties depended on the selected monomer combinations, resulting in both amorphous and semi-crystalline polymer structures. Polyurethanes synthesized from DITD and propanediol displayed an amorphous structure, whereas an increase in the carbon-chain length of the diol resulted in the formation of semi-crystalline materials. In contrast, polyurethanes prepared from DIO in combination with linear aliphatic diols were fully semi-crystalline.

The melting temperature increased with an increase in the number of methylene units from three to four; however, further extension of the aliphatic segments resulted in a decrease in melting temperature due to increased chain flexibility.

**Figure 13:**
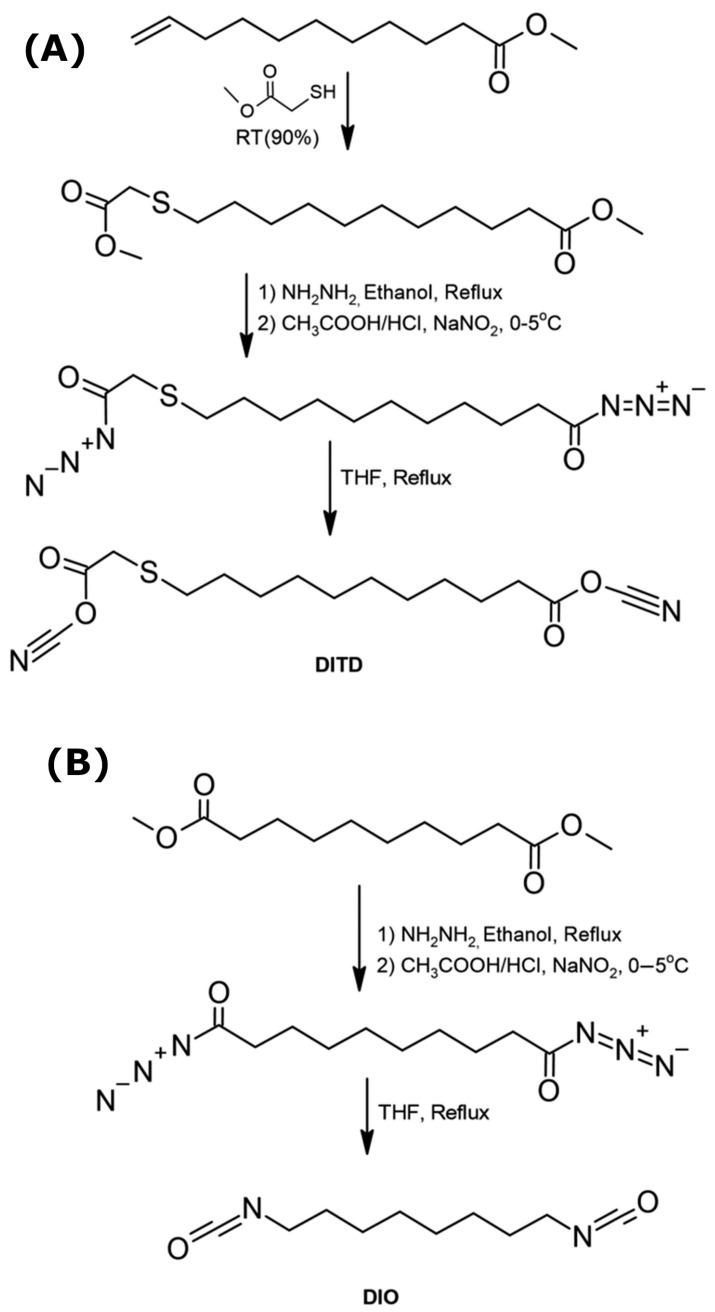
Comparison of two synthetic routes for the preparation of diisocyanates: (**A**) Method A based on a sulfur-containing intermediate and (**B**) Method B involving a sulfur-free pathway [66,76].

The synthesis pathway (Method A and B) of diisocyanates derived from castor-oil-based substrates is schematically illustrated in Figure 13.

#### 2.2.3. Lignin as a Renewable Source for Isocyanate Production

Lignin represents an abundant renewable raw material and is therefore considered an attractive feedstock for the development of bio-based chemicals [77]. Structurally, lignin is an amorphous, highly heterogeneous polymer formed through the dehydrogenative polymerisation of hydroxycinnamyl alcohols [78]. Its macromolecular architecture is composed of three principal phenylpropane units—p-hydroxyphenyl, guaiacyl and syringyl—interconnected through a variety of carbon–carbon and ether linkages. Lignin is isolated from lignocellulosic biomass using different industrial processes, and the extraction method strongly influences its molecular structure, functional-group distribution and reactivity [79].

As a major by-product of the pulp and paper industry, lignin represents an underutilized resource, making its valorisation particularly attractive. In 2010, global lignin production was estimated at approximately 50 million tonnes, of which only about 2% was utilized in applications such as dispersants, adhesives and surfactants [76]. In recent years, increasing attention has been directed toward lignin-derived aromatic building blocks—including vanillin, syringic acid, vanillic acid, guaiacol and syringol—as renewable precursors for polyurethane synthesis [80].

While lignin is most commonly explored as a source of renewable polyols, several studies have also investigated its conversion into isocyanates. The earliest reports on lignin-derived isocyanates date back to 1981. In the publication Lignin-Derived Polyols, Polyisocyanates, and Polyurethanes, two distinct approaches for transforming lignin-derived carboxylic acids into isocyanates were described, demonstrating the feasibility of using lignin as a precursor for aromatic isocyanate synthesis [81].

In Method A (Figure 13), the carboxylic acid is first converted into the corresponding acyl chloride by reaction with thionyl chloride, followed by transformation into an acyl azide using sodium azide under either aqueous or non-aqueous conditions. The resulting acyl azide undergoes a Curtius rearrangement, yielding the corresponding isocyanate.

**Figure 14:**
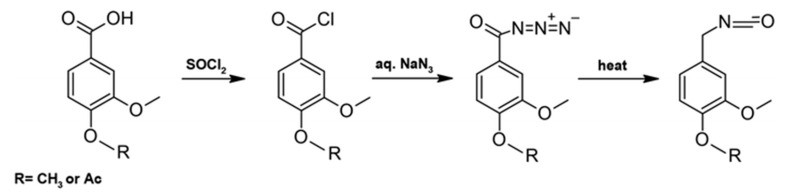
Reaction pathway for the conversion of carboxylic acids into isocyanates via rearrangement reactions [66,82,83].

The reaction pathway for the conversion of carboxylic acids into isocyanates via rearrangement reactions is schematically illustrated in Figure 14.

Kuhire et al. [82,83] reported the synthesis of aromatic diisocyanates, namely bis(4-isocyanato-2-methoxyphenoxy)alkane and bis(4-isocyanato-2,6-dimethoxyphenoxy)alkane, using a Curtius rearrangement strategy (Figure 14). The resulting aromatic diisocyanates were subsequently utilized for the preparation of poly(ether urethanes) through reaction with bio-based aliphatic diols, specifically 1,10-decanediol and 1,12-dodecanediol, which served as chain extenders. This strategy enabled the synthesis of polyurethane materials incorporating both aromatic lignin-derived segments and renewable aliphatic soft segments within the polymer backbone.

**Figure 15:**
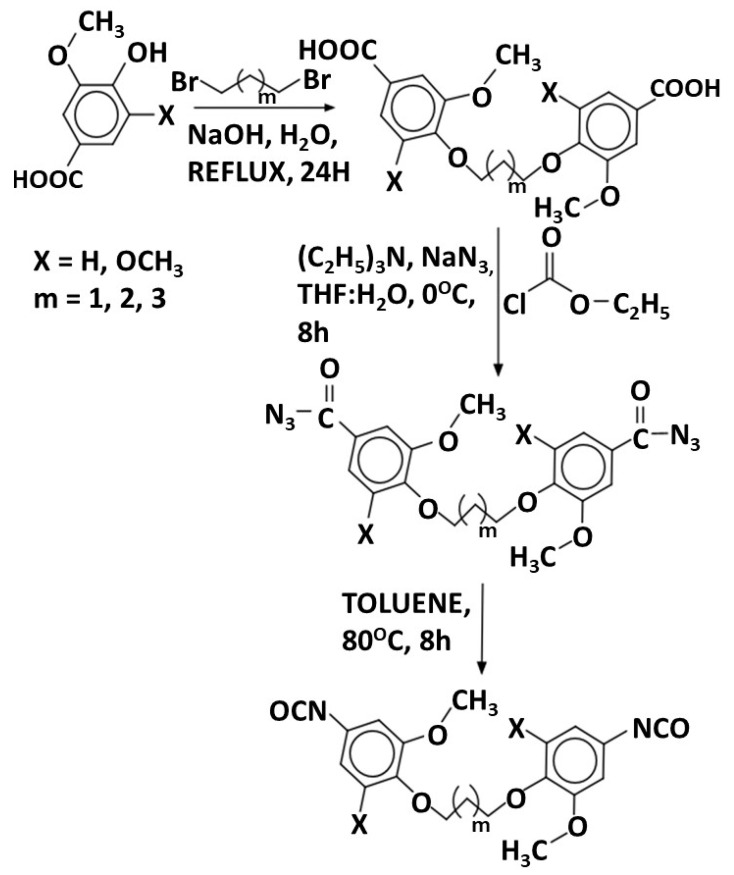
Synthesis of aromatic diisocyanates via lignin-derived intermediates [66].

**Figure 16:**
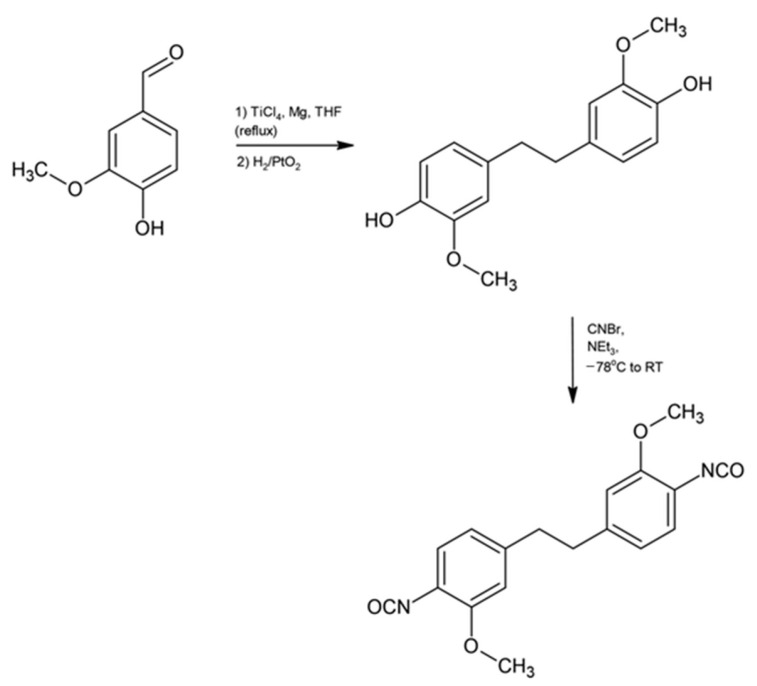
Synthesis of vanillin-derived bisphenols and cyanate esters [66].

A direct synthetic route based on McMurry coupling has also been reported for the preparation of aromatic isocyanate-related compounds. In this approach, vanillin was reacted with a TiCl_4_/Mg system in tetrahydrofuran, yielding a stilbene intermediate (Figure 15 and Figure 16).

The resulting stilbene subsequently underwent reaction with cyanogen bromide (CNBr) in the presence of triethylamine (NEt_3_), leading to the formation of cyanate esters. The products were isolated by filtration followed by washing with water and were obtained in satisfactory yields. Structural confirmation was achieved using single-crystal X-ray diffraction analysis [66]. The resulting bis(cyanate ester) compounds were further subjected to thermal curing under mild conditions, producing crosslinked thermoset materials with properties comparable to those of conventional petroleum-derived epoxy resins.

Lignin is characterized by high availability, low cost and biodegradability, which has driven growing interest in its use as a renewable component of polymer matrices and other industrial materials [84]. However, lignin exhibits pronounced structural and functional variability, which strongly depends on its botanical origin as well as the extraction and processing methods employed. These factors lead to significant heterogeneity in molecular structure and functional-group distribution, which can adversely affect the consistency and performance of the resulting materials. Such variability represents a major limitation for commercial applications, where high material purity and reproducible properties are essential to ensure reliable processing and end-use performance [85].

#### 2.2.4. Amino-Acid-Based Isocyanates

Isocyanates derived from amino acids are predominantly explored for applications in biomedical polyurethane materials. The study entitled Green Polyurethanes from Renewable Isocyanates and Bio-Based White Dextrans reports synthetic strategies for the preparation of polyurethane films using bio-derived isocyanates. In this work, ethyl L-lysine diisocyanate (LLDI), ethyl L-lysine triisocyanate (LLTI) and the commercial isophorone diisocyanate (IPDI) were employed as reactive components. Polyurethanes synthesized from IPDI exhibited transparent and rigid characteristics, whereas materials obtained from LLDI were flexible and displayed an opaque, milky-white appearance. However, polyurethane materials based on LLDI showed an increased tendency toward cracking compared with their IPDI-based analogues, indicating limitations in their mechanical durability [86].

**Figure 17:**
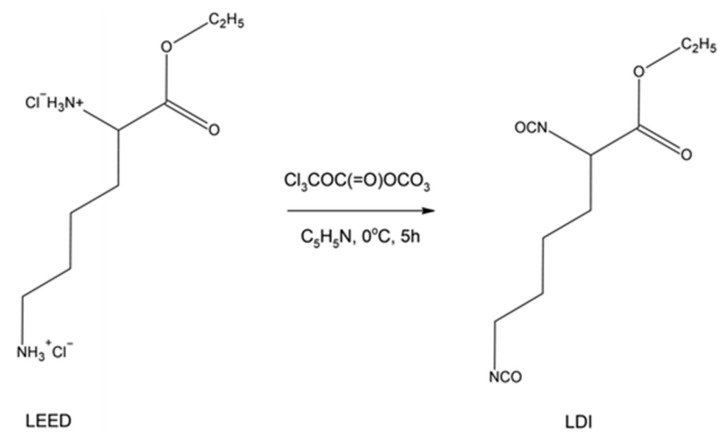
Synthesis of ethyl ester L-lysine diisocyanate (LDI) [66,87].

In a related study, Jian Han et al. [87] proposed a synthetic route for the preparation of the ethyl ester diisocyanate of L-lysine, which was subsequently applied in the synthesis of bio-based polyurethane materials. The polymerisation process was conducted in two stages using 1,4-butanediol and polycaprolactone diols (PCL) as co-reactants. The synthesis of L-lysine-derived diisocyanate was carried out according to the procedure shown in Figure 17.

Mechanical testing demonstrated that the resulting polyurethane exhibited a maximum tensile strength of 23 MPa and an elongation at break reaching 1700%. Owing to the presence of polycaprolactone (PCL) segments, which are inherently biodegradable polyesters, the obtained polyurethane materials exhibited a biodegradable character. Degradation of these systems yields non-toxic products, including L-lysine and α-hydroxycaproic acid.

Despite these favorable properties, the overall reaction yield was limited to approximately 50%. In addition, pyridine was required as a scavenger for hydrogen chloride generated during synthesis. These factors represent significant drawbacks for large-scale implementation, indicating that further optimization of reaction efficiency and elimination of toxic reagents such as pyridine are necessary prior to industrial application.

This class of biodegradable polyurethanes has been identified as a promising candidate for the fabrication of porous tubular scaffolds. In a related study, a biodegradable triblock oligomer, poly(lactic acid)–poly(ethylene glycol)–poly(lactic acid) (PLA-PEG-PLA), was synthesized and subsequently employed, together with ethyl ester L-lysine diisocyanate (LDI) and 1,4-butanediol (BDO) at identical stoichiometric ratios, to prepare a series of biodegradable polyurethane materials [88].

#### 2.2.5. Algae-Derived Isocyanates

The utilization of food-grade feedstocks in chemical synthesis is generally considered unfavorable due to the growing global demand for food, intensive agricultural land use, high water consumption and the resulting pressure on food production capacity. For this reason, algal biomass has attracted increasing interest as an alternative renewable resource. Its main advantages include rapid growth rates, minimal habitat requirements, high productivity and the ability to generate significant quantities of unsaturated fatty acids. In addition, residual components of microalgae, such as proteins, can be further valorised as polymer precursors.

Building on these advantages, a research group at the University of California developed a synthetic strategy for producing isocyanates from fatty acids derived from algal biomass. The approach is based on the generation of acyl azides under continuous-flow conditions, followed by a Curtius rearrangement (Figure 13). Initially, this methodology was applied to the synthesis of aliphatic diisocyanates and was subsequently extended to the preparation of linear alkyl and aromatic isocyanates from a range of organic acid substrates.

In the first stage, azelaic acid was produced from palmitoleic acid isolated from algae and subsequently used as a precursor for the synthesis of 1,7-heptamethylenediisocyanate (HpMDI). Continuous-flow processing was employed throughout the synthetic sequence to enhance operational safety and scalability. Azelaic acid was first converted into dimethyl azelate via Fischer esterification, followed by reaction with hydrazine monohydrate. Subsequent treatment with nitric acid led to the formation of the corresponding acyl azide, after which toluene was introduced into the flow stream. The intermediate was dried and heated to 85 °C under flow conditions to induce the Curtius rearrangement (Figure 8 and Figure 9).

Using this protocol, the target diisocyanate was obtained with a yield of approximately 80% and a production rate of about 500 mg h^−1^. From both environmental and safety perspectives, the use of hydrazine monohydrate—significantly less hazardous than anhydrous hydrazine—represents a clear advantage. Moreover, continuous-flow operation eliminates the risks associated with the accumulation and handling of high-energy, unstable acyl azide intermediates [89].

To date, the studies reported by these authors constitute the only published examples describing the synthesis of isocyanates derived from algal biomass [90]. Proposed methodologies represent a promising route toward the production of bio-based isocyanates with reduced environmental impact and potential economic viability. However, a key challenge for large-scale implementation remains the identification and cultivation of microalgal strains that combine high lipid content, rapid growth rates and high lipid productivity, all of which are critical parameters for efficient industrial processing.

#### 2.2.6. Saccharide-Based Isocyanates

Sugar-derived biomass provides a readily accessible source of renewable feedstocks for isocyanate synthesis, as it can be obtained from agricultural materials such as corn, soy and wheat. Owing to their structural diversity, low toxicity, hydrophilicity and comparatively low environmental impact relative to petroleum-derived chemicals, saccharides have attracted interest as precursors for polymer synthesis. Monosaccharides and disaccharides, including mannose, lactose and galactose, have been explored as renewable building blocks for the preparation of polymer intermediates [91,92,93].

**Figure 18:**
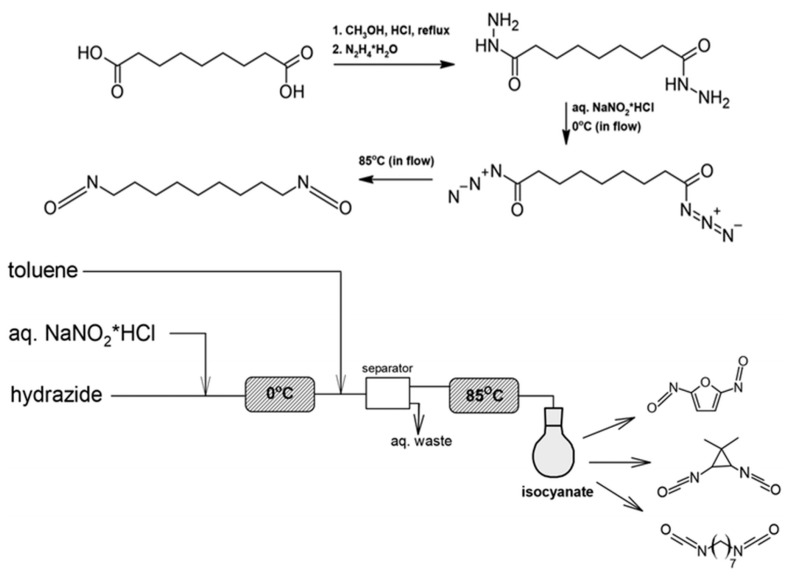
Continuous-flow reaction scheme for the synthesis of isocyanates from hydrazide intermediates [66,89].

Bachmann et al. [94] reported the synthesis of diisocyanates from dianhydrohexitols exhibiting three distinct stereochemical configurations derived from d-glucose, l-ido and d-mannose (Figure 18). Conversion of these substrates into the corresponding diisocyanates was achieved using two phosgenation-based approaches, namely cold and hot phosgenation. The reaction yield was found to be highly dependent on the stereochemical arrangement of the dianhydrohexitol backbone. In particular, phosgene addition was more efficient when the amino groups were oriented out of the molecular plane, resulting in the highest yield for the diisocyanate obtained from the l-ido configuration.

**Figure 19:**
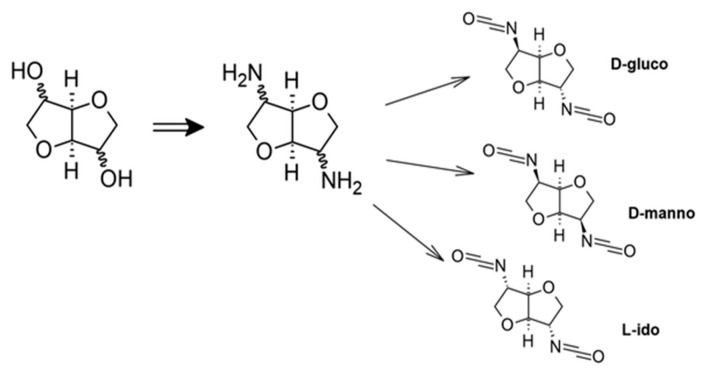
Monomer synthesis pathway for saccharide- and isosorbide-derived intermediates [66,94].

The synthesized diisocyanates were subsequently applied in polyaddition reactions to obtain new polyurethane materials. Although the study demonstrates the feasibility of using saccharide-derived substrates as renewable precursors for isocyanate synthesis, the reliance on phosgenation remains a significant limitation, highlighting the need for alternative phosgene-free synthetic routes (Figure 19).

Isosorbide has emerged as a particularly attractive renewable alternative to petroleum-derived building blocks due to its rigid bicyclic structure and high thermal stability. Zenner et al. reported the synthesis of a series of diisocyanates based on isosorbide as a renewable core structure. In addition, succinic anhydride was incorporated into the synthetic route owing to the competitive cost of bio-based succinic acid and its potential to reduce reliance on petrochemical reagents [95].

**Figure 20:**
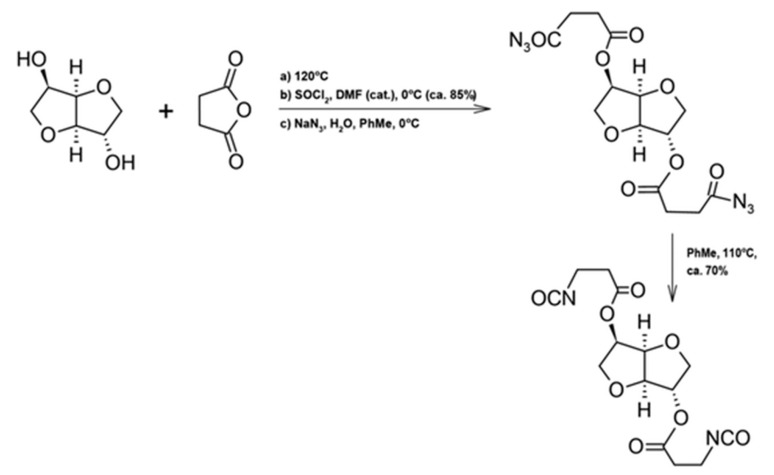
Synthesis of a bio-based diisocyanate derived from starch-based feedstocks [95].

Isosorbide-based diisocyanates have been reported as renewable alternatives for polyurethane synthesis and successfully applied in the preparation of thermoplastic polyurethanes; however, their relevance to polyurethane recyclability remains indirect and is primarily associated with the resulting network structure rather than end-of-life processing strategies (Figure 20) [95].

Starch obtained from non-edible field corn has been utilized by Covestro for the development of aliphatic pentamethylene diisocyanate (PDI). This product represents the first commercially available diisocyanate containing approximately 70% renewable carbon. The production process for this bio-based isocyanate is schematically illustrated in Figure 20 [96].

#### 2.2.7. Cashew Nut Shell Liquid as an Alternative Feedstock for Isocyanate Synthesis

Non-edible biomass-derived resources, including phenols, hemicelluloses and other lignocellulosic components, represent attractive feedstocks for chemical synthesis because they do not compete with food production. One such renewable source of aromatic monomers is cashew nut shell liquid (CNSL), which belongs to the class of naturally occurring phenolic compounds. CNSL is generated as a by-product during cashew nut processing and is considered a valuable renewable raw material. Its main constituents include anacardic acid, cardanol, cardol and 2-methylcardol.

Among these components, cardanol has found widespread industrial use in the production of coatings, resins and surfactants owing to its phenolic functionality and long aliphatic side chain [97]. Cardanol-containing novolac resins can be further converted into cyanate ester systems, providing access to thermosetting polymer networks.

**Figure 21:**
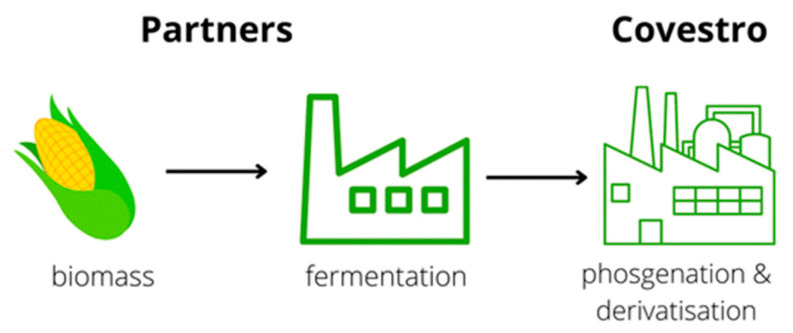
Industrial production process of Covestro’s Desmodur^®^ eco N 7300 bio-based polyisocyanate [66,96].

**Figure 22:**
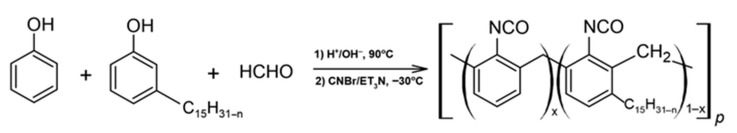
Schematic representation of the synthesis of an isocyanate-functional phenolic resin via polycondensation of phenol and alkylphenol with formaldehyde, followed by functionalization with isocyanate groups [66,99].

Nair et al. [98] investigated the synthesis of cardanol-modified novolac resins and systematically evaluated the influence of cardanol content on the thermal stability of the resulting materials (Figure 21 and Figure 22). Upon curing, the cyanate ester groups formed phenolic–triazine network structures characterized by good thermal stability. However, increasing the proportion of cardanol within the novolac resin led to a progressive decrease in the thermal stability of the cured polymers, reflecting the plasticising effect of the flexible aliphatic side chains.

Kulkarni et al. [99] reported the synthesis of cyanate ester monomers containing pentadecyl-substituted cyclohexyl moieties [100]. The obtained compounds—1,1-bis(4-cyanatophenyl)-3-pentadecylcyclohexane and 1,1-bis(4-cyanatophenyl)cyclohexane—were structurally characterized using Fourier-transform infrared spectroscopy (FTIR) and proton nuclear magnetic resonance (^1^H NMR). Compared with commercially available cyanate ester systems, these monomers exhibited improved processability, reduced melting temperatures and shorter curing times, highlighting the beneficial effect of aliphatic substitution on their thermal and processing behavior.

### 2.3. Additives for Polyurethanes

#### 2.3.1. Blowing Agents

The production of rigid polyurethane foams requires the incorporation of blowing agents alongside the polyol and polyisocyanate components. With increasing awareness of the environmental impact associated with these substances, successive generations of blowing agents have been developed. The first generation comprised fully halogenated chlorofluorocarbons, which are liquids characterized by a high ozone depletion potential. These were later replaced by second-generation hydrochlorofluorocarbons, which exhibit reduced but still significant environmental impact [101].

Cellular polyurethane materials are formed through the action of blowing agents that generate gas bubbles within the reacting polymer matrix. Water is the oldest and most widely used chemical blowing agent; it reacts with isocyanate groups to release carbon dioxide while simultaneously forming rigid polyurea domains. In addition to water, auxiliary blowing agents—typically low-boiling liquids—are incorporated into foam formulations to support the expansion process. These compounds absorb heat generated during the highly exothermic polyurethane-forming reactions, evaporate during foaming, and provide additional gas volume, thereby enabling foam expansion to lower apparent densities [102].

Blowing agents play a critical role in polyurethane foam production by enabling the formation of a controlled cellular structure through in situ gas generation. Their presence directly influences thermal and acoustic insulation performance, foam density and mechanical strength, allowing materials to be tailored to specific application requirements. Both the type and concentration of blowing agent significantly affect foam quality, performance and production cost. In general, blowing agents used in polyurethane systems can be classified into two main categories: physical and chemical blowing agents [103].

(A) Physical Blowing Agents

Many physical blowing agents are associated with adverse environmental effects, including contributions to global warming and ozone depletion. As a result, increasing regulatory pressure has driven the development and implementation of fourth-generation hydrofluoroolefins, which are characterized by negligible ozone depletion potential and significantly reduced global warming potential [6,103].

Physical blowing agents such as cyclopentane and 1,1-dichloro-1-fluoroethane vaporize under processing conditions, generating gas that produces the cellular structure of polyurethane foams. Cyclopentane is particularly widely used in rigid polyurethane foams owing to its favorable thermal insulation performance. Although more environmentally benign alternatives, including certain hydrofluorocarbons, have gained prominence, physical blowing agents continue to play an important role in controlling foam density and cell morphology in polyurethane systems [103].

(B) Chemical Blowing Agents

Chemical blowing agents generate gas as a result of chemical reactions occurring during polyurethane formation, most commonly through the reaction between isocyanate groups and water, which leads to the release of carbon dioxide. This mechanism is widely applied in the production of both flexible and rigid polyurethane foams, enabling controlled cell formation and contributing to improved material performance. In contrast to physical blowing agents, chemical blowing agents participate directly in the reaction mechanism, although they are not consumed as discrete components in the final polymer structure [103].

#### 2.3.2. Catalysts

Catalysts play a critical role in polyurethane synthesis by accelerating chemical reactions without being consumed. In polyurethane systems, they regulate the relative rates of the reactions between polyols and isocyanates, thereby controlling polymer formation, foaming behavior and final material structure. Catalysts used in polyurethane chemistry are generally classified into two main categories: amine-based catalysts and metal-based catalysts.

##### Amine Catalysts

Tertiary amines are among the most commonly employed catalysts for promoting both the isocyanate–hydroxyl (gelling) and isocyanate–water (blowing) reactions. Typical examples include solutions of 1,4-diazabicyclo[2.2.2]octane and bis(2-dimethylaminoethyl) ether diluted in dipropylene glycol, which are widely used in the production of flexible and rigid polyurethane foams. These catalysts enable effective process control and contribute to the formation of foams with favorable mechanical properties [103].

The catalytic activity of tertiary amines is primarily governed by their basicity and the degree of steric hindrance around the nitrogen atom. Higher basicity and lower steric hindrance generally result in increased catalytic activity. In addition, hydrogen-bonding capability and the spatial arrangement of active sites influence catalytic selectivity toward either gelling or blowing reactions [103].

##### Metal Catalysts

Metal-based catalysts, particularly organotin compounds, are frequently used to accelerate specific reactions in polyurethane systems, especially in rigid foam formulations. These catalysts provide precise control over reaction kinetics, facilitating the formation of foams with stable cellular structures and enhanced thermal performance. Metal catalysts are often used in combination with amine catalysts to fine-tune the balance between polymerization and foaming processes.

One of the most widely applied metal catalysts is stannous octoate, which offers effective control over polymerization rates. However, inappropriate dosing can lead to defects in the final foam structure, underscoring the importance of careful catalyst selection and optimization in polyurethane formulations [103].

#### 2.3.3. Surfactants

Surfactants, commonly referred to as cell stabilizers, are essential additives in polyurethane foam production, where they serve to stabilize the developing cellular structure and prevent foam collapse. By reducing surface tension at the gas–polymer interface, surfactants promote uniform cell nucleation and growth, resulting in foams with homogeneous and stable morphology. Their presence contributes to improved thermal and acoustic insulation performance as well as enhanced mechanical properties.

In addition, surfactants play a key role in controlling foam volume and density, thereby influencing overall product quality. Both the type and concentration of surfactant have a pronounced effect on foam performance; insufficient stabilizer content can lead to rapid cell coalescence, structural instability and collapse of the foam during formation [104].

Surfactants derived from renewable resources have attracted increasing attention as alternatives to conventional petroleum-based stabilizers. In particular, surfactants based on neem essential oil have been investigated for application in polyurethane foam production. Neem seed oil contains triglycerides and free fatty acids that can act as surface-active species, effectively reducing surface tension during foaming and promoting the formation of a uniform and stable cellular structure. Their incorporation into polyurethane formulations may enhance foam flexibility and stiffness by influencing the balance between soft and hard segments within the polymer matrix. In addition, neem oil and selected nanoparticulate additives exhibit antimicrobial properties, which may impart additional functional benefits to the resulting foams. Although systematic studies on the mechanical performance of polyurethane foams produced with neem-based surfactants remain limited, their renewable origin and potential for reduced environmental impact highlight their promise for future industrial applications [105].

Polyurethane foams derived from orange peel waste and orange-seed oil have also been reported, although these materials play a comparatively minor role in surfactant applications relative to neem-based systems. From an environmental perspective, neem oil production is generally associated with lower greenhouse-gas emissions than the manufacture of petrochemical surfactants, primarily due to its biogenic carbon content. Moreover, neem tree cultivation can contribute to carbon sequestration. The extraction of neem oil typically requires lower energy input than petrochemical refining processes, while petrochemical surfactants may exhibit higher ecotoxicity as a result of chemical runoff or accidental release [106].

Among bio-based alternatives, castor oil remains the most widely used renewable surfactant in polyurethane foam formulations, frequently serving as a direct replacement for petrochemical surfactants while offering both economic and environmental advantages [105].

#### 2.3.4. Flame Retardants

Polyurethane materials are inherently flammable, which poses significant safety concerns, particularly in applications exposed to elevated temperatures or open flames. To mitigate fire-related risks and improve material safety, flame retardants are commonly incorporated into polyurethane formulations. These additives are generally classified into halogenated, phosphorus-based and non-halogenated systems, depending on their chemical composition and mode of action [103].

An increasingly attractive strategy for enhancing fire resistance involves the application of surface-coating flame-retardant technologies. Rather than modifying the bulk composition of the polymer, this approach provides fire protection through the formation of a thermally stable char layer at the material surface, which acts as a physical barrier and inhibits flame propagation. Such surface-based solutions offer the advantage of improving fire performance while preserving the intrinsic mechanical and thermal properties of rigid polyurethane foams, representing a promising pathway for safer and more durable foam applications (Table 3 and Table 4) [107].

**Table 3:** Common flame retardants used in polyurethane foams [108,109,110,111].

Flame Retardant	Chemical Name	Chemical Formula	Literature Reference
TDCPP	Tris(1,3-dichloro-2-propyl) phosphate	C_9_H_15_Cl_6_O_4_P	[108]
DMMP	Dimethyl methyl phosphonate	C_3_H_9_O_3_P	[109]
APP	Ammonium polyphosphate	(NH_4_PO_3_)_n_	[110]
EG	Expandable graphite	C (or C_42_(HSO_4_)(H_2_SO_4_)_2_)	[110]
AlPi	Aluminum diethyl phosphinate	C_8_H_20_O_4_P_2_Al	[111]

**Table 4:** Bio-based flame retardants used in polyurethane foams [108].

Flame Retardant	Chemical Name/Source	Reference
Alginic acid coating with hydroxyapatite (HAP)	Sodium alginate + hydroxyapatite	[112]
Lignin + sugarcane bagasse ash	Kraft lignin + bagasse ash	[113]
Kraft lignin	Kraft-type lignin	[114]
Bio-based melamine–formaldehyde resin	Glycerol/lignosulfonate-modified MF resin	[115]
Bio-polyol from rapeseed oil + phosphorus FR	Rapeseed-oil-based bio-polyol + DMMP	[116]

##### Hydrogel Coatings

Hydrogel-based coatings are considered environmentally benign flame-retardant solutions owing to their high water content, viscosity and hydrophilic character. When exposed to flame or elevated temperatures, the evaporation of water from the hydrogel matrix absorbs a substantial amount of heat, thereby reducing the temperature at the material surface. Simultaneously, the released water vapor acts as a physical barrier that limits oxygen diffusion to the polymer surface, effectively suppressing combustion processes [117].

##### Halogenated Flame Retardants

Halogenated flame retardants, including chlorine- and bromine-containing compounds, function primarily by forming a protective layer during combustion and by interfering with radical-driven flame-propagation reactions. Although these systems are highly effective, growing environmental and health concerns—particularly related to toxic combustion products and persistence in the environment—have led to their gradual replacement by safer and more sustainable flame-retardant alternatives [103].

##### Phosphorus-Based Flame Retardants

Phosphorus-containing compounds are widely regarded as among the most promising flame retardants for polyurethane materials due to their high efficiency and comparatively low emission of toxic gases and smoke during combustion [108]. This broad class includes red phosphorus [107], inorganic phosphates and hybrid organic–inorganic structures in which phosphorus atoms are chemically incorporated into the molecular framework [118].

Phosphorus-based flame retardants have increasingly replaced halogenated systems as a result of their reduced environmental impact and strong flame-retardant performance. These additives promote the formation of a protective char layer on the polymer surface upon heating, thereby inhibiting flame propagation. In addition, phosphorus-containing compounds can reduce ignition temperature and decrease the heat-release rate, significantly improving the fire safety of polyurethane products. Their chemical stability and relatively low toxicity make them particularly attractive for industrial applications.

A commonly used phosphorus-based flame retardant in polyurethane formulations is tris(1-chloro-2-propyl) phosphate, which is valued for its high flame-retardant efficiency [103]. Phosphorus-containing systems may act in both the condensed and gas phases. In the condensed phase, they enhance char formation, limiting heat and oxygen transfer to the underlying polymer. During thermal decomposition, phosphorus compounds often generate phosphoric acid, which condenses to form pyrophosphate (P–O–P) structures while releasing water vapor, thereby diluting the oxidizing gas phase. In addition, certain phosphorus-based species function as radical scavengers in the gas phase. When covalently bonded to the polymer backbone, these compounds can further reduce heat release.

A notable limitation of phosphorus-containing polyurethane systems is the accelerated onset of thermal degradation associated with built-in phosphorus segments. The lower bond energy of P–O linkages compared with C–O bonds in the polyurethane backbone renders these structures more susceptible to early thermal decomposition.

##### Additives for Flame Retardant Systems

Non-halogenated mineral flame retardants, such as aluminum trihydrate (ATH) and magnesium hydroxide, act by releasing water upon heating, which cools the combustion zone and dilutes flammable gases, thereby reducing flame spread. These compounds are non-toxic and thermally stable, making them attractive components of environmentally benign polyurethane formulations. Their incorporation can improve overall product performance and enable polyurethane materials to meet regulatory and application requirements in sectors such as construction, automotive and packaging [103].

Aluminosilicates are also widely employed as modifiers in polyurethane systems. These naturally occurring minerals are abundant, cost-effective and well suited for enhancing the functional properties of polyurethane foams. Typically, clay-based nanoparticles are dispersed in the polyol component prior to foaming. Their incorporation leads to significant improvements in mechanical properties, including compressive and tensile strength, Young’s modulus and elongation at break.

In addition to mechanical reinforcement, aluminosilicates markedly improve barrier properties, which are critical for thermal stability and flame resistance. For example, the addition of approximately 4 wt.% clay has been shown to reduce oxygen permeability by about 50%. Permeability to water vapor and dichloromethane also decreases with increasing clay content, up to approximately 20 wt.%. This behavior is attributed to the formation of a “tortuous path” created by the dispersed nanoclay platelets, which restricts gas diffusion through the polymer matrix.

From the perspective of flammability and thermal stability, the reduced mass and heat transfer associated with this barrier effect slows polymer degradation. The delayed release of volatile decomposition products further suppresses flame propagation when compared with unfilled polyurethane materials.

In rigid polyurethane foams, nanoclays have been shown to be particularly effective in improving thermal stability. However, the magnitude of this improvement varies across reported studies, which can be primarily attributed to differences in filler dispersion within the polymer matrix as well as to the degree of clay intercalation or exfoliation.

**Figure 23:**
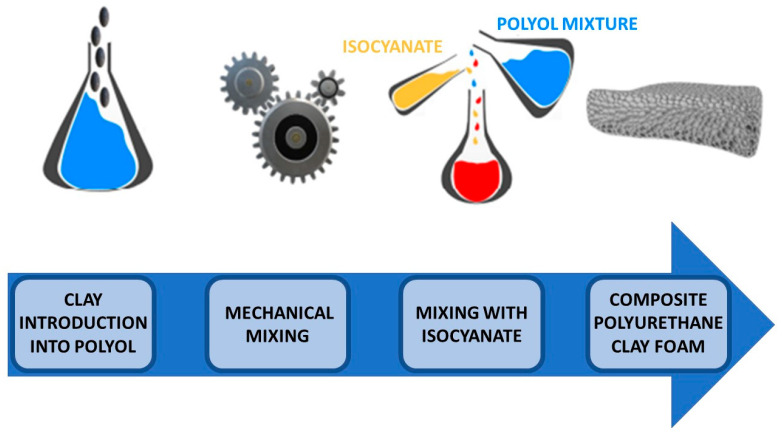
Conventional method of incorporating clay into polyurethane foams [119].

The various dispersion states of clay platelets in the polyurethane matrix are schematically illustrated in Figure 23. The effectiveness of clay-based modification is strongly dependent on the quality of dispersion and can be significantly enhanced through the application of sonication or other dispersion-promoting techniques.

**Figure 24:**
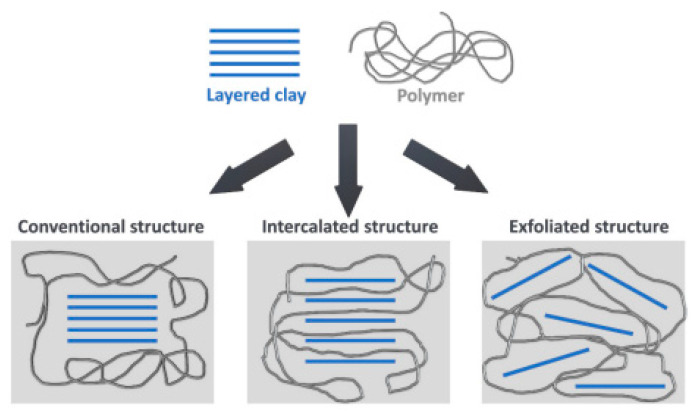
Schematic representation of clay structures in a polymer matrix at different dispersion levels.

Heidarian et al. [119] examined clay dispersion in polyol systems subsequently used for polyurethane synthesis using optical microscopy. The introduction of a 30 min sonication step substantially reduced both the number and size of nanoclay agglomerates compared with samples prepared solely by mechanical stirring (Figure 24). Improved dispersion of clay within the polyurethane matrix resulted in the formation of a more effective thermal barrier, leading to enhanced thermal stability of the resulting foams.

### 2.4. Adhesion Promoters

One of the key advantages of polyurethane materials is their inherently strong adhesion to a wide variety of substrates. This property underpins the extensive use of polyurethanes across multiple industrial sectors, including automotive manufacturing, construction and footwear production. To further enhance adhesion performance—particularly under fluctuating environmental conditions—polyurethane formulations are often modified with adhesion-promoting additives. These compounds improve the strength and stability of interfacial interactions between polyurethane materials and diverse substrate surfaces [103].

Polypropylene glycol (PPG) is among the most widely used adhesion-promoting additives in polyurethane formulations. Owing to its chemical structure, PPG enhances the adhesion of polyurethane systems to metals, wood and various polymeric substrates. When incorporated as a polyol component, PPG additionally improves the elasticity and adhesive performance of polyurethane foams. Its application is particularly prevalent in the automotive and construction industries. Beyond adhesion enhancement, PPG contributes to improved mechanical properties, foam homogeneity and long-term durability. By selecting appropriate PPG types and molecular weights, processing behavior can be tailored and the final performance characteristics of polyurethane materials can be precisely adjusted [103].

Pressure-sensitive adhesives (PSAs) represent another important class of polyurethane-based materials. PSAs are viscoelastic adhesives capable of forming bonds with minimal applied pressure and within a very short contact time. They must create aggressive and durable tack while also allowing clean removability without leaving residue. In PSAs, the viscous component is responsible for wetting and bonding, while the elastic component ensures cohesive strength and peel resistance.

Pressure-sensitive adhesives (PSAs) are widely employed in packaging tapes, labeling systems and repositionable adhesive products, as well as in applications across the food, pharmaceutical, security packaging and automotive sectors. In addition, PSAs show considerable potential in biomedical applications, including medical plasters, wound dressings, biomedical electrodes and transdermal drug-delivery patches.

The three primary viscoelastic properties required for PSA performance are:

- tack—instantaneous bonding ability enabling rapid adhesion and substrate wetting,
- shear adhesion—resistance to deformation and creep under shear stress,
- peel strength—the ability to be cleanly removed without leaving residues.

PSAs can be categorized based on composition and processing routes, including polyurethane PSAs, acrylic PSAs, polysiloxane PSAs, and polyisobutylene PSAs. Polyurethane PSAs (PU-PSAs) typically consist of three segments:

(1) soft segments with low glass-transition temperatures (responsible for tack),
(2) hard segments with defined melting transitions (providing cohesive strength),
(3) crosslinking components, enabling structural integrity and controlled adhesion.

PU-PSAs are valued for their high performance, environmental friendliness, and ease of application. They also exhibit good thermal and chemical resistance, favorable low-temperature properties, and higher hydrophilicity compared with acrylic or rubber-based PSAs. However, PU-PSAs may exhibit limitations such as reduced tack, lower peel strength and relatively high moisture permeability. These drawbacks can be mitigated by optimizing formulation parameters, including adjustment of the HDI/HDI-trimer ratio, modification of crosslinking agents and precise control of interactions between hard and soft segments within the polymer structure.

Fuensanta et al. [120] investigated PU-PSAs synthesized from mixtures of polyether polyols and polypropylene glycols (PPG) of various molecular weights, demonstrating their effect on viscosity, cohesion, and adhesion performance. Baron et al. [121] described grafted polyurethanes containing crystallizable segments and PPG mixtures, enabling precise tuning of adhesive behavior. Akram et al. [122] studied the influence of macrodiol type and molecular weight on tack, peel strength, and cohesive properties of PU-PSAs, identifying optimal structural parameters for high-performance pressure-sensitive adhesives.

### 2.5. Thermal and UV Stabilizers

Despite the generally good resistance of polyurethanes to environmental factors arising from their chemical structure, these materials remain susceptible to thermal and ultraviolet (UV) degradation. Prolonged exposure to elevated temperatures or UV radiation can lead to chemical bond scission, deterioration of mechanical properties, discoloration and, ultimately, embrittlement. To extend service life and ensure long-term performance, thermal and UV stabilizers are commonly incorporated into polyurethane formulations. These additives mitigate degradation processes and help preserve both the functional and aesthetic properties of polyurethane materials during processing and use [103].

#### 2.5.1. Thermal Stabilizers

Thermal stabilizers are employed to protect polyurethanes against degradation induced by elevated temperatures during processing or prolonged service. Manufacturing operations involving heat, such as molding or extrusion, can initiate thermo-oxidative reactions that adversely affect material properties. Commonly used thermal stabilizers include phosphites and phenolic antioxidants, which suppress oxidation reactions and inhibit radical chain processes responsible for thermal degradation [103].

Phosphite stabilizers function primarily as secondary antioxidants by decomposing hydroperoxides formed during polymer autoxidation, thereby preventing further degradation and extending the effectiveness of primary antioxidants. Sterically hindered phenolic antioxidants, in turn, act as radical scavengers, providing protection against thermo-oxidative degradation while maintaining color stability and mechanical integrity. Owing to their low volatility, good compatibility and high extraction resistance, such stabilizers are widely applied in polyurethane systems as well as in other polymeric materials [123,124,125].

#### 2.5.2. UV Stabilizers

UV stabilizers are incorporated into polyurethane formulations to protect materials exposed to sunlight, particularly ultraviolet radiation. UV-induced degradation occurs primarily through photochemical reactions that generate free radicals and lead to bond cleavage within the polymer backbone. To counteract these effects, UV stabilizers either absorb harmful radiation or neutralize reactive species formed during photo-oxidation, thereby preserving mechanical properties and surface appearance during outdoor exposure [103].

Two principal classes of light stabilizers are used in polyurethane systems: ultraviolet absorbers (UVAs) and hindered amine light stabilizers (HALS). UV absorbers, such as benzotriazole derivatives, protect polymers by absorbing UV radiation and dissipating the energy as heat, effectively preventing discoloration, yellowing and surface degradation. HALS stabilizers operate through a different mechanism, scavenging free radicals generated during photo-oxidation and inhibiting degradation reactions at the polymer surface. As a result, HALS are particularly effective in maintaining gloss, preventing microcracking and suppressing chalking in coatings and adhesive applications [123,124,125].

High–molecular-weight HALS compounds are especially valuable due to their low volatility and long-term stabilizing efficiency, providing sustained protection against photo-oxidative degradation in polyurethane-based plastics, fibers and pressure-sensitive adhesive systems. Major classes of UV Stabilizers are presented in Table 5.

### 2.6. Plasticizers

Plasticizers are important additives in polyurethane formulations, where they are used to enhance flexibility, softness and resilience of polymeric materials. Their primary function is to increase the mobility of polymer chains by enlarging the free volume within the polymer matrix, which results in reduced hardness, lower stiffness and improved elasticity. Plasticizers play a particularly important role in the production of flexible polyurethane foams, elastomers, synthetic leather and polyurethane coatings, where excessive rigidity or brittleness must be avoided.

Phthalates have historically been among the most widely used plasticizers in polyurethane and other polymer systems due to their effectiveness in lowering the glass transition temperature (T_g_) and improving mechanical flexibility. However, increasing environmental and health concerns have led to regulatory restrictions on their use in many regions. As a consequence, alternative plasticizers—such as ester-based and other non-phthalate systems—are increasingly being adopted, offering safer and more sustainable solutions for enhancing the flexibility of polyurethane materials.

In addition to improving flexibility, plasticizers contribute to enhanced crack resistance, abrasion resistance and processing performance. Owing to these combined effects, they are widely employed in polyurethane applications across the automotive, furniture, footwear and packaging industries [126].

### 2.7. Solvents

Solvents are used in polyurethane systems to adjust viscosity, improve processability and facilitate the application of coatings, adhesives and resin formulations. By dissolving or dispersing raw materials such as polyols and isocyanates, solvents promote improved homogenization and more uniform component distribution, which enhances handling, flow behavior and application quality. Their use is particularly important in polyurethane coatings and paints, where controlled viscosity, leveling and film formation are critical.

In certain formulations, solvents may also be employed for cleaning cured polyurethane residues or for generating porosity in specific processing routes. The selection of an appropriate solvent has a direct influence on processing efficiency as well as on the final properties of the cured polyurethane material. Consequently, solvents must be carefully chosen to achieve the desired rheological and application characteristics without adversely affecting the mechanical or chemical performance of the final product [103]. Major classes of solvents used in different polyurethane applications are presented in Table 6.

## 3. Applications of Polyurethane Materials

### 3.1. Clothing, Household Appliances and Automotive Applications

The discovery that polyurethanes can be processed into fine filaments enabled their incorporation into textile applications, initially through blending with nylon to produce lighter and more elastic fabrics. Continued material development led to the emergence of spandex fibers, polyurethane coatings and thermoplastic polyurethane elastomers. Advances in processing technologies have further expanded the range of polyurethane-based textile materials, allowing the manufacture of synthetic leather, coated fabrics, sportswear and a wide variety of functional clothing accessories. These materials combine elasticity, durability and comfort, making polyurethanes an important component of modern apparel applications [127].

Polyurethanes play a critical role in the manufacture of household appliances used in everyday life. Their most significant application in this sector is rigid polyurethane foam employed as thermal insulation in refrigerators and freezers. Rigid polyurethane foam is a cost-effective and indispensable insulating material that enables appliance manufacturers to meet stringent energy-efficiency requirements. Its excellent thermal performance arises from a fine, closed-cell morphology combined with low-thermal-conductivity blowing agents that effectively limit heat transfer [127].

In addition to household appliances, polyurethanes are widely used in the automotive industry. Flexible polyurethane foams are commonly applied in seating systems, while rigid foams serve as thermal and acoustic insulation materials. Key advantages of polyurethane materials in automotive applications include their low density coupled with high mechanical strength. These properties contribute to vehicle weight reduction, improved fuel efficiency, extended driving range and enhanced passenger safety [127].

Polyurethanes play a key role in the production of modern engineered wood and composite wood products. Polyurethane-based binders are widely used to bond lignocellulosic materials in oriented strand board, medium-density fiberboard, laminated veneer lumber, long-fibre wood composites, strawboard and particleboard. These adhesives provide strong and durable bonding, contributing to improved mechanical performance, moisture resistance and dimensional stability of composite wood materials [127].

Polyurethane materials—predominantly in the form of flexible foams—are among the most widely used materials in household applications, including furniture, bedding and carpet underlays. As cushioning materials in upholstered furniture, flexible polyurethane foams provide enhanced comfort, durability and mechanical support, contributing to prolonged service life and improved user experience [127].

Polyurethane foam packaging systems provide cost-effective, custom-fitted cushioning solutions designed to protect products during transportation. Such materials are widely used for securing sensitive items, including electronic equipment, medical diagnostic devices, fragile glassware and large industrial components. On-site molding of polyurethane foam packaging offers a flexible and efficient approach, allowing packaging to be tailored precisely to individual shipments while reducing handling time and overall logistics costs [127].

### 3.2. Civil Engineering

Modern construction requires materials that combine high mechanical performance with low weight, durability, ease of installation and design flexibility. Polyurethane materials meet these requirements while additionally contributing to reduced energy consumption in buildings, thereby supporting resource efficiency and environmental protection. Owing to their favorable strength-to-weight ratio, excellent insulation performance, durability and versatility, polyurethanes are widely applied in construction and civil-engineering applications. Their cost effectiveness and contribution to indoor comfort have made polyurethane-based components standard materials in contemporary residential and commercial buildings [127].

Polyurethanes are employed throughout building envelopes and interior systems. In flooring applications, flexible polyurethane foam underlays are used to cushion carpets and improve comfort. In roofing systems, reflective polymeric coatings applied over rigid polyurethane foam reduce solar heat gain, helping to maintain lower indoor temperatures and decrease energy demand. Polyurethane-based building elements also expand architectural design possibilities in both new construction and renovation projects. Foam-core panels are available in a wide range of colors and profiles for walls and roofs, while foam-core entrance and garage doors are produced in various finishes and styles [127].

The use of polyurethanes as coatings, adhesives, sealants and elastomers has enabled numerous high-value construction applications due to their favorable mechanical, chemical and physical properties. Polyurethane sealants provide durable, airtight joints, whereas polyurethane adhesives offer strong and reliable bonding to diverse substrates. In coating applications, polyurethanes must exhibit excellent adhesion, high chemical resistance, favorable curing behavior, thermal flexibility and sufficient scratch resistance [128,129].

For high-performance coating systems, nanomaterials such as titanium dioxide or silicon dioxide are often incorporated to impart anticorrosive properties, improve durability and enhance aesthetic appearance. Despite these advantages, limitations such as reduced impact resistance and sensitivity to UV radiation in prolonged outdoor exposure may restrict the use of certain polyurethane coatings [127].

Polyurethane materials are also widely applied in flooring systems, both as carpet underlays and as protective topcoats. Flexible polyurethane foam used as carpet underlayment extends carpet lifetime, preserves appearance, improves comfort and reduces noise transmission in residential and commercial environments [127]. In addition, polyurethane coatings are commonly used to protect wood, parquet and concrete floors, providing abrasion and solvent resistance while remaining easy to clean and maintain. Such finishes enhance the service life of new flooring systems and enable refurbishment of worn surfaces to restore their original appearance [127].

Potting and encapsulation systems based on polyurethanes are designed to deliver a broad range of physical, thermal and electrical properties. These materials protect electronic components by providing excellent dielectric behavior, strong adhesion and resistance to solvents, moisture and extreme temperatures, making them suitable for demanding construction-related electrical applications [127].

### 3.3. Electronics

Unfoamed polyurethane systems, commonly referred to as potting compounds, are extensively used in the electrical and electronics industries for encapsulation, sealing and insulation of sensitive components. Typical applications include microelectronic devices, underwater cables and printed circuit boards, where protection against mechanical stress, moisture and chemical exposure is required. Polyurethane potting materials provide excellent dielectric properties, strong adhesion and long-term stability under demanding operating conditions [127].

In addition, polyurethane foams have been explored in wave-absorbing and electromagnetic-interference (EMI) shielding applications. Microwave-absorbing composites based on polyurethane foams exhibit high electromagnetic absorption efficiency and tunable properties, making them promising materials for stealth, counter-stealth and advanced electronic applications [130].

**Figure 25:**
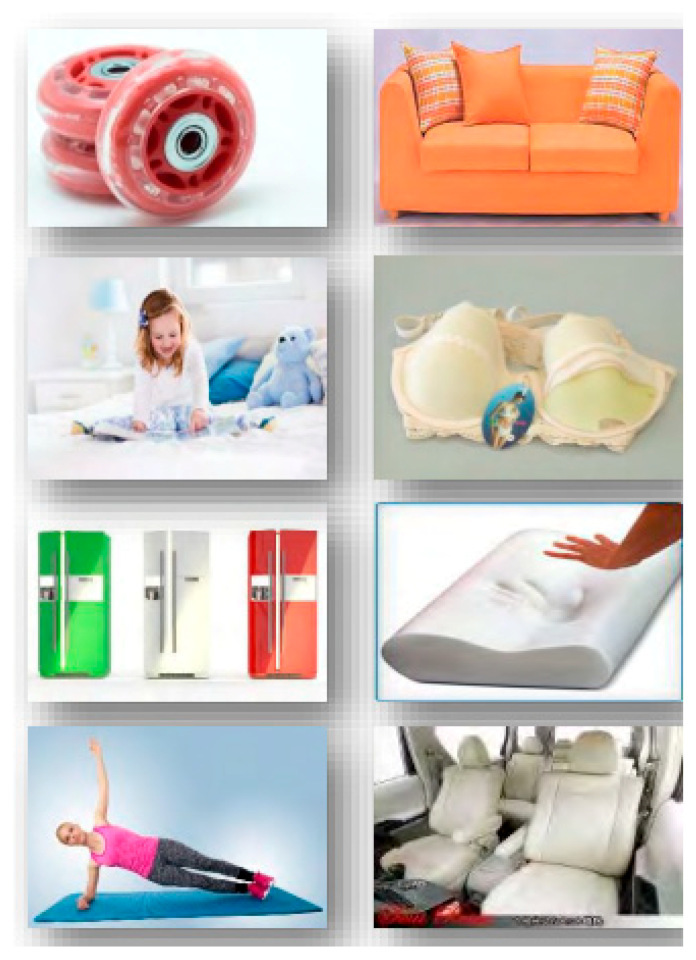
Representative applications of polyurethane [130].

Selected applications of polyurethane-based materials in the furniture and construction sectors, highlighting their structural and functional versatility, are presented in Figure 25.

**Figure 26:**
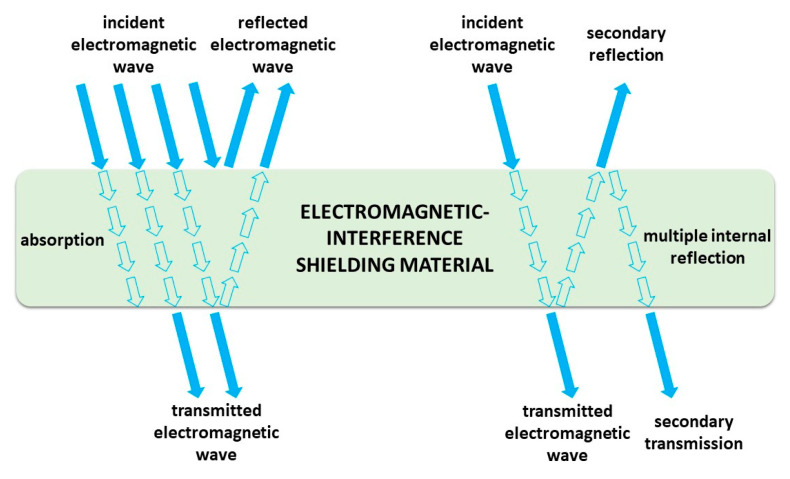
Schematic of the EMI Shielding Mechanism [130].

A schematic representation of the EMI shielding mechanism in polyurethane foams is shown in Figure 26.

Composite polyurethane (PU) foams are well suited for absorbing-type electromagnetic interference (EMI) shielding owing to their low density, high processability and inherent corrosion resistance. The EMI shielding effectiveness (SE) of PU foam composites can reach values of 50–60 dB, which significantly exceeds the ~20 dB threshold typically required for commercial applications. To impart electrical conductivity, a variety of conductive fillers—including carbon black, graphene and carbon nanotubes (CNTs)—have been incorporated into polyurethane matrices.

Rigid polyurethane foams designed for wave-absorption applications are commonly produced by introducing absorbing agents into rigid PU systems during in-mold foaming. The ability to precisely control density, mechanical strength and hardness through formulation design, combined with ease of shaping and processing, makes these materials particularly attractive for use in stealth and defense-related technologies. However, rigid PU foams may exhibit brittleness and limited fracture resistance, especially in large-scale structures, which can lead to cracking under mechanical stress.

Flexible polyurethane foams for electromagnetic wave absorption are typically prepared by reactive foaming, followed by impregnation with absorbing agents and subsequent drying. These materials are primarily employed in anechoic chambers. Although flexible foams generally display lower environmental stability—owing to water absorption, reduced machining precision and limited weather resistance—they offer advantages such as low density, high elasticity, broadband absorption performance and excellent vibration damping. These properties provide significant application potential despite their susceptibility to mechanical damage.

With the rapid expansion of electronics-related fields, including communication, computing and automation, there is an increasing demand for materials with high electrical conductivity. The growing prevalence of electromagnetic interference has intensified environmental and technological concerns, positioning conductive polyurethane composites as promising candidates for EMI shielding applications [33]. Electrical conductivity in such systems is commonly enhanced through the incorporation of fillers such as carbon black, graphene and CNTs [34]. Compared with zero-dimensional carbon black and three-dimensional graphite, one-dimensional carbon fibers and carbon nanotubes, as well as two-dimensional graphene, possess higher aspect ratios. As a result, polyurethane foams containing these fillers typically achieve higher electrical conductivity at comparable filler loadings.

Figure 26 presents a comparative overview of carbon-based conductive fillers with respect to their EMI shielding performance [130].

### 3.4. Marine Sector Applications

Polyurethane–epoxy resin systems are widely used in marine applications to seal boat hulls against water ingress, weathering and corrosion, while also reducing surface roughness and hydrodynamic drag. These properties contribute to extended service life and improved performance of marine vessels. In addition, rigid polyurethane foams are employed for thermal and acoustic insulation in marine structures, offering excellent abrasion and tear resistance as well as enhanced load-bearing capacity while adding minimal weight.

Thermoplastic polyurethane (TPU) is also well suited for marine applications due to its flexibility, mechanical durability and ease of processing. Typical uses include cable and wire coatings, engine hoses, drive belts, hydraulic lines, seals and molded boat components [103].

Waterborne polyurethanes (WPUs) have gained a substantial share of the corrosion-resistant coatings market owing to their environmentally favorable profile, strong adhesion to substrates and good stability under ambient conditions. The presence of polar segments incorporated into the polymer backbone enhances dispersion in water and solubility in polar solvents, enabling a significant reduction in volatile organic compound (VOC) emissions. Ongoing research focuses on incorporating additional functional components to further improve the corrosion resistance of WPU-based coatings for marine environments [131].

### 3.5. Biomedical Applications

Biomedical applications often require materials with a high content of bio-based components, as such systems are generally associated with improved biocompatibility and reduced risk of adverse biological responses. In polyurethane systems, the fraction of bio-derived components may range from approximately 30% to 70%, enabling a broad spectrum of medical applications [132].

The global market for bio-polyurethanes was valued at approximately USD 42 million in 2022, accounting for less than 0.1% of the total polyurethane market. Despite this relatively small share, the sector is expanding rapidly, driven by intensive research aimed at increasing the renewable content of polyurethane formulations. Enhancing the performance and reliability of bio-polyurethanes remains critical for meeting medical-industry requirements and supporting large-scale commercial investment [133].

An example of a biomedical polyurethane system involves the synthesis of a biocrystalline polyurethane from polycaprolactone, hexamethylene diisocyanate and water as a chain extender. Degradation studies conducted in simulated physiological media, such as phosphate-buffered saline, demonstrated favorable long-term thermal, mechanical and physical stability compared with polyurethanes extended with ethylene glycol. The use of water as a chain extender reduces production costs and offers an efficient route to medical-grade materials suitable for applications such as joint replacements [133].

Polyurethanes are widely applied in medical devices, including catheters, general-purpose tubing, hospital bedding, surgical drapes, wound dressings and injection-molded components. They are most commonly used in short-term implant applications, where polyurethane-based materials provide a favorable balance of cost efficiency, durability and mechanical strength [103].

## 4. Polyurethanes—Environmental Aspects

Polyurethane does not contain any chemicals that interfere with the endocrine system and does not contribute to changes in soil or water pH. In other words, it does not exert a negative environmental impact. It does not leach, contaminate water, or corrode healthy soil structures [134].

Polyurethane is also regarded as environmentally favorable due to the possibility of recycling and reuse. Depending on its chemical structure and form, polyurethane waste can be mechanically reprocessed or chemically recycled into secondary raw materials, enabling the recovery and reutilization of discarded products [134].

In recent years, however, the sustainability and recyclability of polyurethane foam have been questioned. The primary factor limiting sustainability and recyclability is the use of highly reactive and toxic isocyanates during production. Various types of catalysts and surfactants are also employed to manufacture PU foams with different properties. According to some estimates, approximately 30% of recovered polyurethane foam is still disposed of in landfills, representing a significant environmental challenge for the construction sector, as this material is not readily biodegradable. At present, only about one-third of polyurethane foams are effectively recycled [134].

The main benefits of using biomass in the production of both polyols and fillers include reduced reliance on fossil resources and a lower environmental footprint. For rigid polyurethane foams include a substantial reduction in the consumption of petrochemicals, the creation of more environmentally friendly materials, and improved performance of the final foams. Biomass used in PU foam production generally has negligible economic value. This is because it typically consists of agricultural or horticultural waste, which may have other potential industrial applications but is often composted due to its availability and the accessibility of relevant technologies in a given region [135].

### Aging and Degradation of Polyurethane Materials

Aging of polyurethane materials is a critical factor influencing recycling feasibility and process efficiency. During service life, polyurethanes are exposed to thermal, oxidative, hydrolytic and mechanical stresses, which induce chemical degradation of urethane and urea linkages, chain scission, changes in phase morphology and gradual loss of functional properties [136].

These aging-related transformations significantly affect the response of polyurethane waste to recycling processes. Progressive degradation increases structural heterogeneity, alters chemical functionality and reduces chain mobility, thereby limiting the effectiveness of mechanical recycling and complicating depolymerization during chemical recycling routes such as glycolysis or hydrolysis [47].

The impact of aging is particularly pronounced in long-lived applications such as building insulation, construction materials and automotive components, where polyurethane waste streams are characterized by advanced degradation, extensive crosslinking and high formulation variability [47,127]. As a consequence, recycling strategies must account for aging-related changes when selecting suitable mechanical, chemical or hybrid recycling approaches.

Despite its importance, aging is often addressed only implicitly in discussions of polyurethane recycling. Explicit consideration of aging-related degradation processes is therefore essential for developing realistic, scalable and sustainable end-of-life strategies for polyurethane materials [6,47,52].

## 5. Recycling of Polyurethanes

### 5.1. Mechanical Recycling

Mechanical recycling involves the physical processing of polyurethane (PU) waste to generate secondary materials without altering the chemical structure of the polymer. In this approach, PU waste is typically shredded and subsequently reused as a filler or reinforcing component in the production of new polyurethane products [37].

Primary and secondary recycling—corresponding to mechanical and physical recycling—represent the simplest and most established polyurethane recycling strategies. These methods are generally cost-effective and require relatively low capital investment; however, the mechanical and thermal performance of recycled PU materials is often inferior to that of virgin products, which limits their market value and application range [137].

In physical recycling, PU waste is converted into powders, flakes or granulates that are reintroduced into new materials through purely physical transformation processes. The main mechanical recycling techniques applied to polyurethane include:

1. Rebonding—Flexible polyurethane foam is shredded into small particles and bonded to produce products such as sports mats and carpet underlays.
2. Grinding or powdering—Finely ground PU waste is mixed with one of the original reactants, typically a polyol (up to approximately 30 wt%), to manufacture new polyurethane materials.
3. Compression molding—Powdered PU waste is processed under elevated temperature and pressure, allowing the production of components containing up to nearly 100% recycled material [138].

Although these methods are well suited for thermoplastic polymers, their effectiveness is limited for polyurethanes owing to their predominantly thermoset character. As a result, mechanical recycling has gradually shifted from early applications involving the reuse of PU scraps as cushioning fillers toward the development of more advanced recycling strategies. In particular, chemical recycling enables the depolymerization of polyurethane waste into low-molecular-weight compounds or monomers (tertiary recycling), which may serve as feedstocks for petrochemical processes or as precursors for new chemical synthesis, thereby enabling the production of higher-value materials [40].

One of the key challenges in mechanical recycling is maintaining a uniform foam cell structure at higher recycled-content levels, as structural deterioration can negatively affect thermal insulation performance. A potential strategy to address this limitation involves incorporating recycled insulating materials with superior insulation capacity into virgin PU matrices. In this context, phenolic foams have been proposed as promising candidates. These thermosetting materials are synthesized via the exothermic condensation reaction between phenol and formaldehyde and exhibit favorable insulating properties [139].

From an industrial perspective, mechanical recycling remains an economically viable approach for polyurethane waste management, as it avoids chemical degradation and complex processing steps. The principal mechanical recycling routes include binder-assisted pressing, binder-free pressing and regrinding. In binder-assisted pressing—the most widely applied industrial method—PU waste is shredded, mixed with a binder (typically an isocyanate) in the presence of steam, and compressed to achieve the desired density. Binder-free pressing relies solely on heat and pressure to bond PU waste particles. In this case, the thermoplastic behavior of soft segments at temperatures between approximately 150 and 220 °C enables thermal softening and interdiffusion. In the regrinding approach, PU waste is milled into powder and reintroduced as a filler in new foam formulations [43].

At present, rebonding is the most extensively used industrial mechanical recycling technology for flexible polyurethane foams and is applied almost exclusively for the recycling of flexible foam waste prior to final end use [138].

#### 5.1.1. Grinding and Reprocessing

In addition to rebonding and reprocessing routes, the direct use of polyurethane waste in flake form as a low-cost filler material in construction applications, such as building blocks for sidewalks or walkways, has also been considered. Such an approach may offer a simple valorization pathway, particularly where high mechanical performance is not required.

#### 5.1.2. Rebonding

In the rebonding process, flexible polyurethane foam waste is shredded into particles with typical dimensions of approximately 1 cm and mixed with monomers or prepolymers used in the production of flexible PU foams. These systems are commonly based on methylene diphenyl diisocyanate (MDI) and polyester- or polyether-based polyols. A major limitation of rebonding technology is the minimum achievable density of the resulting materials, which generally exceeds 60 kg m^−3^, restricting applications where low-density foams are required [139].

**Figure 27:**
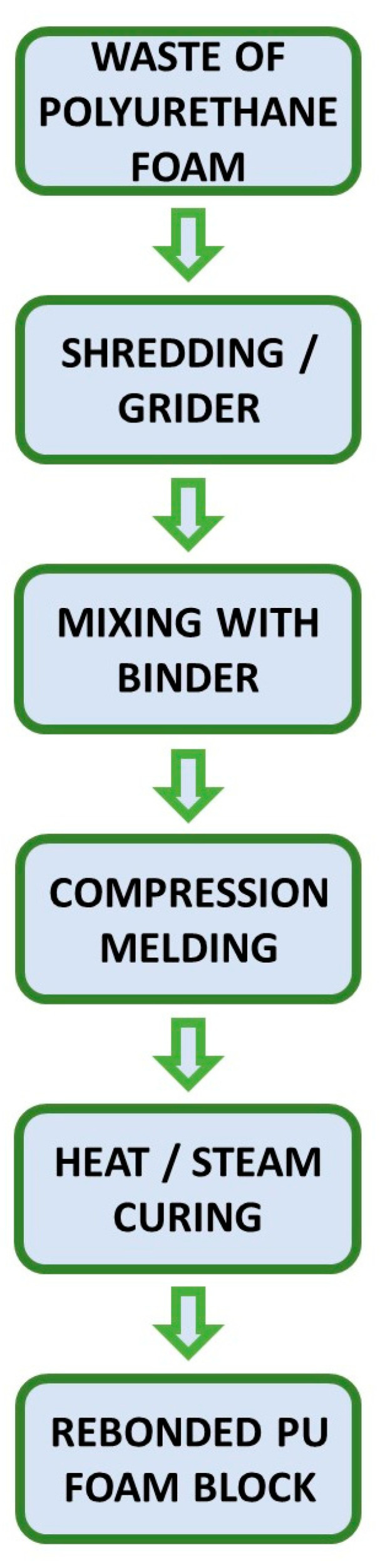
Schematic representation of the mechanical rebonding process for polyurethane foam waste. Illustration created by the Authors.

The mechanical rebonding process used for the recycling of polyurethane foam waste is schematically illustrated in Figure 27.

#### 5.1.3. Adhesive Pressing

Adhesive pressing is a mechanical recycling method in which polyurethane granules are coated with a binder and subsequently cured under elevated temperature and pressure [10]. In this process, PU granules are combined with an adhesive—most commonly a polymeric isocyanate—and processed at pressures of approximately 30–200 bar and temperatures in the range of 100–200 °C.

This technique enables the production of semi-finished and finished products such as automotive floor mats and tire covers. Adhesive pressing can be applied to a broad range of plastic waste streams, including mixed polymer waste, offering a relatively direct route to the manufacture of construction-grade polyurethane panels. Materials produced using this method typically exhibit high moisture resistance and good thermal insulation properties.

The principal limitations of adhesive pressing arise from the widespread presence of flame retardants in many polyurethane foam types, which significantly reduces the availability of suitable feedstock and necessitates careful waste segregation. In addition, the relatively high density of the resulting products restricts their applicability in markets requiring lightweight materials [140].

#### 5.1.4. Mechanical Processing Without Adhesives

Hot compression molding represents an adhesive-free mechanical recycling route for polyurethane waste. In this process, finely ground polyurethane foam particles are compacted under high pressure and elevated temperature. The method is primarily used for processing rigid PU foam waste generated in the automotive industry, such as bumper components.

The molded products obtained exhibit rigid, three-dimensional structures and can be used directly as functional components, including pump housings or engine covers. When glass fibers are incorporated during molding, the resulting composites are suitable for applications such as dashboard elements, door panels and related automotive parts.

In addition, materials produced by incorporating polyurethane waste as a filler in compression-molded resin systems—such as polyester-based composites—demonstrate improved flexibility and impact resistance compared with mineral-filled counterparts. Despite these advantages, the method is limited by difficulties associated with recycling painted components and by challenges related to producing sufficiently fine polyurethane particles required for high-quality molded products [138,139].

#### 5.1.5. Reaction Injection Molding (RIM)

Reaction injection molding (RIM) is a processing technique used for the manufacture of components such as electrical enclosures, computer housings and telecommunications equipment. The process involves mixing two low-viscosity liquid components—a polyol and an isocyanate—which are injected into a closed mold, where an in situ polymerization reaction forms polyurethane with the desired geometry.

Depending on the formulation and processing conditions, the resulting products may be solid or foamed. The system can also be reinforced with glass fibers (reinforced RIM, RRIM) or combined with structural composites (structural RIM, SRIM) to enhance mechanical performance.

RIM technology can be applied to mixed streams of polyurethane waste as well as to blends of PU with other plastics, typically thermoplastics. In addition, finely ground rigid polyurethane foam waste may be incorporated as a core or filler material, enabling partial reuse of recycled PU within new molded components [140].

**Figure 28:**
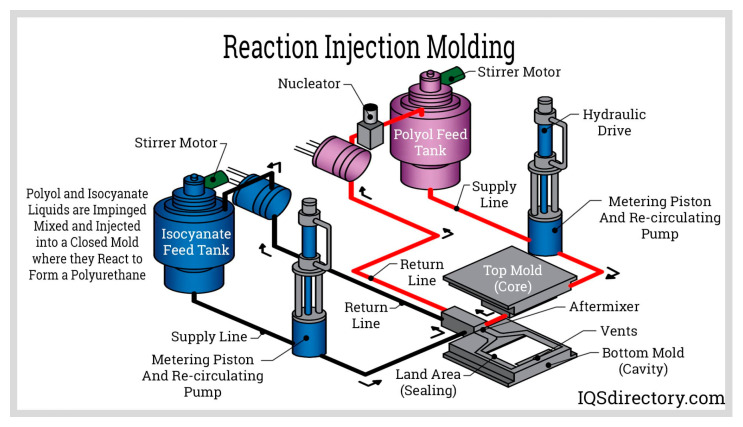
RIM molding process [140].

The reaction injection molding (RIM) process used for the manufacturing and recycling of polyurethane materials is schematically illustrated in Figure 28.

#### 5.1.6. Compression Molding

Compression molding enables the production of components containing up to 100% recycled polyurethane material and is applied mainly to polyurethanes originally manufactured by reaction injection molding (RIM). In this process, RIM and reinforced RIM (RRIM) parts are ground into fine particles and subsequently processed under elevated temperature and pressure to form dense, solid materials suitable for a range of automotive applications.

Recycling efforts in this area focus primarily on the manufacture of finishing components from reprocessed polyurethane and from PU recovered from end-of-life vehicles. In some cases, RIM-derived materials modified with glycol and compression-molded at approximately 195 °C have been reported to exhibit mechanical properties comparable to, or even exceeding, those of the original polyurethane materials.

Compression molding involves shaping polyurethane particles under conditions of high temperature and pressure sufficient to generate shear forces that promote particle bonding without the use of additional binders [30]. The process yields rigid, three-dimensional components such as pump and engine housings. It has been successfully applied to the recycling of RIM-manufactured automotive components, including covers and panels, although the recycling of painted parts remains a significant challenge. Typical recycled products include fenders and sports flooring, often produced in combination with rubber granulates.

Door panels and dashboard components can be manufactured using formulations containing approximately 6% RIM regrind combined with about 15% glass-fiber reinforcement. Recycling of structural reaction injection molding (SRIM) materials is particularly relevant when coarsely ground polyurethane waste is used. In this approach, recycled polyurethane—at levels of up to 30 wt%—is pressed between glass-fiber reinforcements pre-impregnated with a two-component polyurethane resin, yielding components with enhanced stiffness suitable for structural automotive applications [141].

### 5.2. Energy Recovery

Energy recovery is often regarded as the most appropriate management route for polyurethane waste streams for which material recycling is technically or economically unfeasible. This is particularly relevant for complex scrap materials containing polyurethane laminates bonded to wood, leather, textiles or other mixed substrates.

Flame-retarded polyurethane materials present additional challenges for recycling, further favoring energy recovery options. Combustion of polyurethane foam can reduce waste volume by approximately 99%, significantly decreasing landfill requirements while simultaneously destroying chlorofluorocarbons (CFCs) and other hazardous blowing agents contained within the foam structure [141].

### 5.3. Thermochemical Recycling

Thermochemical recycling encompasses processes in which polyurethane (PU) waste is decomposed at elevated temperatures to recover energy or generate secondary raw materials. In principle, depolymerisation can be applied to relatively pure streams of specific polyurethane types, enabling their breakdown into original or low-molecular-weight building blocks that may subsequently be repolymerised. However, because post-consumer polyurethane waste typically consists of complex and mixed material streams, thermochemical feedstock-recycling routes have been developed to handle such heterogeneous compositions.

Five principal thermochemical processing methods have been reported for polyurethane waste: pyrolysis, blast-furnace processing, gasification, hydrogenation and incineration [52]. A critical factor governing the efficiency and outcome of these processes is the thermal degradation behavior of polyurethane foams. The degradation mechanism strongly depends on the temperatures at which specific chemical bonds within the polymer backbone dissociate, which in turn are dictated by the chemical structure of the polyurethane chain.

Polyurethane materials are composed primarily of isocyanates, polyols and chain extenders, and variations in their composition lead to significant differences in thermal decomposition pathways. Consequently, reported results for thermochemical recycling vary widely depending on the nature of the polyurethane waste stream investigated [52].

Thermochemical routes such as pyrolysis, gasification and hydrogenolysis typically require high temperatures (up to approximately 800 °C) and elevated pressures (up to around 80 bar). Under these severe conditions, most bonds within the polyurethane structure are cleaved, yielding gaseous products such as methane (CH_4_), carbon monoxide (CO), hydrogen (H_2_) and nitrogen-containing species. More thermally resistant organic fragments may be stabilized into petroleum-like products in the presence of hydrogen, while inorganic residues form ash [142].

In practice, the high calorific value of polyurethane waste is often exploited for energy recovery rather than for the production of new polyurethane materials. However, thermochemical recycling processes are characterized by high energy demand, poorly controlled product distributions and the generation of potentially toxic gaseous by-products, particularly nitrogen-containing compounds. These drawbacks significantly limit the technical and economic feasibility of large-scale gasification and pyrolysis for polyurethane recycling.

Hydrogenolysis has attracted increasing attention in recent years [54,55]; nevertheless, the requirement for high hydrogen pressures (typically exceeding 30 bar) and the associated cost and safety concerns related to hydrogen handling pose major challenges for industrial implementation.

In contrast, chemical recycling routes aimed at selective depolymerisation offer several advantages, including relatively short reaction times, milder operating conditions, targeted production of repolyols and comparatively low reagent costs. As a result, current research efforts focus predominantly on chemical recycling methods that yield repolyols, such as hydrolysis, glycolysis, acidolysis and aminolysis. Comparative studies evaluate the properties of repolyols obtained via these approaches and identify existing challenges, knowledge gaps and future opportunities for sustainable polyurethane recycling. Both experimental and theoretical investigations are employed to elucidate the underlying mechanisms governing polyurethane depolymerisation into reusable polyol fractions [142].

#### 5.3.1. Pyrolysis

Pyrolysis involves the thermal decomposition of polyurethane waste in an oxygen-free or oxygen-deficient environment. Under these conditions, the polymer macromolecules undergo cleavage reactions, producing a mixture of gaseous, liquid and solid products. A solid carbonaceous residue (char) is typically formed as an undesired by-product, although its yield is generally limited [141].

Most studies on polyurethane pyrolysis are conducted at laboratory scale using thermogravimetric analysis (TGA) under a nitrogen atmosphere. In TGA experiments, the mass loss of a polyurethane sample is monitored as a function of temperature at a controlled heating rate (K·min^−1^), providing insight into degradation stages and thermal stability. In some investigations, TGA is coupled with differential scanning calorimetry (DSC) to simultaneously measure heat flow associated with decomposition reactions.

**Figure 29:**
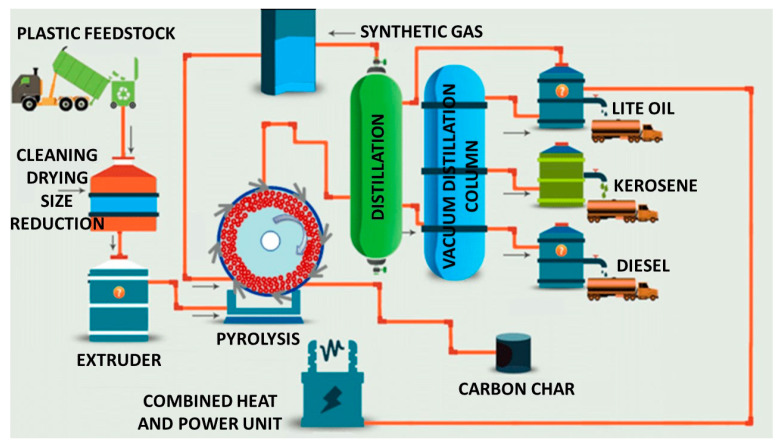
Schematic process of polyurethane pyrolysis.

Numerous pyrolysis studies have been reported for polyurethane materials with varying chemical compositions and industrial applications, highlighting the strong dependence of degradation behavior and product distribution on the structure of the polyurethane waste and its formulation additives [143]. Schematic process of polyurethane pyrolysis was presented in the Figure 29.

#### 5.3.2. Hydrogenation

Hydrogenation is a thermochemical recycling process related to pyrolysis, in which polyurethane waste is treated under a hydrogen atmosphere rather than an inert gas. Conventional hydrogenation is typically conducted at temperatures around 450 °C and under high hydrogen pressures ranging from 150 to 300 bar, producing a mixture of gaseous and liquid hydrocarbons commonly referred to as syncrude [36]. The use of catalysts can significantly enhance reaction rates and influence the selectivity of the resulting products.

Gausas et al. [144] demonstrated the catalytic hydrogenation of both rigid and flexible polyurethane foams using commercially available iPrMACHO-type complexes [37]. Among the investigated iridium-, manganese- and ruthenium-based iPrMACHO catalysts—which all exhibited high and comparable conversion efficiencies—the iridium-based iPrMACHO complex produced the highest yield of aniline. In this study, hydrogenation was performed at a hydrogen pressure of 30 bar and temperatures between 150 and 180 °C, yielding recoverable polyol fractions and isolated aniline suitable for use as precursors in the synthesis of new polyurethane materials. Related work by Zhou et al. [145] demonstrated that hydrogenation strategies can also be applied to polymeric feedstocks beyond polyurethane, such as polyamide 6,6.

**Figure 30:**
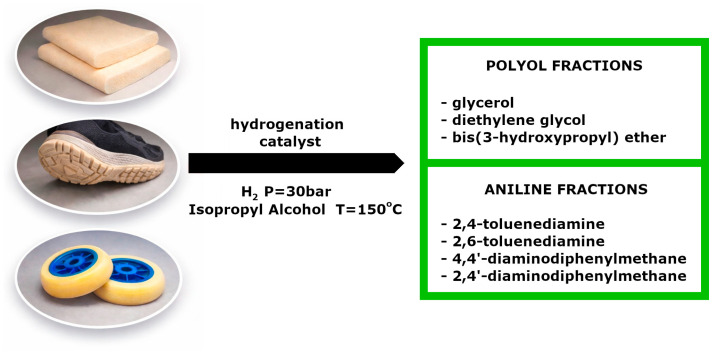
Hydrogenation of PU waste [28].

Despite their potential environmental and economic advantages, thermochemical recycling approaches face several limitations. Although valuable chemical feedstocks—including CH_4_, H_2_ and CO_2_—can be recovered, polyurethane waste streams are often inadequately separated and remain mixed with municipal waste, limiting their suitability for selective thermochemical valorisation. In addition, the generation of toxic gaseous by-products and the requirement for high pressures and temperatures constrain large-scale industrial implementation. While hydrogenation and related technologies enable energy recovery, they are also associated with substantial greenhouse gas emissions, which currently reduce their competitiveness relative to alternative plastic-waste valorisation routes. Further reductions in CO_2_ emissions are therefore required to improve the sustainability of these processes [146]. Schematic route of hydrogenation of polyurethane waste was presented in the Figure 30.

### 5.4. Chemical Recycling of Polyurethanes

#### 5.4.1. Glycolysis

Glycolysis is one of the most developed chemical recycling methods for polyurethane waste and, alongside acidolysis, the only approach that has been implemented at pilot scale. In this process, polyurethane depolymerization is initiated by the addition of a glycol—most commonly ethylene glycol or diethylene glycol—via a transcarbonylation reaction that cleaves urethane linkages and yields hydroxyl-terminated products [147].

The primary objective of polyurethane glycolysis is the recovery of repolyols that can be reused in the synthesis of new polyurethane materials. The process converts a heterogeneous polyurethane waste stream into a homogeneous liquid phase containing low-molecular-weight polyols, making glycolysis one of the most promising chemical recycling routes for crosslinked and thermoset polyurethane systems.

**Figure 31:**
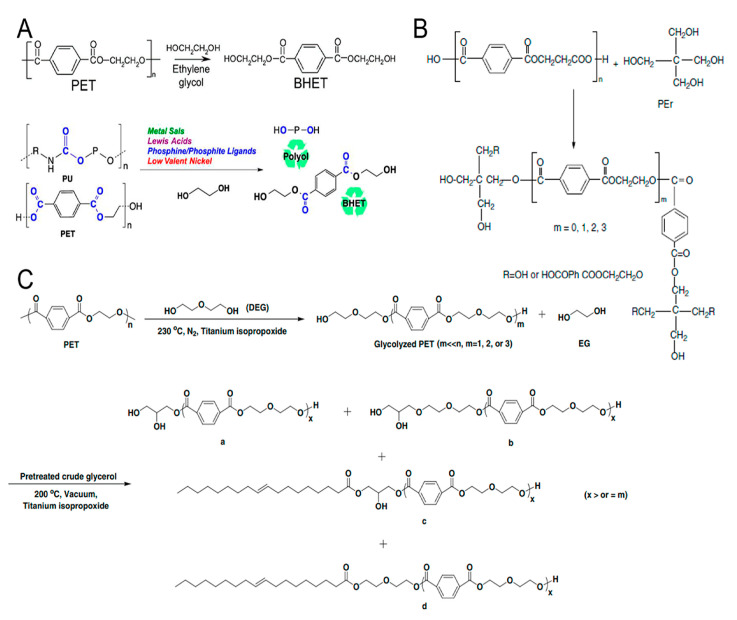
PET glycolysis. (**A**) Recovery of polyols from polyurethane waste and BHET from PET waste via transesterification using ethylene glycol as the transesterification agent, in the presence of metal salts, Lewis acids, phosphine/phosphite–metal complexes, or low-valent Ni(COD)_2_ as catalytic precursors [152]. (**B**) Depolymerization of PET through glycolysis using pentaerythritol as the transesterification reagent. (**C**) Polyols synthesized by glycolysis of polyester waste using diethylene glycol and crude glycerol, yielding mixtures of oligomers, diols, and triols.

Glycolysis is also widely established as an industrial recycling method for polyethylene terephthalate (PET), where it has been commercialized by numerous companies, including DuPont, Dow Chemical, Goodyear, Shell, Eastman and others. In PET glycolysis, ester bonds in the polymer backbone are cleaved through transesterification with excess ethylene glycol, producing bis(hydroxyethyl) terephthalate (BHET), dimers and oligomers, typically in the presence of metal acetate catalysts (Figure 31). Although PET glycolysis differs mechanistically from polyurethane recycling, PET-derived glycolysis products are frequently used as polyol precursors in polyurethane formulations.

Numerous studies have reported the synthesis of oligomeric polyols from waste PET bottles via glycolysis and their subsequent application in polyurethane systems. Atta et al. [148] recycled PET waste into oligomeric polyols using trimethylolpropane or pentaerythritol, which were successfully employed in the production of polyurethane foams. Degradation of PET with ethylene glycol leads to the formation of hydroxyl-functional oligomers suitable for further polymer synthesis. Luo et al. [149] reported PET degradation using diethylene glycol, yielding low-molecular-weight diols that were subsequently reacted with crude glycerol to produce polyols and polyurethane foams. Scremin et al. [150] described PET glycolysis with diethylene glycol, where the resulting PET-based polyol was applied in one-step polyurethane adhesive formulations.

The oligomeric products obtained through glycolysis are predominantly terminated with hydroxyl groups, rendering them suitable as raw materials for the synthesis of new polyurethane systems. Ramin Shamsi et al. [151] reported an environmentally friendly approach in which carbon nanotubes were incorporated into polyurethane matrices derived from PET-based polyols, resulting in nanocomposites with enhanced mechanical properties. In addition, the swelling behavior of crosslinked polyurethanes in polar and nonpolar solvents was investigated, providing insight into diffusion and adsorption mechanisms in recycled polyurethane materials.

Overall, glycolysis enables the conversion of polymer waste into value-added polyols that can be reintroduced into polyurethane synthesis, supporting circular-economy strategies and reducing reliance on virgin petrochemical feedstocks.

#### 5.4.2. Carbamate Aminolysis in Polyurethanes—Linear Carbonates

**Figure 32:**
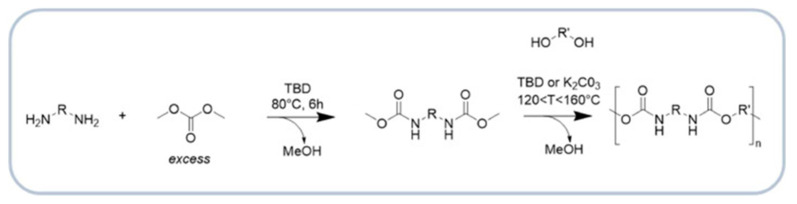
Carbamate aminolysis of linear carbonates [37].

To eliminate the use of isocyanates, carbamate linkages can be formed via aminolysis of alkyl carbonates, as illustrated in Figure 32. Dimethyl carbonate is most frequently employed due to its liquid state and the benign nature of the by-product formed during aminolysis, namely methanol, which can be readily removed from the reaction medium. The reaction is typically carried out using an excess of carbonate to ensure sufficient solubility of the amine and to drive the equilibrium toward carbamate formation.

Early studies relied on metal-based catalysts such as scandium(III) triflate or lead(II) nitrate; however, these systems are increasingly being replaced by organic catalysts, including 1,5,7-triazabicyclo[4.4.0]dec-5-ene (TBD) and tetrabutylammonium bromide [63]. Notably, aminolysis of linear carbonates can also proceed under solvent-free and catalyst-free conditions, which significantly improves the environmental profile of the process and aligns with green-chemistry principles [153].

This approach enables the synthesis of dicarbamate monomers, which can subsequently undergo transurethanisation in the presence of diols to produce thermoplastic polyurethanes [154].

**Figure 33:**
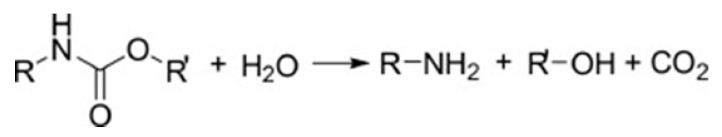
Carbamate aminolysis of dimethyl carbonate leading to the formation of dicarbamate intermediates subsequently used in the synthesis of thermoplastic polyurethanes [155].

A major limitation of this method is the undesired formation of primary aromatic diamines, such as methylenedianiline (MDA) or 2,4-toluenediamine (TDA) [68]. During the process, the aqueous upper phase contains the amines, while the lower phase consists of the recovered polyol. Polyurethane hydrolysis is typically carried out using steam in a high-pressure reactor at elevated temperature. The general hydrolysis mechanism is illustrated in Figure 33 [155].

The principal limitation of this approach is the undesired formation of primary aromatic diamines, such as methylenedianiline (MDA) or 2,4-toluenediamine (TDA) [69]. These by-products are toxic and adversely affect the quality of the recovered polyols, as increasing diamine content leads to a significant rise in mixture viscosity. During processing, the amines are concentrated in the aqueous phase, whereas the recovered polyols form a separate organic phase. The aromatic diamines can be reused via phosgenation to produce new isocyanates, while the recovered polyols may be incorporated into foam formulations by blending with virgin polyols at levels of up to 20 wt.%.

Despite these possibilities, this recycling route suffers from major drawbacks, including high energy demand, harsh reaction conditions, and limited economic viability, which restrict its suitability for large-scale or profitable recycling applications [70].

Duval et al. [156] applied this strategy to synthesize non-isocyanate polyurethanes from renewable diamines and diols, such as 1,6-hexanediamine or Priamine, combined with butanediol or diols derived from methyl ricinoleate. The resulting materials exhibited number-average molar masses in the range of 3000–14,000 g·mol^−1^. Among the catalysts investigated for the transurethanisation step, potassium carbonate (K_2_CO_3_) proved more effective than TBD, enabling operation at higher temperatures and yielding non-isocyanate polyurethanes with increased molar masses.

#### 5.4.3. Hydrolysis

Hydrolysis is one of the earliest chemical recycling methods applied to polyurethane foams. In this process, polyurethane waste is treated with superheated steam at temperatures of approximately 200 °C and pressures around 15 atm, leading to depolymerisation of urethane linkages and the formation of a biphasic liquid system consisting of recovered polyols and diamines [68].

In the case of polyethylene terephthalate, hydrolysis proceeds via nucleophilic attack of water molecules on ester bonds under controlled temperature and pressure conditions, yielding terephthalic acid (TPA) and ethylene glycol (EG). This reaction represents the reverse of the polycondensation process used for PET synthesis, whereby the introduction of water or steam induces chain cleavage and depolymerisation [157].

In recent years, several alternative hydrolysis strategies have been proposed. Vjekoslav et al. [158] reported a quantitative and solvent-free mechanochemical route for PET hydrolysis to TPA using ball milling at ambient temperature and pressure. Kang et al. developed a microwave-assisted hydrolysis process employing ZSM-5 zeolite as a catalyst under inert conditions, achieving efficient PET depolymerisation.

**Figure 34:**
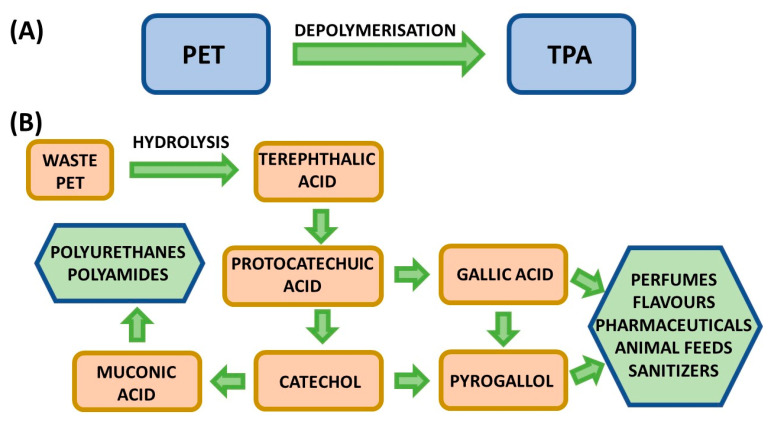
Hydrolysis of polyethylene terephthalate (PET) to terephthalic acid (TPA). (**A**) Alkaline hydrolysis of solid PET to TPA via steam-assisted aging under varying relative humidity levels and solvent-vapor conditions [158]. (**B**) General scheme illustrating the upgrading and utilization of a biorefinery platform for PET waste valorization [160].

**Figure 35:**
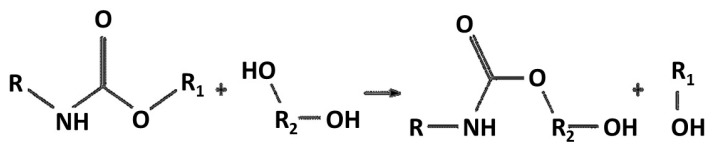
Hydrolysis reaction of polyurethane [161].

Terephthalic acid is an important chemical intermediate used in the synthesis of a wide range of products, including biodegradable plastics, and can be repolymerised with ethylene glycol to regenerate PET, enabling true closed-loop recycling. Moreover, emerging valorisation pathways aim to convert PET hydrolysis products into higher-value compounds. In such approaches, waste PET is first depolymerised into TPA and EG, followed by microbially catalyzed transformation into aromatic compounds and functional derivatives with potential applications in pharmaceuticals, cosmetics, disinfectants, animal feed, and bio-based monomers [159]. Schematic route of hydrolysis of polyethylene terephthalate (PET) to terephthalic acid (TPA) was presented in the Figure 34. Schematic reaction of hydrolysis of polyurethane was presented in the Figure 35.

#### 5.4.4. Hydro-Glycolysis

Hydro-glycolysis of polyurethane, often referred to as soft glycolysis, has attracted increasing interest due to its relatively mild reaction conditions. This process represents an integrated recycling strategy that combines hydrolysis and glycolysis in a single step. Depolymerisation is carried out using a mixture of water and an alcohol as degrading agents, typically at approximately 100 °C for reaction times of up to 24 h [162]. It has been reported that the presence of small amounts of water during glycolysis can improve the purity of the recovered polyols. Hydro-glycolysis has also been successfully investigated under atmospheric pressure using microwave-assisted reactors, offering further potential for process intensification [163].

##### Acidolysis

As reported by Sołtysiński et al. [164], acidolysis offers the important advantage of producing recovered polyols (RP) free of amines, including carcinogenic aromatic diamines such as toluenediamine (TDA) and methylenedianiline (MDA). Owing to this benefit, acidolysis-based recycling technologies have attracted considerable interest from manufacturers of both rigid and flexible polyurethane foams. Industrial stakeholders such as Rampf Group [162] and H&S Anlagentechnik [165] are actively developing acidolysis processes, underlining the growing relevance of this recycling route. Notably, the technology developed by H&S Anlagentechnik has already been implemented at industrial scale at the Dendro foam-processing facility in Poland [165].

In parallel, the PUReSmart consortium represents a coordinated effort across the entire polyurethane value chain, bringing together nine partners from six countries [165]. The consortium aims to develop innovative technologies—acidolysis among them—to enable fully circular polyurethane materials. Current research focuses on elucidating acidolysis degradation mechanisms, assessing the influence of reaction parameters on RP properties, and extending the applicability of this process to different polyurethane waste streams, as described in previous studies [166].

**Figure 36:**
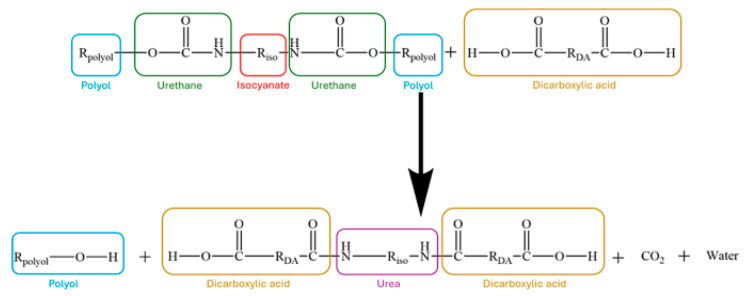
Polyurethane acidolysis [130,168].

Acidolysis can also be applied as part of a multi-step recycling strategy. He et al. [167]. proposed a sequential degradation approach in which polyurethane foam waste is first subjected to ammonolysis, followed by depolymerisation via acidolysis, as illustrated in Figure 36. The recovered polyols obtained in this process were used directly, without purification, as partial replacements for conventional polyols at loadings of up to 30 wt% in flexible foam formulations

Acidolysis requires the presence of a depolymerisation agent (DA), which reacts with carbamate groups within the polyurethane structure, leading to cleavage of the polymer chains and formation of polyols, oligomeric fragments, and low-molecular-weight compounds.

The acidolysis process leads to the formation of degradation products, as schematically illustrated in Figure 36.

**Figure 37:**
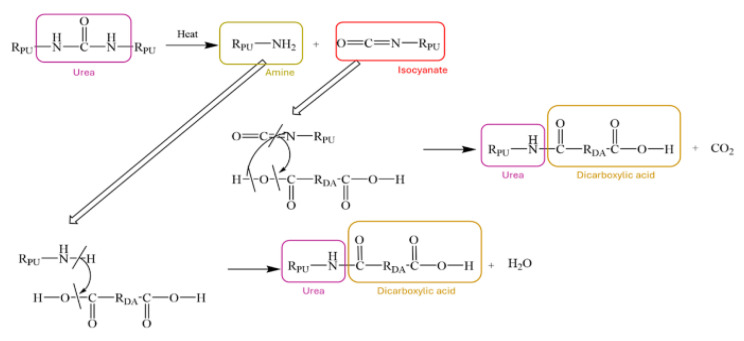
Thermal degradation of urea groups followed by reaction with DA [130,168].

The thermal degradation of urea groups, followed by their reaction with dicarboxylic acids, is schematically illustrated in Figure 37.

Where R_polyol_ denotes the conventional polyol backbone, R_iso_ corresponds to the isocyanate-derived segment, and R_DA represents the chain originating from the depolymerisation agent (DA).

In addition to the use of carboxylic acids as DAs, as described in patent DE 102013106364 A1 [127], dicarboxylic anhydrides have also been identified as effective alternatives. Moreover, several patents report the applicability of other organic acids as depolymerisation agents, including saturated and unsaturated fatty acids as well as mono- and polyfunctional carboxylic acids [164].

Apart from D_A_-induced depolymerisation of polyurethane chains, urethane and urea groups may undergo thermal degradation at temperatures close to 200 °C, resulting in their dissociation into amines and isocyanates [161]. These reactive species can subsequently participate in secondary reactions with the depolymerisation agent, leading to the formation of acid-terminated products accompanied by the release of water, as schematically illustrated in Figure 36 [168].

As a consequence, depending on the applied recycling conditions, recovery of both the polyol and the isocyanate-derived fraction may be achieved. It should be noted, however, that under certain conditions the isocyanate can undergo conversion into diamines, which—as discussed previously—is generally undesirable.

**Figure 38:**
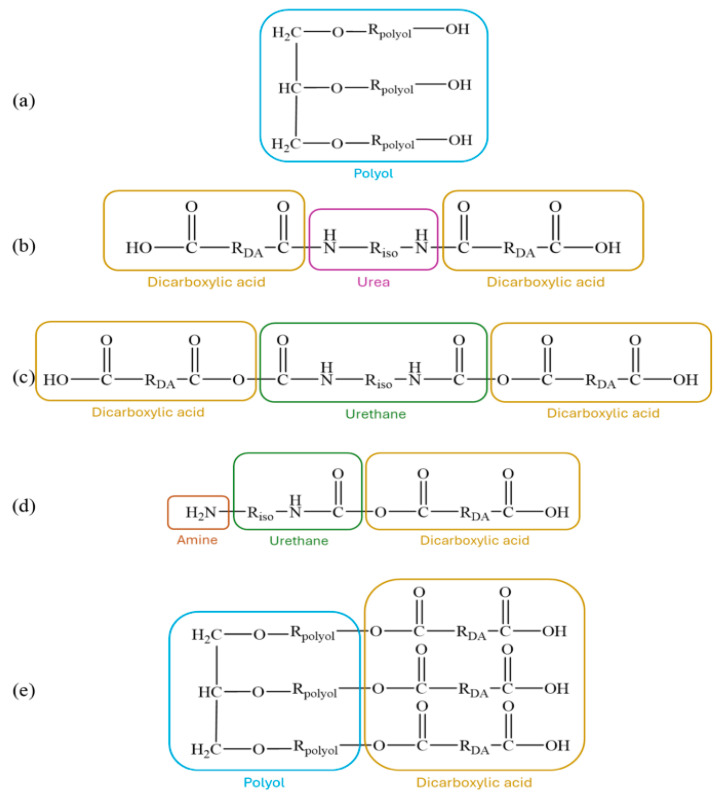
Identification of the most probable chemical environments present in the recovered polyols (RP) during depolymerisation of the polyurethane network: (**a**) structural formula of polyol originally used in the synthesis of the polyurethane foam; (**b**,**c**,**d**) structural formula formed through reactions of hydroxyl- or amine-terminated fragments with the depolymerisation agent dicarboxylic acid; (**e**) formation of polyester segments by reaction of a polyol with a dicarboxylic acid depolymerizing agent [168].

Polyurethane degradation is inherently complex and can result in the formation of a wide range of chemical species. Godinho et al. investigated the depolymerisation of polyurethane foams via acidolysis using succinic and phthalic acids as depolymerisation agents. Analysis of the molecular-weight distribution of the recovered polyols (RP) revealed that the dominant molecular species closely matched the molecular size of the conventional polyol (Figure 38a) originally used in the synthesis of the polyurethane foam. This finding strongly indicates that the primary component of the RP corresponds structurally to the initial polyol employed in foam production.

In addition to the main polyol fraction, lower-molecular-weight species were detected in the RP. These compounds can be attributed to the structures illustrated in Figure 38b–d and are likely formed through reactions of hydroxyl- or amine-terminated fragments with the depolymerisation agent, leading to various side products, as discussed earlier. Conversely, the presence of higher-molecular-weight fractions is indicative of incomplete depolymerisation of the polyurethane chains [164,166].

Product characterization was performed using Fourier-transform infrared (FTIR) spectroscopy and ^13^C nuclear magnetic resonance (NMR) analysis. These techniques revealed characteristic stretching vibrations associated with ester carbonyl (C=O) groups, confirming that the depolymerisation agent promotes the formation of hydroxyl functionalities. These O–H groups may subsequently participate in secondary polymerisation reactions with acidic groups, resulting in the formation of polyester segments, as schematically illustrated in Figure 38e [168].

**Figure 39:**
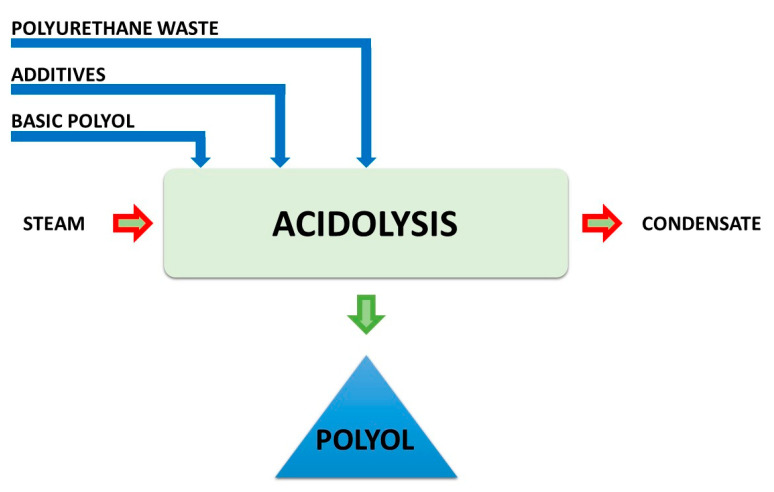
Process sequence and duration of acidolysis using the H&S method [170].

During the final stage of the reaction, water is generated as a by-product (Figure 38). Reported data indicate that approximately 85.4% of the total water is released within the first few hours of the process, reflecting the rapid progression of the initial depolymerisation steps [169]. As documented by H&S Anlagentechnik and illustrated in Figure 39, the process sequence for PU recycling via acidolysis closely resembles that of other chemical recycling methods [170].

### 5.5. Biological Degradation of Polyurethanes

Biodegradation refers to the breakdown of organic compounds by living organisms or their enzymes. This process leads to shortening of polymer chains and removal of specific structural components, resulting in a reduction in molecular weight and, under favorable conditions, potentially complete mineralization of the material. As biodegradation does not require high temperatures or aggressive chemical reagents, it is generally regarded as more environmentally benign than conventional chemical recycling routes and can be applied to post-consumer polyurethane waste. Three principal biological pathways have been identified for polyurethane degradation: bacterial, fungal, and enzymatic processes.

Polyester-based polyurethanes are markedly more susceptible to biodegradation than their polyether-based counterparts. Nevertheless, several studies employing fungal strains have reported promising results even for polyether polyurethanes. In such cases, degradation may be facilitated by the mechanical action of fungal hyphae penetrating the porous structure of the material, leading to physical damage and crack formation. By contrast, microorganisms are more commonly investigated for the degradation of polyurethane coatings, which can be attributed to the ability of bacteria to form stable biofilms on smooth polymer surfaces [171].

Fungi are recognized as key contributors to polyurethane degradation under laboratory conditions, and the majority of studies focus on soil-derived microorganisms involved in this process [172]. This emphasis may partly result from abiotic effects associated with fungal growth, such as pore enlargement and cracking caused by hyphal penetration. However, the precise degradation mechanisms and the relative contributions of biotic and abiotic factors remain insufficiently understood. Importantly, all major classes of polyurethanes—including coatings, foams, polyether- and polyester-based systems, and thermoplastic polyurethanes—have been shown to undergo biodegradation by selected bacterial and fungal strains [171].

In recent years, increasing attention has been directed toward enzymatic recycling strategies for end-of-life polyurethane management [34]. While chemical recycling can recover certain compounds suitable for reuse in polyurethane synthesis, the wide structural diversity of polyurethane waste and the variable quality of recovered polyols and amines significantly limit industrial applicability. Enzymatic approaches, in contrast, offer greater selectivity by targeting specific chemical bonds, enabling more controlled recovery of valuable building blocks from polyurethane waste. Enzymatic degradation involves the action of microorganisms or isolated enzymes that cleave the polymer matrix into smaller fragments. Microbial communities may colonize polyurethane surfaces, form biofilms, and secrete depolymerizing enzymes. The organisms involved in polyurethane biodeterioration are highly diverse and include bacteria, fungi, protozoa, algae, and lichen communities [173].

A major advantage of enzymatic degradation over biodegradation by whole microorganisms lies in the ability to control which bonds are cleaved, resulting in more well-defined degradation products that may be recycled or reused as precursors for new materials. The principal limitation of this approach is the substantial diversity of polyurethane formulations, which necessitates tailored treatment strategies for different polyurethane types [171].

**Figure 40:**
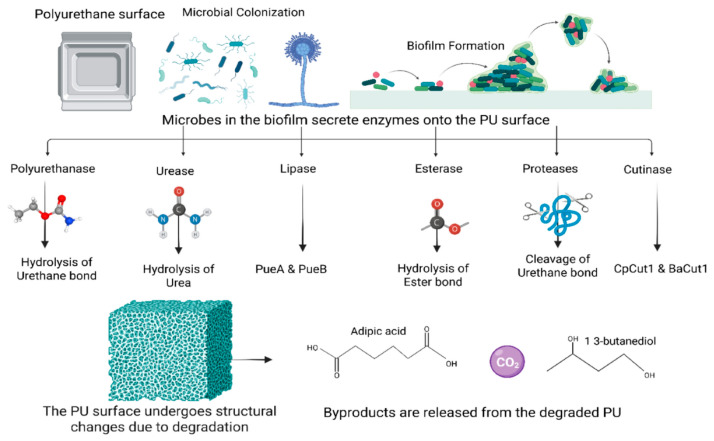
Stages of microbiological degradation of polyurethane [173].

The hydrolysis of ester linkages in polyester polyol segments by esterases accounts for many successful biodegradation results reported in the literature. Some studies suggest that esterases may also hydrolyze urethane bonds, forming alcohol-terminated chains and carbamic acid intermediates; however, due to the inherent instability of carbamic acid, rapid decomposition into amines and carbon dioxide is considered the more likely pathway. Moreover, because most biodegradation studies employ polyester-based polyurethanes, it is often difficult to distinguish between ester bond hydrolysis and direct urethane bond cleavage. Similar limitations apply to studies investigating urethane degradation by ureases, although these observations remain relevant for polyurethanes containing urea linkages. Such urea groups may be present in soft segments or formed during water-blown foaming reactions. Notably, urea bonds incorporated into long, flexible chains are significantly more susceptible to biological degradation. The formation of alcohols, amines, and carbon dioxide has been shown to further promote urethane bond cleavage by peptidases and amidases [173]. Stages of microbiological degradation of polyurethane was presented in the Figure 40.

Overall, polyurethane recycling technologies exhibit substantial differences in material recovery efficiency, process complexity and environmental impact. Mechanical recycling is widely implemented at the industrial scale but is limited by progressive deterioration of mechanical and thermal insulation properties. Chemical recycling routes enable higher-value material recovery through partial regeneration of polyols; however, they are typically associated with increased energy demand, solvent use and process complexity. Thermochemical approaches offer broad feedstock tolerance but involve high temperatures and substantial energy input. Emerging biological and hybrid recycling strategies operate under milder conditions and show potential environmental benefits, although their current technological readiness and scalability remain limited. A balanced assessment of polyurethane recycling technologies therefore requires simultaneous consideration of material performance, environmental impact and industrial feasibility.

### 5.6. Upcycling of Polyurethanes

In recent years, increasing attention has been directed toward upcycling strategies for polyurethane waste, which aim not only at material recovery but also at the generation of products with enhanced or tailored properties compared with the original materials. In contrast to conventional recycling approaches focused on restoring polymer building blocks, upcycling emphasizes value retention and functional improvement, thereby aligning more closely with circular-economy principles [174]. Chemical upcycling of polyurethanes typically involves controlled depolymerization processes and catalytic transformations that convert waste into valuable monomers and intermediates, which can be subsequently repurposed into high-value polymers or materials [175]. In addition to chemical routes, mechanical and thermochemical upcycling approaches have been explored where waste PU is converted into functional fillers or reinforcements for composites, contributing to improved mechanical performance and reduced reliance on virgin materials [176]. Emerging catalytic and hybrid strategies further expand the potential by enabling selective transformation pathways that reduce energy demand and improve sustainability metrics [177].

## 6. Conclusions

This review provides a comprehensive analysis of polyurethane recycling from the perspective of sustainable development, with particular emphasis on distinguishing established recycling technologies from emerging and innovative approaches. Although polyurethanes remain indispensable in modern industry, their extensive crosslinked architectures, formulation heterogeneity, and pronounced aging behavior continue to represent major obstacles to effective end-of-life management.

Mechanical recycling remains the most accessible and industrially implemented route; however, it is inherently constrained by progressive deterioration of mechanical, thermal, and insulation properties. Consequently, mechanically recycled polyurethane materials are typically downcycled and rarely reintroduced into high-performance applications. In contrast, chemical recycling methods—including glycolysis, hydrolysis, and aminolysis—offer higher material-recovery potential by enabling partial regeneration of polyols, although their effectiveness strongly depends on feedstock composition, contamination level, and aging state.

Aging of polyurethane materials emerges as a critical, yet frequently underestimated, factor governing recyclability. Chemical oxidation, hydrolytic degradation, chain scission, and changes in phase morphology significantly influence both process efficiency and the quality of recovered products. Addressing aging-induced degradation is therefore essential for the development of realistic, robust, and scalable recycling strategies.

Innovative approaches, such as catalytic depolymerization, bio-based and recycled polyols, non-isocyanate polyurethane (NIPU) chemistry, and hybrid chemical–biological recycling pathways, demonstrate considerable potential for improving resource efficiency and reducing environmental impact. Nevertheless, many of these strategies remain at an early stage of technological development and face challenges related to catalyst stability, economic viability, feedstock variability, and large-scale implementation.

From a sustainability perspective, the effectiveness of polyurethane recycling cannot be assessed solely on the basis of material-recovery yields. Qualitative indicators, including energy demand, process complexity, preservation of functional properties, compatibility with existing manufacturing infrastructure, and overall environmental footprint, must also be considered. No single recycling route currently fulfills all these criteria, underscoring the need for application-specific and hybrid solutions.

Overall, future progress in polyurethane recycling will require an integrated approach combining advances in polymer chemistry, aging-resistant material design, catalytic process development, and circular-economy-oriented product engineering. The insights presented in this review aim to support the development of more sustainable polyurethane systems and to guide future research toward recycling strategies that are not only technically feasible but also environmentally and economically viable.

## Figures and Tables

**Table 1:** Overview of bio-polyols, including raw materials, synthesis routes, key properties and representative applications [31,32,33,34,35,36,37].

Type of Raw Material	Synthesis Method	Characteristics of the Obtained Polyol	Physicochemical Properties	Thermal/Mechanical Properties	Main Application Areas
Vegetable oils (soybean, castor, palm)	Epoxidation followed by ring-opening with an alcohol or polyhydroxy compound	Moderate hydroxyl value, high functionality, adjustable viscosity	Hydroxyl value: 150–350 mg KOH/g; viscosity: 500–2500 mPa·s; acid value < 5 mg KOH g^−1^	Tg: −40 °C to −10 °C; good flexibility; moderate thermal stability	Flexible/semi-rigid polyurethane foams; coatings
Lignin and cellulose derivatives	Chemical depolymerization, reduction or etherification	Aromatic structures; high thermal stability; variable reactivity	Hydroxyl value: 300–600 mg KOH g^−1^; viscosity > 3000 mPa·s	Tg: 50–120 °C; high modulus; dimensional stability	Rigid foams; composites; insulation materials
Glycerol and sugar alcohols	Esterification or etherification with organic acids or anhydrides	Low molecular weight; high hydroxyl functionality; high reactivity	Hydroxyl value: 400–900 mg KOH g^−1^; viscosity < 1000 mPa·s	Tg: −30 °C to −5 °C; high flexibility; low thermal resistance	Adhesives; coatings; flexible foams
Polyurethane waste and PET polymers	Chemical glycolysis or transesterification	Polyols of varied functionality; secondary hydroxyl groups	Hydroxyl value: 200–500 mg KOH g^−1^; residual ester content	Tg: 10–70 °C; moderate mechanical strength	Polyurethane recycling; circular-economy applications
Fatty-acid esters and microbial oils	Hydroformylation and hydrogenation	Linear/branched polyols; controlled polarity and chain length	Hydroxyl value: 100–250 mg KOH g^−1^; viscosity 400–1500 mPa·s	Tg: −50 °C to 0 °C; flexibility; oxidative stability	Coatings; elastomers; bio-based lubricants

**Table 2:** Patents related to bio-isocyanates and sustainable synthesis methods [58].

Patent No.	Type	Description	Category
US9950996B2	Bio-MDI/bio-aromatic isocyanate	Method for producing bio-based aromatic isocyanates from renewable feedstocks.	Bio-isocyanate
CN113461894A	PU foam from bio-TDI	Production of bio-based TDI and polyurethane foams derived from it.	Bio-isocyanate
EP3819259A1	Sustainable isocyanate synthesis	Isocyanate production using RWGS, CO_2_ and renewable energy inputs.	Sustainable process
WO2014147142A1	Allophanates for coatings	Low-toxicity allophanate compositions for polyurethane coating systems.	Green chemistry
US20200345678A1	Bio-IPDI from fatty acids	Production of aliphatic isocyanates from vegetable-oil-derived fatty acids.	Bio-isocyanate
WO2019123456A1	Bio-HDI from biomass	Synthesis of hexamethylene diisocyanate using bio-derived diamines obtained from agricultural waste.	Bio-isocyanate

**Table 5:** Major Classes of UV Stabilizers Used in Polyurethane Systems [87,88].

Class	Function	Typical Use Level (%)
UV Absorbers (UVA)	Absorb harmful UV radiation, prevent yellowing and polymer degradation	0.2–1.0
HALS (Hindered Amine Light Stabilizers)	Neutralize free radicals, prevent surface degradation	0.1–1.0
Quenchers	Deactivate excited molecules, reduce photodegradation	0.1–0.5

**Table 6:** Major classes of solvents used in polyurethane coatings, adhesives, and processing operations, with typical examples [103].

Solvent Class	Examples	Typical Applications
Aromatic hydrocarbons	Toluene, Xylene	PU coatings, adhesives
Ketones	Acetone, Methyl ethyl ketone (MEK) Methyl isobutyl ketone (MIBK)	Viscosity control, cleaning
Esters	Ethyl acetate, Butyl acetate	High-quality PU coatings
Alcohols	Isopropanol, n-Butanol	Solvent blends, reactivity control
Glycol ethers	Propylene glycol monomethyl ether (PM) Dipropylene glycol monomethyl ether (DPM) Propylene glycol monomethyl ether acetate (PGMEA)	PU dispersions, coatings

## Data Availability

No new data were created or analyzed in this study. Data sharing is not applicable to this article.

## References

[1] Franz A.W., Buchholz S., Albach R.W., Schmid R. (2024). Towards Greener Polymers: Trends in the German Chemical Industry. Green Carbon.

[2] Kolluru S., Thakur A., Tamakuwala D., Kumar V.V., Ramakrishna S., Chandran S. (2024). Sustainable Recycling of Polymers: A Comprehensive Review. Polym. Bull..

[3] Porobic Katnic S., de Souza F.M., Gupta R.K. (2024). Recent Progress in Enzymatic Degradation and Recycling of Polyurethanes. Biochem. Eng. J..

[4] Rossignolo G., Malucelli G., Lorenzetti A. (2024). Recycling of Polyurethanes: Where We Are and Where We Are Going. Green Chem..

[5] Simonini L., Sorze A., Maddalena L., Carosio F., Dorigato A. (2024). Mechanical Reprocessing of Polyurethane and Phenolic Foams to Increase the Sustainability of Thermal Insulation Materials. Polym. Test..

[6] Kemona A., Piotrowska M. (2020). Polyurethane Recycling and Disposal: Methods and Prospects. Polymers.

[7] Gama N., Godinho B., Marques G., Silva R., Barros-Timmons A., Ferreira A. (2020). Recycling of Polyurethane Scraps via Acidolysis. Chem. Eng. J..

[8] Fonseca L.P., Duval A., Luna E., Ximenis M., De Meester S., Avérous L., Sardon H. (2023). Reducing the Carbon Footprint of Polyurethanes by Chemical and Biological Depolymerization: Fact or Fiction?. Curr. Opin. Green Sustain. Chem..

[9] Gupta R.K., Kahol P.K. (2021). Polyurethane Chemistry: Renewable Polyols and Isocyanates.

[10] Mehta P., Chidambaram S.P., Selwyn W.T., Borgohain A. Technical Comparisons of Foams Using Various Blowing Agent Blends. Proceedings of the Polyurethanes Technical Conference 2014.

[11] Madbouly S.A. (2023). Novel Recycling Processes for Thermoset Polyurethane Foams. Curr. Opin. Green Sustain. Chem..

[12] Silva A.L., Bordado J.C. (2004). Recent Developments in Polyurethane Catalysis: Catalytic Mechanisms Review. Catal. Rev..

[13] Zhu M., Ma Z., Liu L., Zhang J., Huo S., Song P. (2022). Recent Advances in Fire-Retardant Rigid Polyurethane Foam. J. Mater. Sci. Technol..

[14] Sanda H.A., Abubakar M.A., Abubakar A.M., Bashir M., Stojchevski M. (2024). Utilization of Neem Seed Oil as Surfactant in the Production of Flexible and Rigid Polyurethane Foams. Emerg. Technol. Eng. J..

[15] Ionescu M. (2005). Chemistry and Technology of Polyols for Polyurethanes.

[16] Domanska A., Boczkowska A. (2014). Biodegradable Polyurethanes from Crystalline Prepolymers. Polym. Degrad. Stab..

[17] Cregut M., Bedas M., Durand M.J., Thouand G. (2013). New insights into polyurethane biodegradation and realistic prospects for the development of a sustainable waste recycling process. Biotechnol. Adv..

[18] Akindoyo J.O., Beg M.H., Ghazali S., Islam M.R., Jeyaratnam N., Yuvaraj A.R. (2016). Polyurethane Types, Synthesis and Applications—A Review. RSC Adv..

[19] Chundawat T.S., Verma N., Vaya D. (2021). Development in Synthesis and Coating Applications of Polyurethane. J. Chil. Chem. Soc..

[20] Prociak A., Kurańska M., Malewska E. (2021). Porous polyurethane plastics synthetized using bio-polyols from renewable raw materials. Polimery.

[21] Thomas S., Rane A.V., Kanny K., Abitha V.K., Thomas M.G. (2018). Recycling of Polyurethane Foams ISBN: 978-0-323-51133-9 William Andrew, Applied Science Publishers. https://books.google.pl/books?hl=pl&lr=&id=8aRBDwAAQBAJ&oi=fnd&pg=PP1&dq=Recycling+of+Polyurethanes:+&ots=8e5_pV2UxK&sig=WsyWW0kbcpG3-dlPEeiVBJXgsxo&redir_esc=y#v=onepage&q=Recycling%20of%20Polyurethanes%3A&f=false.

[22] Szycher M. (2013). Szycher’s Handbook of Polyurethanes.

[23] Muscat A., de Olde E.M., de Boer I.J.M., Ripoll-Bosch R. (2020). The Battle for Biomass: A Systematic Review of Food–Feed–Fuel Competition. Glob. Food Secur..

[24] Hai T.A.P., de Backer L.J.S., Cosford N.D.P., Burkart M.D. (2021). Renewable Polyurethanes from Sustainable Biological Precursors. Biomacromolecules.

[25] Hai T.A.P., Neelakantan N., Tessman M., Sherman S.D., Griffin G., Pomeroy R., Burkart M.D. (2020). Flexible Polyurethanes, Renewable Fuels, and Flavorings from a Microalgae oil waste stream. Green Chem..

[26] Saalah S., Abdullah L.C., Aung M.M., Salleh M.Z., Awang Biak D.R., Basri M., Osman Al Edrus S.S. (2021). Chemical and Thermo-Mechanical Properties of Waterborne Polyurethane Dispersion derived from Jatropha Oil. Polymers.

[27] Lligadas G., Ronda J.C., Galia M., Cadiz V. (2013). Renewable Polymeric Materials from Vegetable Oils: A Perspective. Mater. Today.

[28] Patil C.K., Jung D.W., Jirimali H.D., Baik J.H., Gite V.V., Hong S.C. (2021). Nonedible Vegetable Oil-Based Polyols in Anticorrosive and Antimicrobial Polyurethane Coatings. Polymers.

[29] Sharmin E., Ashraf S.M., Ahmad S. (2007). Synthesis, characterization, antibacterial and corrosion protective properties of epoxies, epoxy-polyols and epoxy-polyurethane coatings from linseed and Pongamia glabra seed oils. Int. J. Biol. Macromol..

[30] Mouren A., Avérous L. (2023). Sustainable Cycloaliphatic Polyurethanes: From Synthesis to Applications. Chem. Soc. Rev..

[31] Llevot A., Grau E., Carlotti S., Grelier S., Cramail H. (2016). From Lignin-derived Aromatic Compounds to Novel Biobased Polymers. Macromol. Rapid Commun..

[32] Kaur R., Singh P., Tanwar S., Varshney G., Yadav S. (2022). Assessment of bio-based polyurethanes: Perspective on applications and bio-degradation. Macromol.

[33] Gallezot P. (2012). Conversion of biomass to selected chemical products. Chem. Soc. Rev..

[34] de Souza F.M., Kahol P.K., Gupta R.K. Biomass-Derived Polyurethanes for Sustainable Future. Advances in Green Synthesis: Avenues and Sustainability.

[35] Yang S.K., Mu Y.X., Li D., Li T., Lin H.L., Zhang A.P., Bian J., Chen D.Q., Yang K.C. (2025). Degradable Thermoplastic Polyurethane Achieved through Dynamic Imine Bond-Containing Biobased Chain Extenders Incorporating to Regulate Microstructures. ACS Appl. Polym. Mater..

[36] Santos M., Mariz M., Tiago I., Alarico S., Ferreira P. (2025). Bio-Based Polyurethane Foams: Feedstocks, Synthesis, and Applications. Biomolecules.

[37] Prociak A., Kurańska M., Cabulis U., Ryszkowska J., Leszczyńska M., Uram K., Kirpluks M. (2018). Effect of bio-polyols with different chemical structures on foaming of polyurethane systems and foam properties. Ind. Crop. Prod..

[38] Kurańska M., Prociak A., Cabulis U., Kirpluks M. (2015). Water-blown polyurethane-polyisocyanurate foams based on bio-polyols with wood fibers. Polimery.

[39] Amri M.R., Al-Edrus S.S.O., Guan C.T., Yasin F.M., Hua L.S. (2021). Jatropha Oil as a Substituent for Palm Oil in Biobased Polyurethane. Int. J. Polym. Sci..

[40] Sołtysiński M., Piszczek K., Romecki D., Narożniak S., Tomaszewska J., Skórczewska K. (2018). Conversion of polyurethane technological foam waste and post-consumer polyurethane mattresses into polyols—Industrial applications. Polimery.

[41] Onn M., Jalil M.J., Mohd Yusoff N.I.S., Edward E.B., Wahit M.U. (2024). A Comprehensive Review on Chemical Route to Convert Waste Cooking Oils to Renewable Polymeric Materials. Ind. Crop. Prod..

[42] Dworakowska S., Bogdal D., Prociak A. (2012). Microwave-Assisted Synthesis of Polyols from Rapeseed Oil and Properties of Flexible Polyurethane Foams. Polymers.

[43] Akdogan E., Erdem M. (2023). A comprehensive research of low-density bio-based rigid polyurethane foams from sugar beet pulp-based biopolyol: From closed-cell towards open-cell structure. Ind. Crop. Prod..

[44] Rajput B.S., Forman A., Halloran M.W., Phung Hai T.A., Scofield G.B., Burkart M.D. (2023). Variation of Aliphatic Diisocyanates in Bio-Based TPUs. Macromolecules.

[45] Petrović Z.S., Wan X., Bilić O., Zlatanić A., Hong J., Javni I., Degruson D. (2013). Polyols and Polyurethanes from Crude Algal Oils. J. Am. Oil Chem. Soc..

[46] Staroń A. (2023). Composite Materials Based on Waste Cooking Oil for Construction Applications. Buildings.

[47] Gama N., Ferreira A., Barros-Timmons A. (2018). Polyurethane Foams: Past, Present, and Future. Materials.

[48] Gaikwad M.S., Gite V.V., Mahulikar P.P., Hundiwale D.G., Yemul O.S. (2015). Eco-friendly polyurethane coatings from cottonseed and karanja oil. Prog. Org. Coat..

[49] American Chemistry Council Polyurethane Applications and Benefits. https://www.americanchemistry.com.

[50] Delavarde A., Savin G., Derkenne P., Boursier M., Morales-Cerrada R., Nottelet B. (2024). Sustainable polyurethanes: Toward new cutting-edge opportunities. Prog. Polym. Sci..

[51] Kausar A. (2018). Polyurethane Nanocomposite Coatings: State of the Art and Perspectives. Polym. Int..

[52] Camadanli S., Hisir A., Dural S. (2022). Synthesis and Performance of Moisture-Curable Solvent-Free Silane-Terminated Polyurethanes for Coating and Sealant Applications. J. Appl. Polym. Sci..

[53] Agrawal A., Kaur R., Walia R.S. (2017). PU foam derived from renewable sources: Perspective on properties enhancement: An overview. Eur. Polym. J..

[54] National Institute for Occupational Safety and Health (NIOSH) Safer Choice Program. https://www.epa.gov/saferchoice.

[55] Stachak P., Łukaszewska I., Hebda E., Pielichowski K. (2021). Recent Advances in Fabrication of Non-Isocyanate Polyurethane-Based Composite Materials. Materials.

[56] Engels H.W., Pirkl H.G., Albers R., Albach R.W., Krause J., Hoffmann A., Dormish J. (2013). Polyurethanes: Versatile materials and sustainable problem solvers for today’s challenges. Angew. Chem. Int. Ed..

[57] Bähr M., Bitto A., Mülhaupt R. (2012). Cyclic limonene dicarbonate as a new monomer for non-isocyanate oligo-and polyurethanes (NIPU) based upon terpenes. Green Chem..

[58] Vanaraj R., Suresh Kumar S.M., Kim S.C., Santhamoorthy M. (2025). A Review on Sustainable Upcycling of Plastic Waste through Depolymerization into High-Value Monomer. Processes.

[59] Grand View Research Polyurethane Market Size, Share and Trends Analysis Report By Product (Rigid Foam, Flexible Foam), By Application (Construction, Furniture and Interiors), By Region, And Segment Forecasts, 2022–2030. https://www.grandviewresearch.com/industry-analysis/polyurethane-pu-market.

[60] Xia L., Gui T., Wang J., Tian H., Wang Y., Ning L., Wu L. (2025). Bio-Based Coatings: Progress, Challenges and Future Perspectives. Polymers.

[61] Vencorex Tolonate™ X FLO 100 Product Information. https://www.vencorex.com.

[62] Grdadolnik M., Zdovc B., Drinčić A., Onder O.C., Utroša P., Ramos S.G. (2023). Chemical Recycling of Flexible Polyurethane Foams by Aminolysis to Recover High-Quality Polyols. ACS Sustain. Chem. Eng..

[63] ICE-Belgium STABIO™ Polyisocyanates—Technical Data Sheet. https://cms.ice.be/files/249/tds-stabio-presentation-.pdf.

[64] Desai Y., Jariwala S., Gupta R.K. (2023). Polyurethanes: Preparation, Properties, and Applications Volume 2: Advanced Applications. American Chemical Society.

[65] Acik G., Karabulut H.R.F., Altinkok C., Karatavuk A.O. (2019). Synthesis and characterization of biodegradable polyurethanes made from cholic acid and l-lysine diisocyanate ethyl ester. Polym. Degrad. Stab..

[66] Niesiobędzka J., Datta J. (2023). Challenges and recent advances in bio-based isocyanate production. Green Chem..

[67] Fu C., Zheng Z., Yang Z., Chen Y., Shen L. (2014). A fully bio-based waterborne polyurethane dispersion from vegetable oils: From synthesis of precursors by thiol-ene reaction to study of final material. Prog. Org. Coat..

[68] Srivastava A., Maity S., Das B.R. (2025). A review on multi-functional polyurethane (PU) coatings for fabric applications: Materials, processes and recent developments. Prog. Org. Coat..

[69] Hojabri L., Kong X., Narine S.S. (2010). Functional thermoplastics from linear diols and diisocyanates produced entirely from renewable lipid sources. Biomacromolecules.

[70] Cifarelli A., Boggioni L., Vignali A., Tritto I., Bertini F., Losio S. (2021). Flexible polyurethane foams from epoxidized vegetable oils and a bio-based diisocyanate. Polymers.

[71] Akay O., Altinkok C., Acik G., Yuce H., Ege G.K. (2021). A bio-based and non-toxic polyurethane film derived from Luffa cylindrica cellulose and L-Lysine diisocyanate ethyl ester. Eur. Polym. J..

[72] Laurichesse S., Avérous L. (2014). Chemical modification of lignins: Towards biobased polymers. Prog. Polym. Sci..

[73] Chaoqun Z., Madbouly S.A., Kessler M.R. (2015). Biobased Polyurethanes Prepared from Different Vegetable Oils. ACS Appl. Mater. Interfaces.

[74] Das A., Mahanwar P. (2020). A brief discussion on advances in polyurethane applications. Adv. Ind. Eng. Polym. Res..

[75] SI Group Antioxidant Solutions for Polyurethanes and Polyols. https://discover.univarsolutions.com.

[76] Bhutra K., Datta S., More A.P. (2024). A Comprehensive Review on Biobased Hyperbranched Polymers. Polym. Bull..

[77] Makaveckas T., Šimonėlienė A., Šipailaitė-Ramoškienė V. (2025). Lignin Valorization from Lignocellulosic Biomass. Extraction, Depolymerization, and Applications in the Circular Bioeconomy. Sustainability.

[78] Georgs V., Piili H., Gustafsson J., Xu C. (2025). A critical review on lignin structure, chemistry, and modification towards utilisation in additive manufacturing of lignin-based composites. Ind. Crop. Prod..

[79] Dandash A.A., Abu-Jdayil B., Tannous J.H. (2025). Lignin Extraction from Various Biomass Sources: A Comprehensive Review of Characteristics, Applications, and Future Prospects. Chem. Rec..

[80] El Bouhali A., Gnanasekar P., Habibi Y. (2021). Chemical modifications of lignin. Lignin-Based Materials for Biomedical Applications: Preparation, Characterization, and Implementation.

[81] Glasser W.G., Hsu O.H., Reed D.L., Forte R.C., Wu L.F. (1981). Lignin-Derived Polyols, Polyisocyanates, and Polyurethanes. Urethane Chemistry and Applications.

[82] Watkins D., Nuruddin M., Hosur M., Tcherbi-Narteh A., Jeelani S. (2015). Extraction and characterization of lignin from different biomass resources. J. Mater. Res. Technol..

[83] Kuhire S.S., Nagane S.S., Wadgaonkar P.P. (2017). Poly(ether urethane)s from aromatic diisocyanates based on lignin-derived phenolic acids. Polym. Int..

[84] Thakur V.K., Thakur M.K. (2015). Recent advances in green hydrogels from lignin: A review. Int. J. Biol. Macromol..

[85] Sun R.C. (2020). Lignin source and structural characterization. ChemSusChem.

[86] Konieczny J., Loos K. (2019). Green Polyurethanes from Renewable Isocyanates and Biobased White Dextrins. Polymers.

[87] Emrani J., Benrashid R., Mohtarami S., Fini E., Abu-Lebdeh T. (2018). Synthesis and Characterization of Bio-based Polyurethane Polymers. Am. J. Eng. Appl. Sci..

[88] Guan J., Sacks M.S., Beckman E.J., Wagner W.R. (2002). Synthesis, Characterization, and Cytocompatibility of Elastomeric, Biodegradable Poly(ester-urethane)ureas based on poly(caprolactone) and putrescine. J. Biomed. Mater. Res..

[89] Atmaca U. (2020). A novel approach for the synthesis of β-keto esters: One-pot reaction of carboxylic acids with chlorosulfonyl isocyanate. Arkivoc.

[90] Błażek K., Datta J. (2019). Renewable natural resources as green alternative substrates to obtain bio-based non-isocyanate polyurethanes-review. Crit. Rev. Environ. Sci. Technol..

[91] Belgacem M.N., Gandini A. (2008). Chapter 18—Surface Modification of Cellulose Fibres. Monomers, Polymers and Composites from Renewable Resources.

[92] Gandini A. (2008). Polymers from Renewable Resources: A Challenge for the Future of Macromolecular Materials. Macromolecules.

[93] Mosiewicki M.A., Aranguren M.I. (2013). A short review on novel biocomposites based on plant oil precursors. Eur. Polym. J..

[94] Garcon R., Clerk C., Gesson J.P., Bordado J., Nunes T., Caroco S., Rauter A.P. (2001). Synthesis of novel polyurethanes from sugars and 1, 6-hexamethylene diisocyanate. Carbohydr. Polym..

[95] Zenner M.D., Xia Y., Chen J.S., Kessler M.R. (2013). Polyurethanes from isosorbide-based diisocyanates. ChemSusChem.

[96] Brzoska J., Smorawska J., Głowińska E., Datta J. (2024). A green route for high-performance bio-based polyurethanes synthesized from modified bio-based isocyanates. Ind. Crop. Prod..

[97] Jia P.Y., Hu L.H., Shang Q.Q., Wang R., Zhang M., Zhou Y. (2017). Self-Plasticization of PVC materials viachemical modification of mannich base of cardanol butyl ether. ACS Sustain. Chem. Eng..

[98] Nair C.P.R., Bindu R.L., Joseph V.C. (1995). Cyanate esters based on cardanol modified-phenol-formaldehyde resins: Syntheses and thermal characteristics. J. Polym. Sci. Part A Polym. Chem..

[99] Sandhya T.E. (2003). Synthesis and Characterization of Aliphatic-Aromatic Polyesters. Ph.D. Thesis.

[100] Baron A., Rodriguez-Hernandez J., Ibarboure E., Derail C., Papon E. (2009). Adhesives based on polyurethane graft multiblock copolymers: Tack, rheology and first morphological analyses. Int. J. Adhes. Adhes..

[101] Brzeska J., Piotrowska-Kirschling A. (2021). A Brief Introduction to the Polyurethanes According to the Principles of Green Chemistry. Processes.

[102] Godinho B., Gama N., Barros-Timmons A., Ferreira A. (2021). Recycling of different types of polyurethane foam wastes via acidolysis to produce polyurethane coatings. Sustain. Mater. Technol..

[103] Azobuild Blowing Agents for Polyurethane Foams. https://www.azobuild.com.

[104] Pantone V., Annese C., Fusco C., Fini P., Nacci A., Russo A., D’Accolti L. (2017). One-Pot Conversion of Epoxidized Soybean Oil (ESO) into Soy-Based Polyurethanes by MoCl_2_O_2_ Catalysis. Molecules.

[105] Hebda E., Bukowczan A., Michałowski S., Pielichowski K. (2022). Flexible Polyurethane Foams Reinforced by Functionalized Polyhedral Oligomeric Silsesquioxanes: Structural Characteristics and Evaluation of Thermal/Flammability Properties. Polymers.

[106] Silva R., Barros-Timmons A., Quinteiro P. (2023). Life cycle assessment of fossil-and bio-based polyurethane foams: A review. J. Clean. Prod..

[107] Luo Y., Geng Z., Zhang W., He J., Yang R. (2023). Strategy for constructing phosphorus-based flame-retarded polyure-thane elastomers for advanced performance in long-term. Polymers.

[108] Wang X., Song L., Hu Y. (2021). Phosphorus-based flame retardants for polyurethanes: Synthesis and mechanistic studies. Materials and Chemistry of Flame-Retardant Polyurethanes Volume 2: Green Flame Retardants.

[109] Tang G., Liu X., Zhou L., Zhang P., Deng D., Jiang H. (2020). Steel slag waste combined with melamine pyrophosphate as a flame retardant for rigid polyurethane foams. Adv. Powder Technol..

[110] Ma Z., Zhang J., Liu L., Zheng H., Dai J., Tang L.C., Song P. (2022). A highly fire-retardant rigid polyurethane foam capable of fire-warning. Compos. Commun..

[111] Li J.-L., Gao C.T., Sun X., Peng S.G., Wang Y.W., Qin S.H. (2023). Synergistic flame-retardant effects of aluminum diethyl phosphinate in PP/IFR system and the Flame-Retardant Mechanism. Int. Polym. Process..

[112] Nabipour H., Wang X., Song L., Hu Y. (2020). A fully bio-based coating made from alginate, chitosan and hydroxyapatite for protecting flexible polyurethane foam from fire. Carbohydr. Polym..

[113] Vega-Baudrit J., Delgado-Montero K., Madrigal-Carballo S. (2012). Biodegradable polyurethanes from sugar cane biowastes. Cellul. Chem. Technol..

[114] Xiao T., Wu L., Xu Q., Yu X., Fu Q., Zhang F., Li Y., Yin G., Huang L., Fatehi P. (2025). Highly-Flame-Retardant Performance and Sustainable Polyurethane Foams from Industrial Kraft Lignin via Exploiting Lignin Demethylation. Biomacromolecules.

[115] Wang Y., Zheng X., Jiang K., Han D., Zhang Q. (2024). Bio-based melamine formaldehyde resins for flame-retardant polyurethane foams. Int. J. Biol. Macromol..

[116] Zemła M., Prociak A., Michałowski S. (2022). Bio-Based Rigid Polyurethane Foams Modified with Phosphorus Flame Retardants. Polymers.

[117] Vahabi H., Gholami F., Tomas M., Movahedifar E., Yazdi M.K., Saeb M.R. (2024). Hydrogel and aerogel-based flame-retardant polymeric materials: A review. J. Vinyl Addit. Technol..

[118] Heidarian M., Shishesaz M.R., Kassiriha S.M., Nematollahi M. (2010). Characterization of structure and corrosion resistivity of polyurethane/organoclay nanocomposite coatings prepared through an ultrasonication assisted process. Prog. Org. Coat..

[119] Fuensanta M., Martín-Martínez J.M. (2019). Thermoplastic polyurethane pressure sensitive adhesives made with mixtures of polypropylene glycols of different molecular weights. Int. J. Adhes. Adhes..

[120] Akram N., Gurney R.S., Zuber M., Ishaq M., Keddie J.L. (2013). Influence of polyol molecular weight and type on the tack and peel properties of waterborne polyurethane pressure-sensitive adhesives. Macromol. React. Eng..

[121] Yousif E., Haddad R. (2013). Photodegradation and photostabilization of polymers, especially polystyrene. SpringerPlus.

[122] Wellt Chemicals Hindered Amine Light Stabilizer (HALS): Protecting Polymers from Degradation. https://welltchemicals.com.

[123] Wypych G. (2020). Handbook of UV Degradation and Stabilization.

[124] Rahman M., Brazel C.S. (2004). The plasticizer market: An assessment of traditional plasticizers and research trends to meet new challenges. Prog. Polym. Sci..

[125] Hepburn C. (1992). Polyurethane Elastomers.

[126] Cognard P. (2005). Handbook of Adhesives and Sealants: Basic Concepts and High Tech Bonding.

[127] Nguyễn T., Myer K. (2002). Organic Coatings. Handbook of Materials Selection.

[128] Wang C., Murugadoss V., Kong J., He Z., Mai X., Shao Q., Guo Z. (2018). Overview of carbon nanostructures and nano-composites for electromagnetic wave shielding. Carbon.

[129] Cui J., Xu J., Li J., Qiu H., Zheng S., Yang J. (2020). A crosslinkable graphene oxide in waterborne polyurethane anticorrosive coatings: Experiments and simulation. Compos. Part B Eng..

[130] Chattopadhyay D.K., Raju K.V.S.N. (2007). Structural Engineering of Polyurethane coatings for high performance applica-tions. Prog. Polym. Sci..

[131] Li Y., Luo X., Hu S. (2015). Bio-Based Polyols and Polyurethanes.

[132] Chaudhary M.L., Gupta R.K. (2025). Environmental and Health Concerns in Polyurethane. Non-Isocyanate Polyurethanes: Chemistry, Progress, and Challenges.

[133] Morales-Cerrada R., Tavernier R., Caillol S. (2021). Fully Bio-Based Thermosetting Polyurethanes from Bio-Based Polyols and Isocyanates. Polymers.

[134] Simon J., Barla F., Kelemen-Haller A., Farkas F., Kraxner M. (1988). Thermal stability of polyurethanes. Chromatographia.

[135] Simón D., Borreguero A.M., De Lucas A., Rodríguez J.F. (2018). Recycling of Polyurethanes from Laboratory to Industry, a Journey towards the Sutainability. Waste Manag..

[136] Datta J., Głowińska E., Włoch M. (2018). Mechanical Recycling via Regrinding, Rebonding, Adhesive Pressing and Mold-ing. Recycling of Polyurethane Foams.

[137] Guo L., Wang W., Guo X. (2022). Recycling of flexible polyurethane foams by regrinding scraps into powder to replace polyol for re-foaming. Materials.

[138] de la Cruz-Martínez F., Martínez de Sarasa Buchaca M., Martínez J., Fernández-Baeza J., Sánchez-Barba L.F., Rodríguez-Diéguez A., Lara-Sánchez A. (2019). Synthesis of bio-derived cyclic carbonates from renewable resources. ACS Sustain. Chem. Eng..

[139] North M., Pasquale R., Young C. (2010). Synthesis of cyclic carbonates from epoxides and carbon dioxide. Green Chem..

[140] Šooš Ľ., Matúš M., Legutko S., Bábics J. (2025). Research into Efficient Technology for Material Recovery of Waste Polyu-rethane Foams. Recycling.

[141] Shaikh A.A.G., Sivaram S. (1996). Organic carbonates. Chem. Rev..

[142] Pęczek E., Pamuła R., Białowiec A. (2024). Recycled Waste as Polyurethane Additives or Fillers. Materials.

[143] Deng Y., Dewil R., Appels L., Ansart R., Baeyens J., Kang Q. (2021). Reviewing the thermo-chemical recycling of waste polyurethane foam. J. Environ. Manag..

[144] Peti D., Dobránsky J., Michalík P. (2025). Recent Advances in Polymer Recycling: A Review of Chemical and Biological Processes for sustainable solutions. Polymers.

[145] Horváth T., Kecskés K., Jordán Csábrádiné A., Szőri-Dorogházi E., Viskolcz B., Szőri M. (2024). Searching for the Achil-les’ Heel of Urethane Linkage—An Energetic Perspective. Polymers.

[146] Zhou W., Neumann P., Al Batal M., Rominger F., Hashmi A.S.K., Schaub T. (2020). Depolymerization of Technical Grade Polyamide 66 and Polyurethane Materials via Hydrogenation. ChemSusChem.

[147] Ragaert K., Delva L., Van Geem K. (2017). Mechanical and Chemical Recycling of Solid Plastic Waste. Waste Manag..

[148] Wieczorek K., Bukowski P., Stawiński K., Ryłko I. (2024). Recycling of Polyurethane Foams via Glycolysis: A Review. Materials.

[149] Siddiqui M.N., Redhwi H.H., Achilias D.S. (2012). Recycling of poly(ethylene terephthalate) waste through methanolic pyrolysis in a microwave reactor. J. Anal. Appl. Pyrolysis.

[150] Lalhmangaihzuala S., Laldinpuii Z., Lalmuanpuia C., Vanlaldinpuia K. (2020). Glycolysis of poly(ethylene terephthalate) using biomass-waste derived recyclable heterogeneous catalyst. Polymers.

[151] Ghaderian A., Haghighi A.H., Taromi F.A., Abdeen Z., Boroomand A., Taheri S.M.R. (2015). Characterization of Rigid Polyurethane Foam Prepared from Recycling of PET Waste. Period. Polytech. Chem. Eng..

[152] Senra E.M., Silva A.L., Pacheco E.B. (2023). A review of waterborne polyurethane coatings and adhesives with polyester polyol from poly(ethylene terephthalate) waste. J. Polym. Environ..

[153] Al-Sabagh A.M., Yehia F.Z., Eshaq G., Rabie A.M., ElMetwally A.E. (2016). Greener Routes for Recycling of Poly(ethylene terephthalate). Egypt. J. Pet..

[154] Grignard B., Thomassin J.M., Gennen S., Poussard L., Bonnaud L., Raquez J.M., Detrembleur C. (2016). CO_2_-Blown Microcellular Non-Isocyanate Polyurethane (NIPU) Foams from bio-and CO_2_-sourced monomers to potentially thermal insulating materials. Green Chem..

[155] Bakkali-Hassani C., Berne D., Ladmiral V., Caillol S. (2022). Transcarbamoylation in polyurethanes: Underestimated exchange reactions. Macromolecules.

[156] Kathalewar M.S., Joshi P.B., Sabnis A.S., Malshe V.C. (2013). Non-Isocyanate Polyurethanes: From Chemistry to Applications. Rsc Adv..

[157] Das S., Klinedinst D.B., Yilgor I., Beyer F.L., Toki S., Hsiao B.S., Wilkes G.L. (2007). Structure-Property Relationships of Segmented Polyurethanes and Polyureas Based on Single Isocyanate Molecules as Hard Segments. Polym. Prepr..

[158] Kirchberg A., Khabazian Esfahani M., Röpert M.C., Wilhelm M., Meier M.A. (2022). Sustainable Synthesis of Non-Isocyanate Polyurethanes Based on Renewable 2,3-Butanediol. Macromol. Chem. Phys..

[159] El Mejjatti A., Harit T., Riahi A., Khiari R., Bouabdallah I., Malek F. (2014). Chemical Recycling of Polyethylene Terephthalate: Application to the Synthesis of Multiblock Copolyesters. Express Polym. Lett..

[160] Warsahartana H., Bashir A., Keyworth A., Davies R., Falkowska M., Asuquo E., Garforth A. (2023). Catalytic Steam Hydrolysis of Polyethylene Terephthalate to Terephthalic Acid Followed by Repolymerisation. Chem. Eng. Trans..

[161] Jia Z., Gao L., Qin L., Yin J. (2023). Chemical recycling of PET to value-added products. RSC Sustain..

[162] Mudondo J., Lee H.S., Jeong Y., Kim T.H., Kim S., Sung B.H., Kim H.T. (2022). Recent advances in the chemobiological upcycling of polyethylene terephthalate (PET) into value-added chemicals. J. Microbiol. Biotechnol..

[163] Salas R., Villa R., Velasco F., Macia M., Navarro V., Dupont J., Lozano P. (2025). On the Hydrolytic Depolymerization of Polyurethane Foam Waste by Ionic Liquids. Molecules.

[164] Nikje M.M.A., Nikrah M., Mohammadi F.H.A. (2008). Microwave-assisted Polyurethane Bond Cleavage via Hydroglycolysis Process at atmospheric pressure. J. Cell. Plast..

[165] He H.W., Hu H., Du K.M., Lu M., Yang F., Cui L.X., Wang X. (2025). Prospects of high-value recycling methods for polyurethane based on the selective cleavage of C–O/C–N bonds. Green Chem..

[166] Aksu Y., Haykiri-Acma H., Yaman S. (2025). Recycle of Flexible Polyurethane Foam by Acidolysis and Reuse of Recovered Polyol. J. Polym. Environ..

[167] Pu M., Fang C., Zhou X., Wang D., Lin Y., Lei W., Li L. (2024). Recent Advances in Environment-Friendly Polyurethanes from Polyols Recovered from the Recycling and Renewable Resources: A Review. Polymers.

[168] Gama N., Godinho B., Madureira P., Marques G., Barros-Timmons A., Ferreira A. (2024). Polyurethane Recycling Through Acidolysis: Current Status and Prospects for the Future. J Polym Environ.

[169] Gama N., Godinho B., Marques G., Silva R., Barros-Timmons A., Ferreira A. (2021). Recycling of polyurethane by aci-dolysis: The effect of reaction conditions on the properties of the recovered polyol. Polymer.

[170] Liu B., Westman Z., Richardson K., Lim D., Stottlemyer A.L., Farmer T., Gillis P., Hooshyar N., Vlcek V., Christopher P. (2024). Polyurethane foam chemical recycling: Fast acidolysis with maleic acid and full recovery of polyol. ACS Sustain. Chem. Eng..

[171] Dżerzdżon K., Datta J. (2025). Advances in the degradation and recycling of polyurethanes: Biodegradation strategies, MALDI applications, and environmental implications. Waste Manag..

[172] Rajan A., Ameen F., Jambulingam R., Shankar V. (2024). Biodegradation of polyurethane by fungi isolated from industrial wastewater—A sustainable approach to plastic waste management. Polymers.

[173] Magnin A., Pollet E., Perrin R., Ullmann C., Persillon C., Phalip V., Avérous L. (2019). Enzymatic recycling of thermoplastic polyurethanes: Synergistic effect of an esterase and an amidase and recovery of building blocks. Waste Manag..

[174] Tan T., Wang W., Zhang K., Zhan Z., Deng W., Zhang Q., Wang Y. (2022). Upcycling plastic wastes into value-added products by heterogeneous catalysis. ChemSusChem.

[175] Hartmann D., Rahman T., Carias L., Auad M.L., Adhikari S. (2024). Upcycling Polyurethane Plastics via Thermochemi-cal Conversion Pathways: A Comparison of Hydrothermal Liquefaction and Pyrolysis Processes. ACS Sustain. Chem. Eng..

[176] Sun B., Zou J., Qiu W., Tian S., Wang M., Tang H., Ma D. (2025). Chemical transformation of polyurethane into valuable polymers. Natl. Sci. Rev..

[177] Balu R., Dutta N.K., Roy Choudhury N. (2022). Plastic Waste Upcycling: A Sustainable Solution for Waste Management, Product Development, and Circular Economy. Polymers.

